# Resurrecting ancestral structural dynamics of an antiviral immune receptor: adaptive binding pocket reorganization repeatedly shifts RNA preference

**DOI:** 10.1186/s12862-016-0818-6

**Published:** 2016-11-08

**Authors:** Charles Pugh, Oralia Kolaczkowski, Austin Manny, Bryan Korithoski, Bryan Kolaczkowski

**Affiliations:** 1 Department of Microbiology & Cell Science and Institute for Food and Agricultural Sciences, University of Florida, Gainesville, USA; 2 Genetics Institute, University of Florida, Gainesville, USA

**Keywords:** RIG-I, RIG-like receptors, Ancestral reconstruction, Antiviral immunity, Evolution of immunity, Molecular evolution, Innate immunity

## Abstract

**Background:**

Although resurrecting ancestral proteins is a powerful tool for understanding the molecular-functional evolution of gene families, nearly all studies have examined proteins functioning in relatively stable biological processes. The extent to which more dynamic systems obey the same ‘rules’ governing stable processes is unclear. Here we present the first detailed investigation of the functional evolution of the RIG-like receptors (RLRs), a family of innate immune receptors that detect viral RNA in the cytoplasm.

**Results:**

Using kinetic binding assays and molecular dynamics simulations of ancestral proteins, we demonstrate how a small number of adaptive protein-coding changes repeatedly shifted the RNA preference of RLRs throughout animal evolution by reorganizing the shape and electrostatic distribution across the RNA binding pocket, altering the hydrogen bond network between the RLR and its RNA target. In contrast to observations of proteins involved in metabolism and development, we find that RLR-RNA preference ‘flip flopped’ between two functional states, and shifts in RNA preference were not always coupled to gene duplications or speciation events. We demonstrate at least one reversion of RLR-RNA preference from a derived to an ancestral function through a novel structural mechanism, indicating multiple structural implementations of similar functions.

**Conclusions:**

Our results suggest a model in which frequent shifts in selection pressures imposed by an evolutionary arms race preclude the long-term functional optimization observed in stable biological systems. As a result, the evolutionary dynamics of immune receptors may be less constrained by structural epistasis and historical contingency.

**Electronic supplementary material:**

The online version of this article (doi:10.1186/s12862-016-0818-6) contains supplementary material, which is available to authorized users.

## Background

Resurrection and biochemical analysis of ancestral proteins is a powerful technique for understanding the molecular-structural basis of functional evolution [1, 2]. Example studies have elucidated the precise details by which evolutionary changes in sequence produce changes in protein structure and how structural changes alter molecular function, generating a wealth of potentially generalizable results about how structural properties may affect the evolution of molecular function [3–18].

One of the major limitations of current ancestral sequence resurrection (ASR) studies is that they have—almost without exception—examined molecular systems that function in basic cellular processes and organism development, systems that are expected to be relatively slow-evolving [19–24]. In contrast, proteins comprising the immune systems of multicellular eukaryotes are often involved in evolutionary arms races with pathogens, leading to rapid and highly variable evolutionary trajectories [25–27]. Proteins directly interacting with pathogen factors are expected to evolve particularly rapidly, and genome-wide comparisons have generally supported this expectation [28–30]. No detailed ASR studies have yet been conducted on primary immune receptors, so we have little experimental information about how the evolution of structure-function occurs in these systems, particularly across large evolutionary timescales.

Another major limitation of existing ASR studies is that they have focused almost exclusively on proteins that function by binding small ligands such as sugars, hormones or other metabolites [8, 13, 16–18, 31, 32] or by light activation [12, 15]. Short-term evolution of macromolecular interactions has been examined through experimental evolution and studies of protein affinity maturation [33–36], and a few ASR studies have begun investigating the evolution of protein interactions with larger macromolecules [37–40]. However, the long-term molecular evolution of macromolecular interactions remains under-studied. Structural properties important for the evolution of macromolecular interactions may be different from those driving protein-small ligand interactions, and the extent to which results obtained in one case can be generalized to the other is not clear [41, 42].

The RIG-like receptors, RIG-I (DDx58) MDA5 (IFIH1) and LGP2 (DHx58), form a complement of cytoplasmic RNA-binding proteins contributing to innate antiviral immunity in a wide variety of vertebrates, including all mammals [43–46]. RIG-like receptors (RLRs) bind viral-derived RNAs and signal cellular immune responses, primarily through direct interactions with the mitochondrion-anchored signal transducer, IPS1 [47, 48] (Additional file 1: Figure S1).

In order to function as effective antiviral receptors, RLRs must collectively recognize a variety of viral-associated RNA types without binding the specific motifs associated with cytoplasmic host RNAs. Although the precise ligand complement of human and other vertebrate RLRs has not been determined, RLRs have been shown to recognize specific end structures of RNA molecules via a C-terminal RNA recognition domain (RD), providing a structural mechanism capable of differentiating viral-derived from host RNA [49–51]. An upstream helicase and intervening pincer domain also contribute to RLR-RNA binding, primarily through interactions with the RNA backbone [52].

RIG-I—by far the best understood of the RLRs—recognizes single- and double-stranded RNA molecules with and without 5′ triphosphate (5′ppp) moieties but does not recognize the 7-methylguanylate cap typical of eukaryote mRNA [52–54] Additionally, RIG-I exhibits severely reduced immune signaling activity from dsRNA with 3′ or 5′ overhangs, which are typical of mature host tRNAs, rRNAs and microRNAs [55–57]. Less is known about the specific RNA ligands bound by MDA5 and LGP2. LGP2 appears to bind RNA moieties similar to those of RIG-I as well as additional RNA end structures [58, 59]. The natural MDA5 ligands have remained the most mysterious, although evidence suggests that MDA5 may cooperatively bind long—and possibly short—blunt-ended dsRNA molecules or other specific virus-produced RNAs [59–61].

We have recently found that RLRs were present in the earliest multicellular animals and functionally diversified through a series of gene duplication events [46]. Our analysis uncovered evidence for recurrent protein-coding adaptation in RLRs throughout the mammalian lineage and in the human population, particularly targeting the RNA recognition domain, consistent with an ‘arms race’ model in which RLRs adapt their RNA-binding repertoire in response to rapidly-evolving viral threats.

In our view, the RLRs provide an excellent model with which to begin extending current ASR results obtained from slow-evolving small-ligand-binding proteins to fast-evolving immune receptors interacting with larger macromolecules. Better understanding the evolutionary trajectories leading to RLR functional diversity is expected to shed light on how pathogen-driven arms races can shape the molecular functions of primary immune receptors and how structural features of the receptors affect potential evolutionary trajectories.

## Results and discussion

The aim of this study is to characterize how RNA preference changed during early RIG-like receptor (RLR) evolution. We employed an approach beginning with phylogenetic analysis to establish the RLR protein family tree, followed by analysis of protein-coding adaptation to identify potential functional shifts in RNA binding. Functional shifts were confirmed using ancestral sequence reconstruction and kinetic analyses of ancestral proteins. We used molecular dynamics simulations of ancestral proteins to characterize the likely mechanisms driving observed shifts in RNA preference and confirmed these by measuring the RNA-binding kinetics of mutant ancestral proteins incorporating historical amino-acid substitutions. This strategy represents a general approach for examining the mechanistic basis for molecular-functional evolution in protein families [62] (see Methods for details).

### RIG-like receptors (RLRs) arose in early animals and diversified by gene duplication and protein-coding adaptation targeting the RNA recognition domain (RD) in deuterostomes and jawed vertebrates

**Fig. 1:**
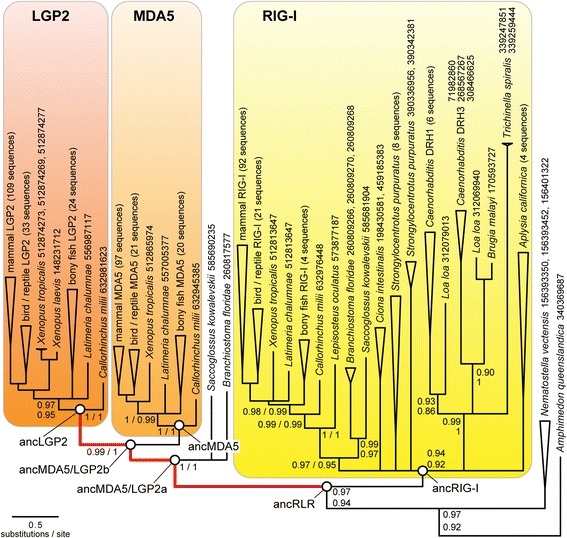
RIG-like receptors (RLRs) arose in early animals and diversified by gene duplications in bilateria and jawed vertebrates. We reconstructed the RLR gene phylogeny by maximum likelihood using two different alignments of all available RLR protein sequences (see Methods). Branch lengths are scaled to the average number of substitutions/site. We plot SH-like aLRT clade support from PROBALIGN (*top*) and MAFFT (*bottom*) alignments; clades with <0.8 support are collapsed to polytomies. Sequences from major monophyletic taxonomic groups are collapsed, and genbank IDs are provided for sequences from individual species. *Bold red* branches indicate significant support for protein-coding adaptation specific to the RNA-recognition (RD) domain (*p* < 0.01 after correction for multiple tests; see Methods). *White circles* indicate ancestral proteins resurrected in this study

In order to establish a robust framework within which to examine RIG-like receptor (RLR) functional evolution, we reconstructed the phylogeny of full-length RLR protein sequences using maximum likelihood, assuming two different alignments in order to incorporate alignment uncertainty (see Methods; Additional file 1: Table S1). Consistent with our previous study [46], the consensus tree across different alignments suggested the RLRs originated in the earliest multicellular animals and diversified by two major gene duplication events, one in bilateria and the other early in the jawed vertebrate lineage (Fig. 1). All bilaterian RLRs formed a monophyletic clade separated from *Amphimedon* and *Nematostella* RLRs with ≥0.94 SH-like aLRT, depending on the alignment. Our analysis confidently grouped deuterostome and protostome RIG-Is (support ≥0.92), suggesting that the earliest RLR gene duplication occurred before the protostome-deuterostome split. Vertebrate MDA5 and LGP2 sequences—along with *Saccoglossus* and *Branchiostoma* MDA5/LGP2—were monophyletic with maximal support, placing the MDA5-LGP2 duplication very early in the jawed vertebrate lineage, consistent with our previous findings.

The phylogeny reconstructed using sequence data implies a loss of MDA5/LGP2 in all protostomes and a second loss of RIG-I in arthropods (Additional file 1: Figure S2). The alternative phylogeny, in which the first RLR gene duplication occurred in deuterostomes after the protostome-deuterostome split, is more parsimonious in gene loss events—requiring only a single loss of the ancestral RLR in arthropods—but is consistently rejected by phylogenetic analysis (SH-test *p* < 0.023; see also [46]). Although it is impossible to completely rule out phylogenetic error, we have not observed any evidence for long-branch attraction or other systematic artifacts; in our previous analysis, the same phylogeny was consistently recovered using various alignments, inference methods, inclusion/exclusion of fast-evolving species and inclusion/exclusion of outgroup sequences [46]. The resolution of our previous phylogeny using additional sequence data and new alignments further supports its general robustness.

Although the evolution of a protein family’s molecular function can occur in the absence of adaptation or positive selection, reliable identification of protein-coding adaptation is a strong predictor of changes in molecular function, as adaptive processes can only occur when there is some phenotypic change visible to selection. We investigated the evolutionary forces driving RLR functional divergence using a branch-sites test to identify protein-coding adaptation in specific functional domains across specific branches on the phylogeny (see Methods). After correcting for multiple tests, we found strong support for protein-coding adaptation in one lineage following each of the major RLR duplication events (Fig. 1). In all cases, adaptation was specific to the C-terminal RNA recognition domain (RD) and was not found affecting any other functional domain (Additional file 1: Figure S3). These results are generally consistent with our previous findings of recurrent protein-coding adaptation in mammalian and human RLR RDs [46].

Following the earliest duplication of the ancestral RLR (ancRLR), protein-coding adaptation specific to the RD was detected along the branch leading to the first ancestral MDA5/LGP2 (ancMDA5/LGP2a) but not along that leading to ancRIG-I. We observed evidence for continued adaptation of the RD along the ancestral MDA5/LGP2 branch before the MDA5-LGP2 duplication and again in the LGP2 lineage after the MDA5-LGP2 split (Fig. 1). We did observe evidence for protein-coding adaptation in the helicase, pincer, CARD signaling domains and other regions of the RLR protein sequence following the initial diversification of RIG-I, MDA5 and LGP2 lineages (Additional file 1: Figure S3). However, only the RD was inferred to have evolved adaptively along the earliest branches of the RLR tree prior to the establishment of these three major RLR lineages.

Although the branch-sites test is generally considered robust [63–66], concerns have been raised about potentially high false-positive rates under some conditions [67, 68]. However, false-positive rates were always <0.05 when we simulated codon sequences along the maximum-likelihood RLR phylogeny using a variety of empirically-derived neutral scenarios (Additional file 1: Figure S4). Although it is impossible to completely rule out false-positive detection of protein-coding adaptation, we observed no evidence suggesting inflated false-positive rates in this case.

Consistent with evidence from analyses of protein-coding adaptation, we found that the branch lengths—measuring the expected number of protein substitutions/site—were generally longer on the earliest branches of the RLR phylogeny when optimized using only RD sequences, whereas later branches had longer lengths when optimized using the helicase + pincer domains, compared to the RD (Additional file 1: Figure S5). Together, these findings suggest that the C-terminal RNA recognition domain (RD) tended to evolve faster during the earlier history of RLR evolution, whereas the helicase and pincer domains exhibited faster protein-coding evolution only after the major RIG-I, MDA5 and LGP2 lineages were established.

The helicase, pincer and RD domains cooperatively bind viral RNA ligands, with the RD specifically recognizing the end structure of the RNA and the helicase + pincer interacting primarily with the RNA backbone [52]. Our results suggest that early functional evolution of RLR-RNA recognition may have occurred primarily through adaptively-driven changes in the RD, whereas later changes in RLR-RNA binding may have involved evolution of the helicase + pincer domains.

**Fig. 2:**
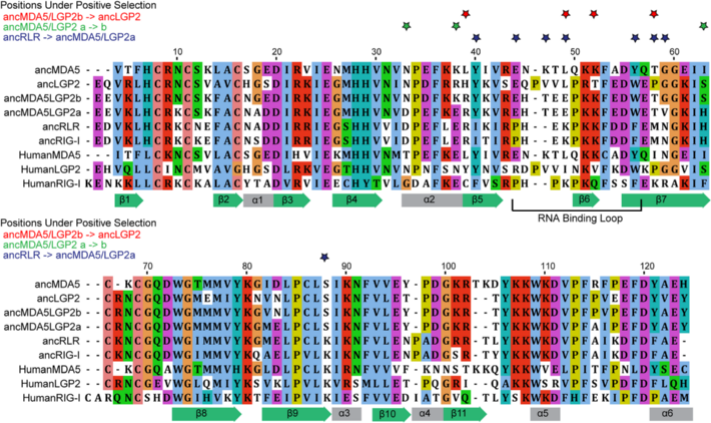
Adaptive protein-coding substitutions clustered near the RNA binding loop in the C-terminal RNA recognition domain (RD) along the lineages leading from ancRLR to ancLGP2 (see Fig. 1). We show the consensus sequence alignment of the three human RLR RDs and the ancestral RLRs resurrected in this study, with residues colored by biochemical classification and sequence conservation. Stars above the alignment indicate significant support for protein-coding adaptation at specific residues along the branches separating ancLGP2 from ancMDA5/LGP2b (*red*), ancMDA5/LGP2b from ancMDA5/LGP2a (*green*) and ancMDA5/LGP2a from ancRLR (*blue*), respectively (see Fig. 1). Adaptive substitutions were inferred by Bayes-empirical-Bayes posterior probability >0.95 using the branch-sites test for positive selection (see Methods). The location of the RNA binding loop is indicated, and approximate secondary structural elements are shown below the alignment

**Fig. 3:**
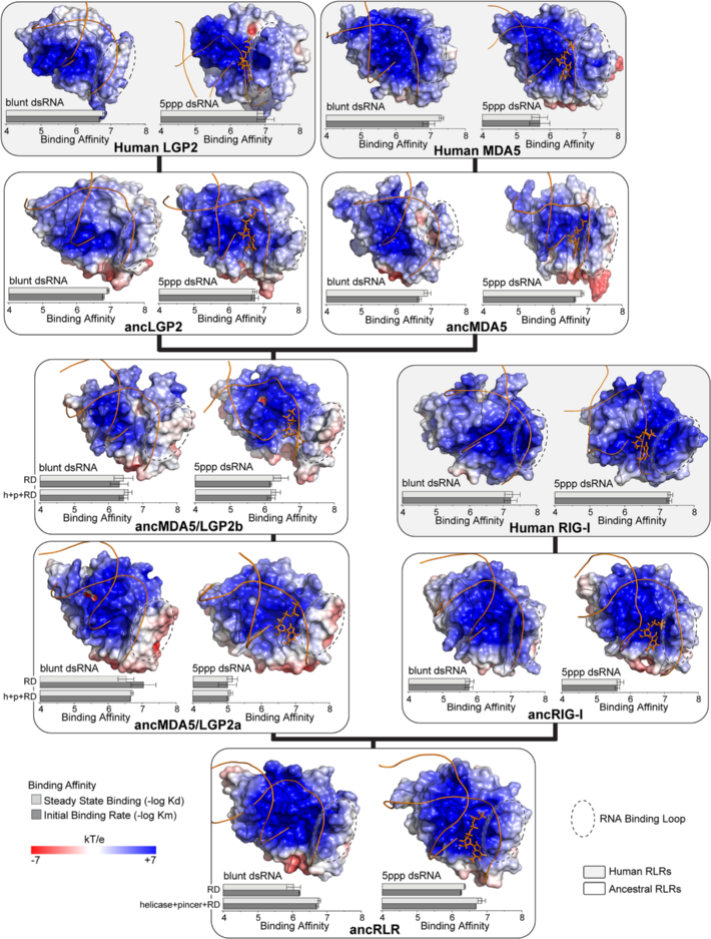
Adaptive substitutions alter RNA recognition domain (RD) structure and RNA-binding preference throughout RIG-like receptor (RLR) evolution. We inferred structural models of human and ancestral RLR RDs (see Figs. 1 and 2) bound to blunt-ended double-stranded RNA and dsRNA having a 5′ triphosphate (5ppp). We show the central structures of each RD-RNA from replicate molecular dynamics simulations, with electrostatic potential (kT/e) displayed on the molecular surface (see Methods). *Dotted ovals* indicate the location of the canonical RNA binding loop on each structural model. We resurrected ancestral RLR RDs and measured steady-state (Kd) and initial (Km) RNA binding affinities (see Methods). We plot –log-transformed binding affinities, with longer bars indicating higher affinity. Standard errors over three replicates are indicated. For ancRLR, ancMDA5/LGP2a and ancMDA5/LGP2b, we compare RNA binding affinities measured for the RD to affinities measured using the combined helicase + pincer + RD domains

To examine the early functional evolution of RLR-RNA recognition in more detail, we reconstructed ancestral protein sequences at key early nodes on the phylogeny (Fig. 1), inferred structural models of ancestral sequences (see Methods) and mapped putatively-adaptive substitutions to specific locations on the protein sequence (Fig. 2). In general, we found that inferred adaptive substitutions tended to cluster around the canonical “RNA-binding loop,” a flexible region of the RD that anchors the RNA ligand and makes key polar contacts with 5′ppp moieties in human RIG-I [53, 69]. Consistent with a potential functional role for adaptive changes in the RNA-binding loop, we observed a shift in the electrostatic distribution of the RNA-binding loop from strongly basic in ancRLR, ancRIG-I and human RIG-I to strongly acidic in ancMDA5/LGP2a and b (Fig. 3). The evolution of an acidic ‘RNA-binding loop’ in ancMDA5/LGP2a is expected to alter key electrostatic interactions likely to be important for RNA binding [46, 69, 70].

**Fig. 4:**
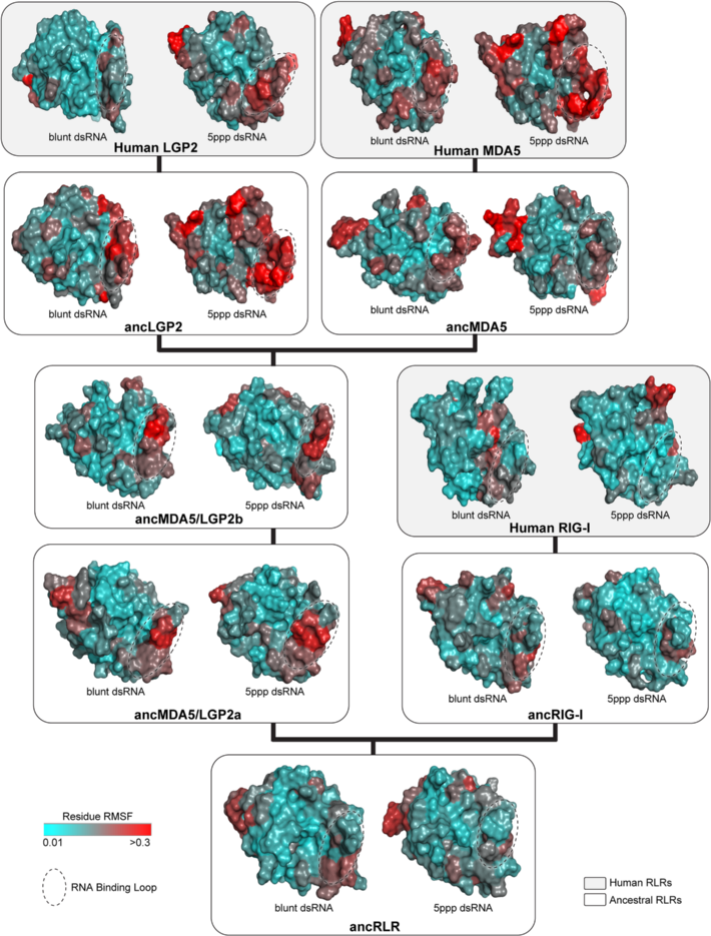
The RNA-binding loop of ancRLR and the RIG-I lineage fluctuates less over the course of molecular dynamics simulations, compared to the RNA-binding loop of other ancestral and human RLRs. We plot the root mean square fluctuation (RMSF) of each residue on the molecular surface of ancestral (*white boxes*) and human (*gray boxes*) RLR RNA-recognition domains (RD), averaged over replicate molecular dynamics simulations (see Methods). Higher values of RMSF indicate that the residue moved more over the course of the dynamics simulations. *Dotted oval* indicates location of the RNA-binding loop on each structure. See Figs. 1 and 2 for locations of each ancestral RLR on the phylogeny and ancestral RD sequences, respectively

Also consistent with a potential role in altering RD-RNA interactions, we observed changes in the stability of the RNA-binding loop during molecular dynamics simulations of ancestral RDs bound to RNA (Fig. 4). Specifically, residues comprising the RNA-binding loop fluctuated very little over the course of molecular dynamics simulations of ancRLR, ancRIG-I and human RIG-I RDs bound to blunt-ended or 5′ppp double-stranded RNA ligands, suggesting that these residues are likely stabilized by strong interactions with the RNA. Other ancestral RLRs in the MDA5/LGP2 lineage exhibited much larger root mean square fluctuation (RMSF) of residues in the RNA-binding loop, suggesting a general loss of stabilizing RNA interactions along this lineage. We observed a similar increase in the fluctuation of the RNA-binding loop in ancMDA5/LGP2a and ancMDA5/LGP2b during molecular dynamics simulations of the combined helicase + pincer + RD domains, suggesting that this observation is likely relevant to general RLR-RNA interactions and not an artifact of simulating only the RD bound to RNA (Additional file 1: Figure S6). Our results are generally consistent with recent structural studies of chicken LGP2 and MDA5, which also found the ‘RNA-binding loop’ failed to make strong contacts with RNA ligands [59]. Overall, these results suggest that the RNA-binding loop lost the capacity to bind blunt-ended and 5′ppp dsRNA ligands early in the MDA5/LGP2 lineage following the first RLR gene duplication in bilateria, and this functional shift was likely retained throughout MDA5/LGP2 evolutionary history.

**Fig. 5:**
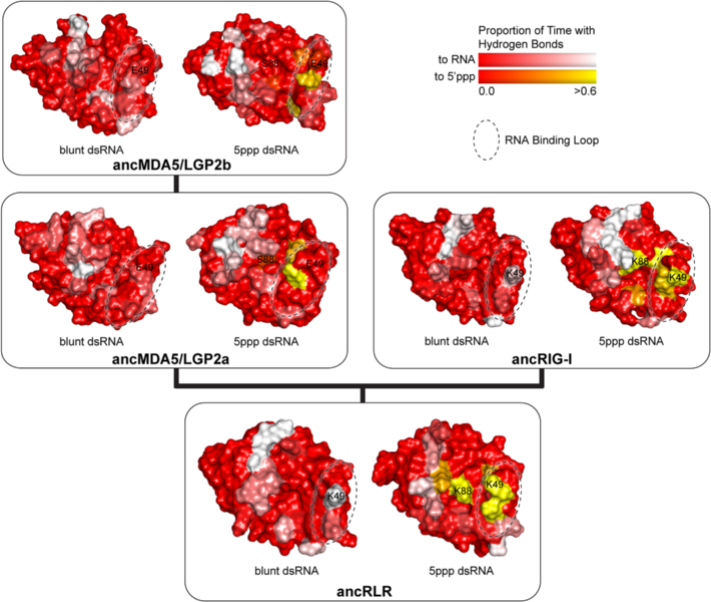
The RNA-binding loop loses hydrogen bonding to RNA ligands in the MDA5/LGP2 lineage after the first RLR gene duplication. We plot the proportion of molecular dynamics time points during which each residue was observed to form hydrogen bonds with the RNA ligand (*red-white gradient*) or the 5′ppp moiety in particular (*red-yellow gradient*), averaged over replicate simulations of ancestral RLR RDs (see Methods). The RNA-binding loop and specific residues exhibiting reduced hydrogen bonding to 5′ppp dsRNA in ancMDA5/LGP2a-b are indicated

Consistent with this general model, we found that the “RNA-binding loop” of ancMDA5/LGP2a and ancMDA5/LGP2b exhibited reduced capacity to form hydrogen bonds with RNA ligands over the course of molecular dynamics simulations, compared to ancRIG-I (Fig. 5; Additional file 1: Figure S7). Specifically, MDA5/LGP2a lost a key polar contact conserved in ancRLR and the RIG-I lineage (K49, see Fig. 2), which appears to stabilize both blunt-ended and 5′ppp dsRNA (Fig. 5). Additionally, K88 appears to form a strong hydrogen bond with the 5′ppp moiety in ancRLR and ancRIG-I, whereas the corresponding Ser in the ancMDA5/LGP2 lineage does not stabilize the 5′ppp dsRNA ligand (see Figs. 2 and 5). These losses of key polar contacts with the RNA ligand along the MDA5/LGP2 lineage were observed whether we simulated only the RD bound to RNA (Fig. 5) or the complete helicase + pincer + RD (Additional file 1: Figure S7), suggesting that adaptively-driven changes in the RLR RD may have altered the hydrogen bond network stabilizing RLR-RNA interactions, particularly early in the establishment of the MDA5/LGP2 lineage.

Maximum likelihood reconstructions of ancestral RD sequences were highly similar to those found in our previous study [46], and support for ancestral sequences was generally improved (Additional file 1: Figure S8). For each of the ancestral sequences reconstructed, > 47.5 % of ancestral states were reconstructed with >0.95 posterior probability; > 50.8 % of states were reconstructed with >0.9 posterior probability, and >65 % of states had >0.8 posterior probability. Only two sites in each of ancRLR, ancMDA5/LGP2a and ancMDA5/LGP2b had alternative reconstructions with posterior probability >0.3, and ancRIG-I had three such sites. All alternative reconstructions were considered biochemically conservative, and positions with plausible alternative reconstructions generally occurred in parts of the RD structure unlikely to strongly impact RNA binding (Additional file 1: Figure S8).

When we simulated sequence data using the maximum-likelihood tree and best-fit evolutionary model, error rates for ancRLR and ancRIG-I RD reconstructions were <0.03, with remaining ancestral sequences having error rates <0.02 (Additional file 1: Figure S8). Considering residues of the same general biochemical class as equivalent reduced all error rates to < 0.01. Ancestral reconstructions of the helicase + pincer domains were even more strongly supported than those of the RD (Additional file 1: Figure S9). The number of ancestral states across the helicase + pincer reconstructed with >0.9 posterior probability was always >53 %; fewer than 0.06 % of states had alternative reconstructions with posterior probability >0.3, and estimated error rates were always <0.03.

Ancestral sequences were reconstructed using full-length RLRs aligned by PROBALIGN, which produced slightly stronger support for the consensus phylogeny, compared to the MAFFT alignment (see Fig. 1). Reconstructing ancestral RLR RDs at key nodes on the phylogeny using the MAFFT alignment produced sequences >73 % identical (>79 % identical when considering biochemically similar residues as equivalent) to those produced from the PROBALIGN alignment, and the important sequence differences among ancestral RLR RDs outlined above were present in the alternative ancestral reconstructions (Additional file 1: Figure S10A).

We found ample sequence similarity between all ancestral RLR RDs and human RIG-I RD to support accurate structural homology modeling of ancestral proteins (48 % identity for ancRLR, 43 % for ancMDA5/LGP2a, 41 % for ancMDA5/LGP2b, 37 % for ancMDA5, 36 % for ancLGP2 and 49 % for ancRIG-I) [71–73]. Sequence identity between ancestral RLR helicase + pincer domains and human RIG-I helicase + pincer were similarly high (46 % for ancRLR, 37 % for ancMDA5/LGP2a and 36 % for ancMDA5/LGP2b). Consistent with expectation based on high sequence similarity of ancestral RLRs to the crystalized human RIG-I, objective measurements of structural modeling quality were generally high (Additional file 1: Table S2). Conducting replicate molecular dynamics simulations and examining the resulting ‘central’ structures (see Methods) further improved the quality of inferred structural models (Additional file 1: Table S2).

We ran molecular dynamics simulations for 11 ns, excluding the first nanosecond as ‘burnin’ (see Methods). All simulations of RDs bound to RNA appear to have reached stationarity following burnin (Additional file 1: Figures S11–S13). Energy, temperature and pressure parameter values fluctuated randomly around a relatively stable average (Additional file 1: Figure S11), as did the volume and density of the simulated solvent box (Additional file 1: Figure S12). The radius of gyration, root mean squared deviations and minimum atom-atom distances also exhibited little directional trend following burnin—although some simulations did exhibit directional trends during the burnin period—and the minimum atom-atom distance never fell below 2 nm (Additional file 1: Figure S13). Molecular dynamics simulations of combined helicase + pincer + RD domains bound to RNA exhibited similar evidence for stationarity (Additional file 1: Figures S14–S16). Best-fit linear regressions to each simulation time course series exhibited little deviation from zero slope. The average absolute-value of best-fit slope was 4.4e^−3^, the median was 2.1e^−5^ and the largest inferred slope was 7.6e^−2^ in absolute value.

Although it is not possible to completely rule out potential errors in ancestral sequence reconstruction, structural modeling or dynamics simulations, we did not observe any compelling evidence to suggest high potential for errors in these inferences. As a whole, our results suggest that the RLRs functionally diversified via gene duplications in bilateria and jawed vertebrates, associated with protein-coding adaptation specifically targeting residues in the RD likely affecting RNA binding by altering hydrogen bond networks.

### RIG-like receptors (RLRs) repeatedly lost and gained affinity for 5′-triphosphate (5′ppp) double-stranded RNA (dsRNA)

To examine the evolution of RNA preference across the early RLR phylogeny experimentally, we measured the affinity with which each ancestral RLR RD bound double-stranded RNAs (dsRNAs) having either a blunt end or a 5′-triphosphate (5′ppp) moiety (see Methods). These particular RNA types were chosen due to their association with viral infections [56, 74–79], observed differences in blunt-ended vs. 5′ppp dsRNA preference among human and ancestral RLRs [46, 52, 53, 80–83] and availability of crystal structures of human RIG-I bound to both RNAs to support structural modeling and molecular dynamics [51, 52, 84]. While we are aware that these two RNA types may not represent the primary drivers of historical RLR evolution, they do provide an important starting point from which to begin examining the evolution of RLR-RNA interactions (see Conclusions).

We observed a dramatic shift in RNA preference between the ancestral RLR (ancRLR) and the first ancestral MDA5/LGP2 after the bilaterian gene duplication (ancMDA5/LGP2a), followed by an immediate reversion back to the ancestral binding preference along the ancestral MDA5/LGP2 lineage (ancMDA5/LGP2b; Fig. 3; Additional file 1: Figures S10B, S17–S19). Consistent with our previous results [46], the ancestral RLR RD had equal preference for blunt-ended and 5′ppp dsRNA (*p* = 0.09 for steady-state affinities; *p* = 0.33 for initial binding rates), whereas ancMDA5/LGP2a RD evolved ~20-fold reduced affinity for 5′ppp dsRNA (25.8-fold decrease in steady-state affinity, *p* = 0.04; 18-fold decrease in initial binding rate, *p* = 0.014). The ~3-4-fold increase in affinity for blunt-ended dsRNA we observed along this branch was not significant (*p* > 0.15). In contrast, the RNA binding affinities of ancRIG-I RD were much more similar to those of the ancestral RLR and human RIG-I RD (*p* > 0.03; Additional file 1: Figures S17 and S18).

Surprisingly, we found that the RD of ancMDA5/LGP2b bound blunt-ended and 5′ppp dsRNAs with the same affinity, similar to what we observed for ancRLR RD and RDs in the RIG-I lineage (*p* > 0.64). This was caused by an observed ~20-fold increase in ancMDA5/LGP2b RD’s affinity for 5′ppp dsRNA, compared to the RD of ancMDA5/LGP2a (21-fold increase in steady state affinity, *p* = 0.04; 18.5-fold increase in initial binding rate, *p* = 0.01). Binding to both 5′ppp and blunt-ended RNA types by ancMDA5/LGP2b RD were equivalent to that of ancRLR RD, suggesting an evolutionary reversion to an ancestral function (*p* > 0.08 and *p* > 0.73, respectively). Ancestral RDs resurrected immediately following the MDA5-LGP2 duplication also bound blunt-ended and 5′ppp dsRNAs with equivalent affinities (*p* > 0.7), arguing against sequence reconstruction errors in ancMDA5/LGP2b as a major explanation of our results (Fig. 3; Additional file 1: Figures S17 and S18). Ancestral sequences reconstructed using an alternative sequence alignment exhibited the same evolutionary shifts in RNA preference, further supporting the robustness of our findings to ancestral reconstruction uncertainty (Additional file 1: Figure S10B).

Human LGP2 RD also bound both RNA types with equal affinity (*p* > 0.24), whereas human MDA5 RD appears to have re-evolved a preference for blunt-ended over 5′ppp dsRNA (24.7-fold higher steady-state preference for blunt-ended dsRNA, *p* = 0.03; 45.3-fold higher initial binding rate, *p* = 0.006), similar to what we observed for the RD of ancMDA5/LGP2a (Additional file 1: Figures S17 and S18). Previous studies have established that human LGP2 RD binds RNA molecules using structural mechanisms distinct from those of human RIG-I RD, despite having similar RNA preference [51, 61, 69, 85]. Our results suggest that the similarity in RNA preference between human LGP2 and RIG-I RDs originated prior to the MDA5-LGP2 duplication (in ancMDA5/LGP2b), although it did evolve from a (functionally) MDA5-like ancestor (ancMDA5/LGP2a). Our finding that human MDA5 prefers blunt-ended dsRNA over 5′ppp dsRNA is also consistent with recent analyses of chicken MDA5 [59].

Previous studies have shown that—in addition to the C-terminal RD—RLR helicase + pincer domains contribute to RNA binding and may affect RNA preference [52, 60, 61, 85]. To determine whether changes in ancestral RLR helicase + pincer contributed to the evolution of RNA preference in early animal RLRs, we compared the RNA preferences of ancestral RLR constructs encoding the complete helicase + pincer + RD domains to those of ancestral RDs, alone. We found that ancestral helicase + pincer + RDs exhibited the same loss of 5′ppp dsRNA affinity along the branch leading from ancRLR to ancMDA5/LGP2a, as well as the same reversion back to equal blunt-vs-5′ppp dsRNA affinities in ancMDA5/LGP2b (Fig. 3; Additional file 1: Figures S17–S19). Specifically, we observed a ~50-fold decrease in ancMDA5/LGP2a helicase + pincer + RD’s affinity for 5′ppp dsRNA, compared to ancRLR (*p* < 0.0002). AncMDA5/LGP2b helicase + pincer + RD displayed equal preference for 5′ppp and blunt-ended dsRNA (*p* > 0.58), similar to ancRLR (*p* > 0.42).

These results indicate that the binding shift we observed for ancestral RDs is not an artifact of measuring RD-RNA binding in the absence of other protein functional domains and suggest that changes in the helicase + pincer domain likely contributed little to the early evolution of RLR-RNA preference, at least for these two RNA types. However, later changes in the helicase + pincer may have impacted RLR-RNA binding of more recently-derived receptors. We did not examine the impact of ancestral RLR CARD domains on RNA binding, as RLR CARD sequences were too divergent to reliably reconstruct ancestral CARDs on these deep nodes of the phylogeny, and CARDs are not considered to strongly impact RNA binding in extant receptors [54, 86, 87].

Our examination of RNA preference across the early RLR phylogeny paints a general picture in which RLRs repeatedly gain and lose affinity for 5′ppp dsRNA, first by losing ancestral high-affinity after the first RLR duplication in deuterostomes, then by immediately re-gaining 5′ppp dsRNA affinity along the MDA5/LGP2 lineage and finally losing it again sometime after MDA5 diverged from LGP2 in jawed vertebrates. In contrast to what has been observed for proteins involved in metabolic and developmental processes, in which major changes in ligand preference generally occur following gene duplication or speciation events and are fairly conserved across large evolutionary distances [8, 13, 16, 23, 31, 88, 89], our results suggest that the evolution of ligand preference in immune receptors can occur much more rapidly, independently of gene duplications, and may repeatedly ‘flip flop’ between two or more functional states. In general, we would expect the strong and variable selection pressures exerted on immune receptors by fast-evolving pathogens to result in more rapid and variable evolution of receptor function, and our results are consistent with this expectation.

### A complex substitution in the RNA binding loop reduced affinity for 5′ppp dsRNA

**Fig. 6:**
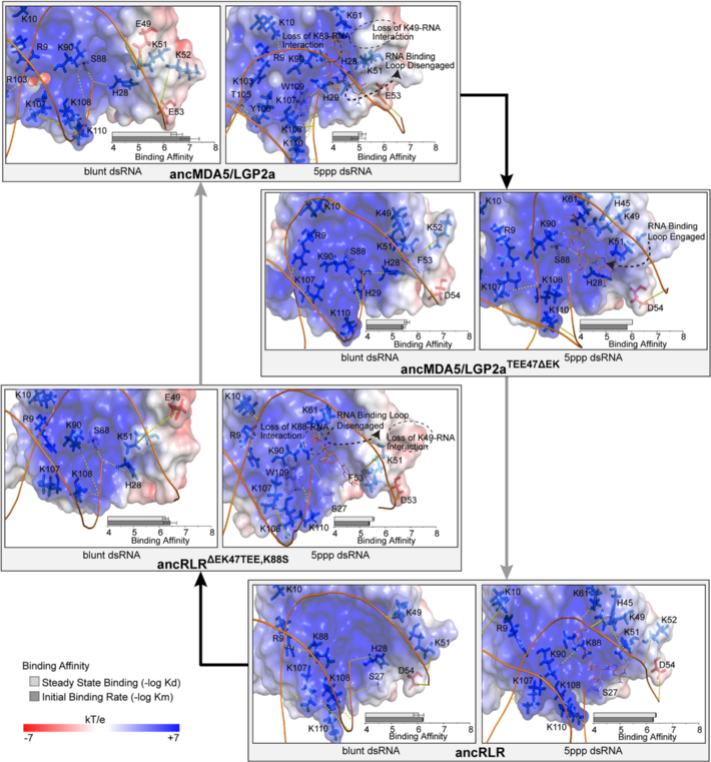
Two complex substitutions are sufficient to recapitulate the observed shift in RNA-binding preference following the earliest RLR gene duplication. We reconstructed ancestral RD protein sequences before and after the first RLR gene duplication (see Fig. 1), constructed structural models of RDs bound to blunt-ended and 5′ppp dsRNAs and performed replicate molecular dynamics simulations to characterize the structural basis for RNA binding (see Methods). We introduced the historical ΔEK47TEE and K88S substitutions into the ancRLR background as well as the ‘reverse’ TEE47ΔEK substitution into the ancMDA5/LGP2a background. We show the central structures of each RD-RNA interaction from replicate molecular dynamics simulations, with electrostatic potential (kT/e) displayed on the molecular surface. Residues forming hydrogen bonds to the RNA molecule in at least 50 % of sampled time points from molecular dynamics simulations (see Additional file 1: Figures S19 and S20) are shown as sticks, with *dashed yellow lines* indicating hydrogen bonds. We measured steady-state (Kd) and initial (Km) RNA-binding affinities of ancestral and mutant RDs bound to each RNA type (see Methods). We plot –log-transformed binding affinities, with longer bars indicating tighter affinity. Standard errors over three replicates are indicated

Integrating results from analysis of protein-coding adaptation (Figs. 1 and 2) and molecular dynamics (Figs. 3, 4 and 5), we hypothesized that two historical substitutions were primarily responsible for the observed loss of 5′ppp dsRNA binding between ancRLR and ancMDA5/LGP2a: a complex ΔEK47TEE substitution within the canonical RNA-binding loop and a second K88S substitution within the β9-α3 transition (Figs. 2 and 5). When we introduced these mutations into the ancRLR background (ancRLR^∆EK47TEE,K88S^), binding to 5′ppp dsRNA was reduced, similar to what we observed in ancMDA5/LGP2a (7.8-fold reduction in steady-state binding, *p* = 0.0008; 6.7-fold reduction in initial binding rate, *p* = 0.003; Fig. 6; Additional file 1: Figures S17 and S18), but binding to blunt-ended dsRNA was retained (*p* > 0.18). These results indicate that the combined ΔEK47TEE and K88S substitutions are sufficient to recapitulate the observed functional shift between ancRLR and ancMDA5/LGP2a. Introducing other historical substitutions around the RNA-binding pocket into the ancRLR background did not shift ancRLR’s RNA preference (*p* > 0.17; Additional file 1: Figure S17), suggesting that ΔEK47TEE and K88S played a specific role in reducing 5′ppp dsRNA binding during early RLR evolution.

Hydrogen bond networks are expected to be important for stabilizing protein-RNA interactions. Consistent with this expectation, we found that all ionizable residues potentially contacting RNA ligands were heavily protonated during molecular dynamics simulations (Additional file 1: Figure S20). Molecular dynamics simulations support the general conclusion that the ΔEK47TEE and K88S substitutions disrupted hydrogen bonding between the ancestral RLR RD and 5′ppp dsRNA (Additional file 1: Figure S21). Hydrogen bonding was reduced in the ancRLR^∆EK47TEE,K88S^ mutant, compared to the ancestral RLR, both to the 5′ppp dsRNA as a whole (*p* = 0.03) and to the 5′ppp moiety in particular (*p* = 0.02). We observed no differences in hydrogen bonding to 5′ppp dsRNA between the ancRLR^∆EK47TEE,K88S^ mutant and ancMDA5/LGP2a (*p* > 0.13), and no overall differences in hydrogen bonding to blunt-ended dsRNA were observed among any of the ancestral or mutant RDs (*p* > 0.23). These results suggest that the ancRLR^∆EK47TEE,K88S^ mutant is sufficient to recapitulate not only the observed functional shift in RNA preference but also the changes in hydrogen bond networks expected to be responsible for this functional shift.

Structural modeling and molecular dynamics suggest that the ΔEK47TEE substitution introduced an electrostatic clash between the canonical ‘RNA-binding loop’ and the large 5′ppp moiety, disrupting a number of favorable protein-5′pppRNA contacts but having minimal effect on interactions between the protein and blunt-ended dsRNA (Fig. 6). The K49E substitution directly replaces a strong polar contact between the protein and the 5′ppp with a repulsive acidic residue; K49 formed hydrogen bonds with the 5′ppp moiety in 56 % of the sampled time points during molecular dynamics simulations of ancRLR, whereas the corresponding 49E residue did not form any hydrogen bonds in either ancMDA5/LGP2a or the mutant ancRLR^∆EK47TEE,K88S^ (*p* < 0.002; Fig. 5; Additional file 1: Figure S22). The Δ47T insertion is expected to exert some force to reposition the acidic E48 closer to the bulky 5′ppp, further interfering with its ability to engage the ‘RNA-binding loop’ (Fig. 6).

The ancestral K88 formed hydrogen bonds with the 5′ppp moiety in 47 % of the molecular dynamics samples from ancRLR, whereas hydrogen bonds were formed between K88 and blunt-ended dsRNA in only 2 % of ancRLR dynamics simulation samples (*p* = 0.02; Additional file 1: Figure S20). In the case of ancMDA5/LGP2a, the corresponding 88S residue formed hydrogen bonds with 5′ppp 13 % of the time and hydrogen bonds with blunt-ended dsRNA 33 % of the time (*p* = 0.17; Fig. 5; Additional file 1: Figure S22). The ancRLR^∆EK47TEE,K88S^ mutant recapitulated this loss of 5′ppp-specific hydrogen bonding (*p* = 7.1e^−5^; Additional file 1: Figure S22), suggesting that the K88S substitution specifically stabilizes blunt dsRNA.

The combined effect of these functional substitutions was to shift the orientation of the 5′ppp dsRNA in the ligand-binding pocket away from its ancestral orientation with the 5′ppp moiety engaged in the RNA-binding loop (Fig. 6) and into an orientation more similar to that of the blunt-ended dsRNA (Fig. 6). In this new orientation, the bulky 5′ppp moiety clashes with the ‘acidified RNA-binding loop’ and is not stabilized by 88S (Fig. 6). The blunt-ended dsRNA lacks a bulky 5′ppp, reducing its clash with the ‘acidified RNA-binding loop’ and allowing 88S to stabilize the sugar backbone of the 5′ terminal base (Fig. 6).

Previous studies of slow-evolving receptors have revealed instances in which, after a functional shift, subsequent “restrictive” substitutions can occur that prevent the direct reversal of receptor preference [9, 90–92]. However, when we introduced the “reverse” TEE47∆EK substitution into the derived ancMDA5/LGP2a (ancMDA5/LGP2a^TEE47∆EK^), we observed a complete reversion to the ancestral ancRLR function, in which blunt-ended and 5′ppp dsRNAs are bound with equal affinity (*p* > 0.13; Fig. 6; Additional file 1: Figures S17 and S18). The “reverse” ancMDA5/LGP2a^TEE47∆EK^ mutant was also sufficient to revert overall hydrogen bonding to an ‘ancestral’ condition (Fig. 6; Additional file 1: Figures S21 and S22). The number of hydrogen bonds formed over molecular dynamics simulations was indistinguishable between ancRLR and ancMDA5/LGP2a^TEE47∆EK^, both to the RNA as a whole (*p* > 0.21) and to the 5′ppp moiety in particular (*p* = 0.13). Additionally, the 5′ppp dsRNA adopted the ancestral RLR conformation, with the 5′ppp moiety engaged in the RNA-binding loop (Fig. 6). These results suggest that the TEE47∆EK substitution may be primarily responsible for the differences in 5′ppp binding between ancRLR and ancMDA5/LGP2a, and that ‘restrictive’ substitutions required to optimize shifts in receptor function may be less likely in fast-evolving immune receptors than in more slowly-evolving systems.

In addition to changes in the hydrogen bond network, we also observed some significant differences in sidechain and RD backbone flexibility (Additional file 1: Figure S23), the distance between the RD and its bound ligand (Additional file 1: Figure S24), and protein secondary structure (Additional file 1: Figure S25) across molecular dynamics simulations of different ancestral and mutant RDs. These changes in other molecular-dynamics properties were largely consistent with inferred changes in hydrogen bonding networks, which appear sufficient to explain the observed differences in molecular binding kinetics.

### A single amino-acid substitution restored affinity for 5′ppp dsRNA

**Fig. 7:**
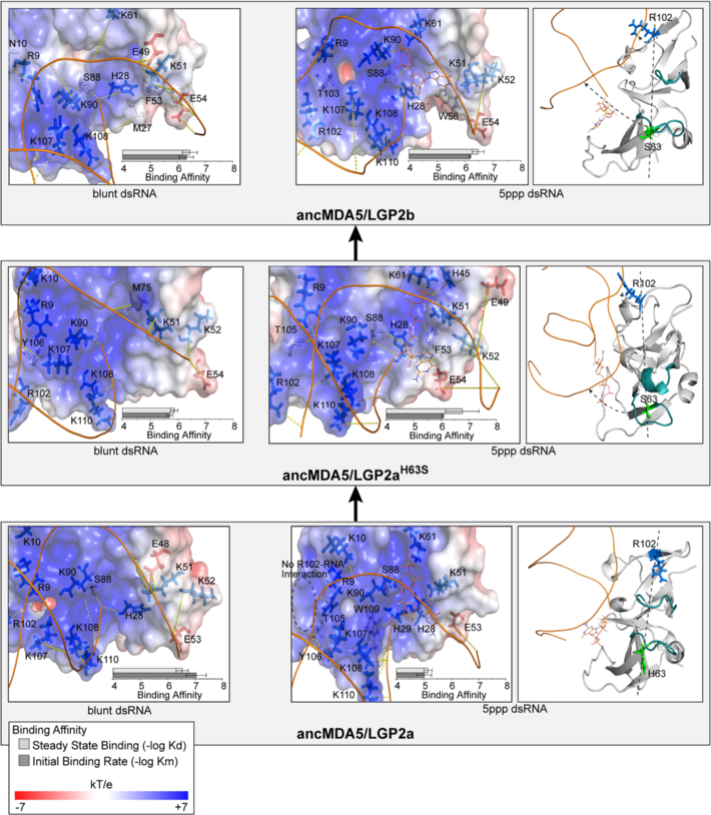
A single amino acid substitution is sufficient to recapitulate the observed re-evolution of high-affinity 5′ppp dsRNA binding between ancMDA5/LGP2a and ancMDA5/LGP2b. We reconstructed RD protein sequences of the first (ancMDA5/LGP2a) and last (ancMDA5/LGP2b) ancestral RLRs between the first and second major RLR gene duplications (see Fig. 1). We additionally introduced a single historical H63S substitution into the ancMDA5/LGP2a background. We show the central structures from replicate molecular dynamics simulations of each RD bound to blunt-ended and 5′ppp dsRNA (see Methods). Electrostatic potential (kT/e) is displayed across the molecular surface of each RNA-binding pocket (left panels). Residues forming hydrogen bonds to the RNA in at least 50 % of molecular dynamics time points (see Additional file 1: Figures S24 and S25) are shown as sticks, with *dashed yellow lines* indicating hydrogen bonds. We plot –log-transformed steady-state (Kd) and initial (Km) RD-RNA binding rates, with bars indicating standard errors. Right panels show each RD bound to 5′ppp dsRNA. A *dotted line* connects the H/S63 (*green*) and R102 (*blue*) residues and the zinc-binding pocket (teal)

Between the origin of ancMDA5/LGP2a and the MDA5-LGP2 duplication in early vertebrates, we observed a functional reversion back to ancRLR’s equal preference for blunt-ended and 5′ppp dsRNAs, even though the ‘RNA binding loop’ remained largely acidic (ancMDA5/LGP2b; Fig. 3). Combining information about protein-coding adaptation (Figs. 1 and 2) and molecular dynamics (Figs. 3, 4 and 5) suggested that a single H63S substitution may have contributed to the observed reversion in binding preference (see Fig. 2). To test this hypothesis, we measured changes in RNA preference caused by the single historical H63S substitution in the ancMDA5/LGP2a background (ancMDA5/LGP2a^H63S^). We found that this single substitution was sufficient to revert ancMDA5/LGP2a’s function to the ancestral-like pattern of equal binding to blunt-ended and 5′ppp dsRNA observed in ancMDA5/LGP2b (Fig. 7; Additional file 1: Figures S17 and S18). The H63S substitution increased affinity for 5′ppp dsRNA by ~20-fold (*p* < 0.02), while affinity for blunt-ended dsRNA remained unchanged (*p* > 0.06). RNA binding by ancMDA5/LGP2a^H63S^ was indistinguishable from that of ancMDA5/LGP2b (*p* > 0.28), suggesting that the single substitution was sufficient to recapitulate the observed functional shift from ancMDA5/LGP2a to ancMDA5/LGP2b.

Indroducing the H63S mutation into the ancMDA5/LGP2a background produced a modest increase in the average number of hydrogen bonds observed over molecular dynamics simulations between the protein and the 5′ppp dsRNA ligand as a whole (*p* = 0.04) as well as to the 5′ppp moiety in particular (*p* = 0.005; Additional file 1: Figure S26). No differences in overall protein-RNA hydrogen bonding were observed between the ancMDA5/LGP2a^H63S^ mutant and ancMDA5/LGP2b (*p* > 0.09), suggesting that the single H63S substitution is sufficient to shift the overall hydrogen bond network from the ancestral ancMDA5/LGP2a to the derived ancMDA5/LGP2b conformation.

We observed a single residue, R102, which dramatically increased its capacity to form hydrogen bonds with the RNA ligand in molecular dynamics simulations, from forming no observed hydrogen bonds in ancMDA5/LGP2a to forming favorable protein-RNA contacts in over 50 % of sampled timepoints in ancMDA5/LGP2b and the ancMDA5/LGP2a^H63S^ mutant (*p* < 0.02; Additional file 1: Figure S27). R102 is located at the end of β11 in ancMDA5/LGP2a, which is disordered in ancMDA5/LGP2b and the ancMDA5/LGP2a^H63S^ mutant and is far away from the H63S substitution (Fig. 7).

Examination of the central structures from molecular dynamics simulations revealed that the substitution of the small serine residue for the bulky histidine at position 63 introduced space into the region of the RNA-binding pocket between the ‘acidified RNA-binding loop’ and the Zn-binding finger, allowing the Zn finger to shift away from the RNA ligand. This allowed the RNA to move closer to the protein and rotate in the binding pocket to engage R102, which is flipped up in both ancMDA5/LGP2b and the ancMDA5/LGP2a^H63S^ mutant, compared to the ancestral MDA5/LGP2a (Fig. 7). As in our analysis of the ancRLR—ancMDA5/LGP2a transition, changes in a number of molecular-dynamics properties were consistent with a primary role for alteration of hydrogen bonding networks in explaining the differences in RNA preference between ancMDA5/LGP2a and ancMDA5/LGP2b (Additional file 1: Figures S28–S30).

Overall, these results reveal how the introduction of a single ‘destabilizing’ H63S substitution had a ‘ripple effect’ through the RD structure, changing the way existing residues far away from the substitution interact with the large RNA ligand. They also reveal that RLR RD evolution has exploited multiple, structurally-distinct evolutionary trajectories to implement and re-implement similar RNA-binding functions.

## Conclusions

Ancestral sequence resurrection (ASR) has allowed researchers to experimentally evaluate hypotheses about how the structures and functions of biological molecules evolve, deepening our understanding of important biological systems and informing molecular-evolutionary theory [5, 23, 37, 93]. To date, ASR studies have highlighted potentially strong epistasis among substitutions and historical contingency as major features of protein evolution [9, 10, 17, 94, 95] and have supported a model in which ancestral ‘promiscuity’ gives rise to increasing specialization as proteins ‘optimize’ specific functions over evolutionary time [39, 96, 97]. Major changes in protein function are generally observed to occur following gene duplication events [98–100] or speciation events [96, 101–103] and appear to be preserved over long periods of time. However, all such studies have examined molecular systems involved in conserved metabolic or developmental processes that we expect to be under relatively stable selection pressures. It is unclear whether the results of these studies generalize to fast-evolving immune receptors, which are expected to experience strong and frequent shifts in selection pressures.

Our study is the first to examine the structural mechanisms underlying long-term functional evolution in a fast-evolving primary immune receptor. In contrast to previous studies of slow-evolving proteins, we found no evidence for strong epistasis among substitutions, historical contingency or long-term ‘functional optimization.’ Rather, the repeated functional ‘flip flopping’ we observed was primarily caused by small numbers of substitutions that clustered in and around the ligand-binding pocket and exerted functional effects by reorganizing local structural dynamics. We also observed little evidence for strong structural constraints on RLR evolution, with similar functions evolving and re-evolving through novel structural mechanisms.

This study focused on two model RNA ligands that have been associated with viral infections [56, 74–79] and exhibit differential binding among RLRs [53, 80–83]. We observed changes in RLR binding to these model RNAs across evolutionary history, and the observed functional changes were associated with adaptive protein-coding substitutions. However, the functional shifts we observed could also be side-effects of adaptation primarily targeting other functions not examined in our study, including binding to other ligands or viral antagonization.

Unlike studies of ligand-binding proteins involved in more stable processes, the exact ligands recognized by modern human RLRs are still unclear [83, 104]. The dynamic nature of host-pathogen molecular interactions may result in potentially radical changes over time in the specific pathogen-associated molecules recognized by host immune receptors. Viral RNA sequences can change extremely rapidly, and although structural analyses and early functional studies have suggested that RLR-RNA interactions are not strongly affected by sequence variation [52, 84], changes in viral RNA sequences might impact RLR immune signaling under some circumstances [105–107]. Many viruses biochemically ‘hide’ their RNAs to avoid host detection [108–110], and viruses are known to antagonize various aspects of the RLR system [111–113]. Unfortunately, we know almost nothing about the ecologically important viruses that may have plagued ancient animals, so determining the precise native ligands that may have driven the evolution of RLRs is not possible. At this point, we must content ourselves with characterizing how interactions between RLRs and model biochemical ligands evolved. Although these studies can shed light on the structural and molecular-functional evolution of immune receptors, it is not possible to confidently infer specific properties of ancient viruses based on limited analyses of receptor-RNA interactions.

While our study has focused on the evolution of receptor-ligand binding affinity, factors other than ligand affinity affect how receptors ultimately function in immune signaling, including how receptors interact with themselves [114–117] with cofactors [48, 118, 119] and with signaling adaptors [47, 48, 120]. Making sense of the functional evolution of RLRs and other immune receptors will ultimately require considering the full breadth of molecular interactions determining a receptor’s cellular function.

While speculative, we feel our results may reflect general differences in the evolutionary dynamics of immune receptors, compared to those of proteins involved in more stable biological processes. Because the functional requirements of pathogen recognition are likely to change rapidly, immune receptors may exist in constant non-equilibrium, unable to optimize a specific functional repertoire. In contrast, proteins evolving toward a more stable evolutionary goal have more time to optimize specific functions. This general model would predict that immune receptors should exhibit less epistasis among substitutions overall, and extant receptors should be less optimally tuned to their functions, compared to slow-evolving counterparts. Determining the validity of this general model will require characterizing the molecular-functional evolution of RLRs across a broader range of ligands and over a larger slice of evolutionary history. Testing generalizability will also require mechanistic investigations of the evolution of other immune receptors. Characterizing a large number of receptor-ligand systems is expected to provide important clues about the specific aspects of pathogen-receptor interactions of primary importance in evolutionary history, advancing our understanding of host-pathogen interactions in particular as well as molecular-evolutionary theory in general.

## Methods

### Sequence data and gene family phylogeny

RIG-like receptor (RLR) protein sequences were retrieved from the NCBI Reference Sequence database [121] using DELTA-BLAST [122] with an e-value cutoff of 1.0e^−5^. We used the RNA recognition domains (RDs) from human RIG-I, MDA5, LGP2 and our previously reconstructed ancestral RLR RD [46] as BLAST queries, and merged the results into a single RLR dataset. We confirmed the presence of a DEAD/Helicase domain in each sequence by RPS-BLAST against the NCBI Conserved Domain Database [123], using an e-value cutoff of 0.01.

Sequences were aligned using PROBALIGN v1.4 (default parameters [124]) and MAFFT v7.158b (einsi parameters [125]). Initial maximum likelihood trees were built from each alignment using FastTree v2.1.7 (default parameters [126]) and further refined using the “BEST” topology search heuristic in PhyML v20140606 [127], with the best-fit evolutionary model inferred by AIC using ProtTest v3.4 [128]. We inferred SH-like aLRT clade support using PhyML [129]. Ancestral sequences were inferred by marginal maximum likelihood reconstruction using RAxML v8.0.25 [130], with insertions and deletions reconstructed by maximum likelihood from the presence-absence alignment matrix. Support for each ancestral state was assessed by calculating posterior probabilities [131], and ancestral sequences were compared to our previously reconstructed ancestral RLRs [46].

Protein-coding adaptation was assessed using the branch-sites model implemented in PAML v4.7 [132, 133], which uses a mixture distribution to model a combination of negatively-selected, neutral and positively-selected positions in the protein sequence [66]. For each branch on the phylogeny, we tested the hypothesis that some sites experienced adaptive protein-coding substitutions against the null hypothesis of neutral evolution using a likelihood ratio test. *P* values were calculated using the *χ* ^2^ distribution [66], and we used a Bonferroni correction for multiple testing.

### Structural modeling and molecular dynamics

We constructed structural models of RLR RDs using MODELLER v9.13 [134]. The structures of human RIG1 RD bound to blunt-ended double-stranded RNA (PDB ID: 3OG8 [51]) and 5′-triphosphate dsRNA (PDB ID: 3LRN [84]) were used as templates. We constructed 100 preliminary models of each RD-RNA and scored each model using the MODELLER objective function (molpdf), DOPE score and DOPEHR [135]. Each score was re-scaled to units of standard-deviation across the 100 models [136], and we selected the best structural model as that with the best average of re-scaled molpdf, DOPE and DOPEHR scores. Spatial and thermodynamic quality scores were assigned to each structural model using QMean [137], DFIRE [138] and Procheck [139]. Structural models of ancestral RLR helicase + pincer + RD domains bound to blunt dsRNA (PDB ID: 5F9F) and 5′ppp dsRNA (PDB ID: 5F9H) were constructed using the same approach [52].

Structural models were processed for electrostatic surface visualization using PROPKA v3.1 and PDB2PQR v1.7 to determine residue side-chain pKas, optimize the structure for favorable hydrogen bonding and calculate charge and radius parameters from amber electrostatic force fields [140, 141]. These calculations were performed using protein-RNA complexes to ensure proper side-chain orientation and protonation in the presence of ligand. For visualization, we estimated electrostatic surface potentials in the absence of RNA ligands using APBS v1.4.1 [142] and projected them onto the protein’s molecular surface.

For each RLR structure bound to each RNA type, we ran 4 replicate molecular dynamics simulations using GROMACS v4.6.5 [143]. Dynamics simulations of RLR RDs bound to RNA contained between 22,056 and 64,408 water molecules, 43–146 sodium and 44–120 chloride ions. Simulations of helicase + pincer + RD domains bound to RNA contained 32,906–35,871 waters, 67–83 sodium ions and 68–73 chloride ions. We used the amber99sb-ildn force field [144] and the tip3p water model. Initial dynamics topologies were generated for each RLR-RNA complex using the GROMACS pdb2gmx algorithm with default parameters. Topologies were relaxed into simulated solvent at pH = 7 using a 50,000-step steepest-descent energy minimization. The system was then brought to 300K using a 50-ps dynamics simulation under positional restraints, followed by pressure stabilization for an additional 50 ps. Unconstrained molecular dynamics were run for 11 ns using a 0.002-ps integration time step, with the system sampled every 5 ps. Simulations were run using Particle-Mesh Ewald electrostatics with cubic interpolation and grid spacing of 0.12 nm. Van der Waals forces were calculated using a cutoff of 1.0 nm. We used Nose-Hoover temperature coupling, with protein, RNA and solvent systems coupled separately and the period of temperature fluctuations set to 0.1 ps. Pressure coupling was applied using the Parrinello-Rahman approach, with a fluctuation period of 2.0 ps. Non-bonded cutoffs were treated using buffered Verlet lists. We discarded the first 1 ns of each simulation.

From each dynamics simulation, we inferred the central structure by calculating pairwise root mean square deviations (RMSDs) between every pair of simulation samples and identifying the sampled structure most equidistant to the others, using the g_cluster function in GROMACS. We measured the root mean square fluctuation (RMSF) of each residue’s side-chain and backbone over the dynamics simulation. We additionally calculated a number of biochemical properties from each sample taken from each molecular dynamics simulation. Secondary structure was calculated using DSSP v2.2.1 [145], and we report the proportion of samples from which each residue was a member of a helix, strand or loop. We estimated a consensus secondary structure over the course of each simulation by assigning each residue to helix or strand if it was assigned to that secondary structure by DSSP in >50 % of samples; we required at least 3 consecutive residues to infer consensus helices and strands. We inferred hydrogen bonds using a radius cutoff of 0.3 nm and an angle cutoff of 20 ° and report, for each residue, the proportion of simulation samples from which that residue forms a hydrogen bond with the RNA molecule or the 5′ppp moiety. We calculated the minimum distance between each residue and the RNA molecule over the course of each dynamics simulation. We considered a residue as potentially contributing to RNA contact if its average minimum distance across the entire dynamics simulation was significantly <4 angstroms.

Significance of differences in each biochemical property inferred by structural dynamics was assessed using the 2-tailed, 2-sample independent *t*-test, assuming unequal variances [146]. We corrected for multiple testing using a false discovery rate correction [147].

### Molecular binding kinetics

We generated GC-rich 29-base-pair RNA molecules in vitro using T7 RNA reverse transcriptase and synthetic dsDNA as template (3′-GAAAGAGGUGCGGAAAGAGGUAGAGGAGG-5′). Complementary purified single-stranded RNAs were annealed to produce double-stranded RNA by combining at 1:1 ratio, heating to 95 °C for 5 min and then cooling to 25 °C. Blunt-ended dsRNA was produced from 5′-triphosphate RNA by exposure to alkaline phosphatase or synthesized *de novo* by IDT (Iowa, USA). The 3′ end of one RNA strand was biotinylated to facilitate kinetics assays using the Pierce™ 3′ End RNA Biotinylation Kit (Thermo).

RLR RDs were expressed in *E. coli* Rosetta™ 2(DE3)pLysS cells using pET-22b(+) constructs, which were verified by Sanger sequencing. Proteins were purified by His-affinity purification and visualized by SDS-page stained with 1 % coomassie. Protein concentrations were measured using a linear-transformed Bradford assay [148].

We measured RD-RNA binding using a label-free in vitro kinetics assay at pH = 7 [149]. Biotinylated RNA molecules were bound to a series of 8 streptavidin probes for 15 min, until saturation was observed. Probes were washed and then exposed to 25 μg/ml biocytin to bind any remaining free streptavidin. The probes were then exposed in parallel to RDs at various concentrations in 1 × Kinetics Buffer (ForteBio) for 30 min, followed by dissociation in Kinetics Buffer for an additional 30 min. Molecular binding at each concentration over time was measured as the change in laser wavelength when reflected through the probe in solution, sampled every 3 ms.

For each replicate experiment, we estimated the RD concentration at which ½-maximal steady-state RNA binding was achieved (Kd) by fitting a one-site binding curve to the steady-state laser wavelengths measured across RD concentrations at saturation (60 min), using nonlinear regression. We additionally fit 1-site association/dissociation curves to the full time-course data in order to estimate the initial rates of RNA binding across RD concentrations and used these rates to calculate the RD concentration at which the ½-maximal RNA-binding rate was achieved (Km). Kds and Kms were negative-log transformed to facilitate visualization, and standard errors across 3 experimental replicates were calculated. We calculated the statistical significance of differences between Kds and Kms using the 2-tailed, 2-sample independent *t*-test, assuming unequal variances [146].

## Additional file

Additional file 1: Supplementary figures and tables. (PDF 9731 kb)

## Abbreviations

5′ppp: Five-prime triphosphate
ASR: Ancestral sequence resurrection
dsRNA: Double-stranded RNA
RD: RNA recognition domain
RLR: RIG-like receptor
