# Supplementary material for: Resurrecting ancestral structural dynamics of an antiviral immune receptor: adaptive binding pocket reorganization repeatedly shifts RNA preference
Source: BMC Evol Biol. 2016 Nov 8;16:241. doi: 10.1186/s12862-016-0818-6 (PMC5101713; doi:10.1186/s12862-016-0818-6)
Supplement: Additional file 1: — Supplementary figures and tables. (PDF 9731 kb) [file 12862_2016_818_MOESM1_ESM.pdf]

Additional File 1

Supplementary Figures

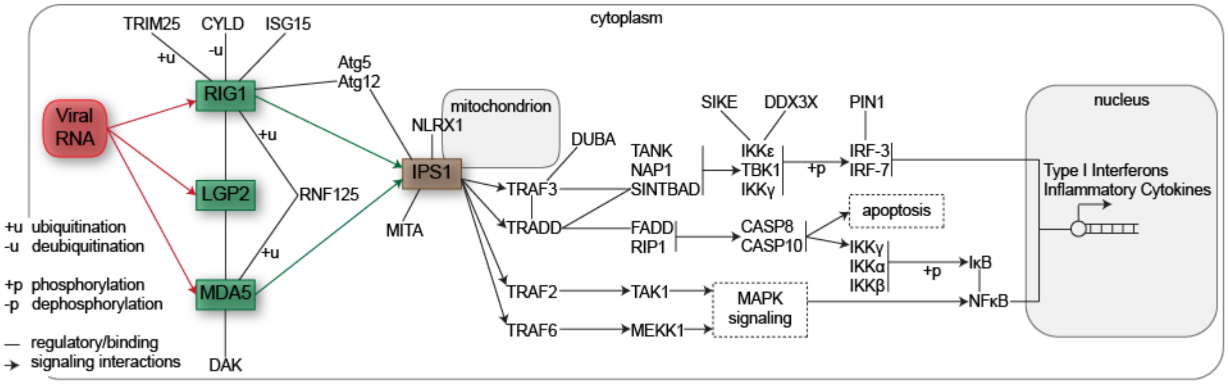

Figure S1. RIG-like receptors (RLRs) bind viral RNA and initiate immune-signaling cascades through direct interactions with IPS1. We show a simplified diagram of the main protein-protein interactions involved in immune signaling by RLRs, taken largely from the human "RIG-I-like receptor signaling pathway" in KEGG [1] and review of relevant literature. The RLRs are shown in green, with IPS1 in brown and viral RNA ligands in red. Primary signaling interactions are displayed by arrows, with plain lines indicating regulatory or physical interactions. Cellular compartments are outlined and shaded. Collapsed sub-networks are displayed using dotted outlines. Important (de)ubiquitination and (de)phosphorylation reactions are indicated. Although all three RLRs bind viral RNA, only RIG-I and MDA5 interact directly with the signal-transducing protein, IPS1 [2]. LGP2 appears to play regulatory roles during some viral infections, although the precise mechanisms are unknown [3-5]. Cellular results of RLR-initiated immune signaling include activation of type I interferons and inflammatory cytokines as well as initiation of apoptosis and direct activation of the inflammasome.

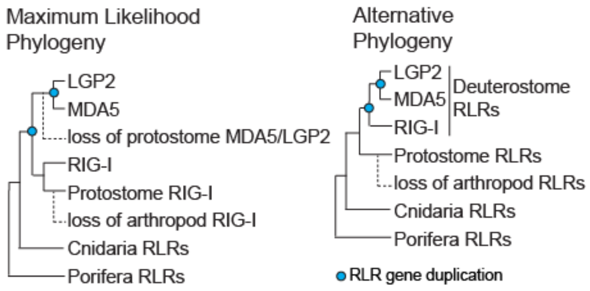

Figure S2. Statistical topology tests reject deuterostome-specific expansion of RIG-like receptors (RLRs). We compared the statistical support for the maximum likelihood phylogeny (left) to the alternative tree (right), in which all RLR gene duplications occurred after the protostome-deuterostome split. The AU test rejected the alternative phylogeny, assuming both PROBALIGN (p=0.023) and MAFFT (p=0.018) alignments. Dotted lines indicate inferred gene loss events, given each phylogeny.

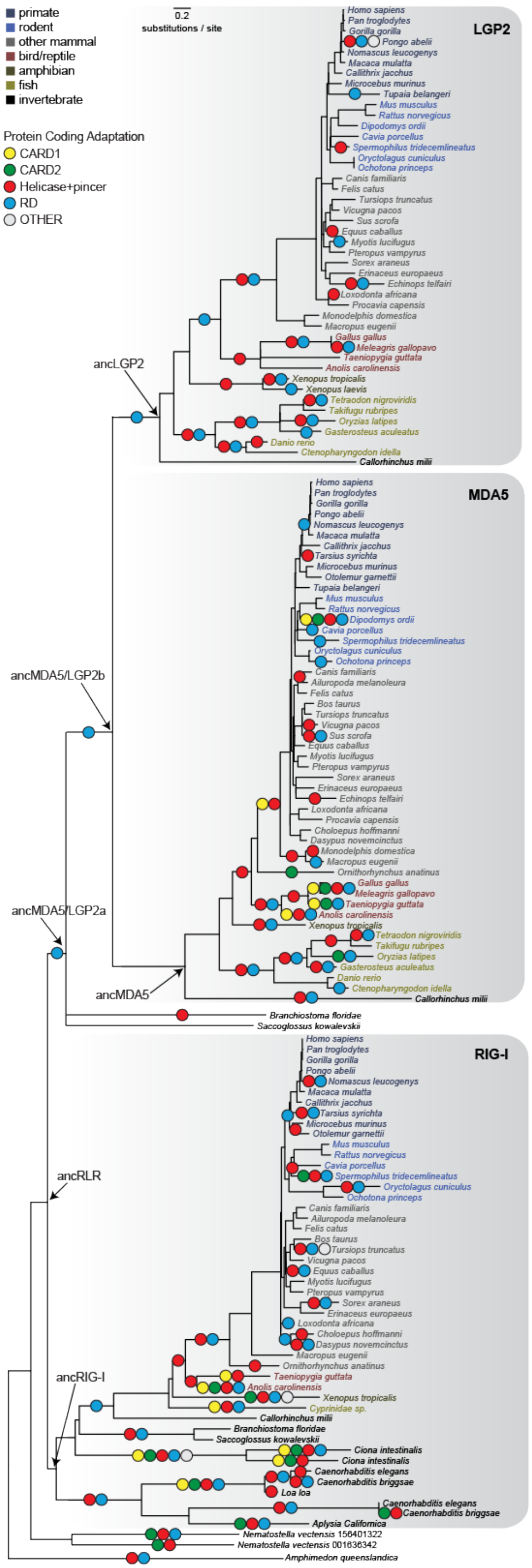

primate
rodent
other mammal
bird/reptile
amphibian
fish
invertebrate
0.2
substitutions / site
Protein Coding Adaptation
CARD1
CARD2
Helicase+pincer
RD
OTHER
LGP2
MDA5
RIG-I
ancLGP2
ancMDA5/LGP2b
ancMDA5/LGP2a
ancMDA5
ancRLR
ancRIG-I
Homo sapiens
Pan troglodytes
Gorilla gorilla
Pongo abelii
Nomascus leucogenys
Macaca mulatta
Callithrix jacchus
Microcebus murinus
Tupaia belangeri
Mus musculus
Rattus norvegicus
Dipodomys ordii
Cavia porcellus
Spermophilus tridecemlineatus
Oryctolagus cuniculus
Ochotona princeps
Canis familiaris
Felis catus
Tursiops truncatus
Vicugna pacos
Sus scrofa
Equus caballus
Myotis lucifugus
Pteropus vampyrus
Sorex araneus
Erinaceus europaeus
Echinops telfairi
Loxodonta africana
Procavia capensis
Monodelphis domestica
Macropus eugenii
Gallus gallus
Meleagris gallopavo
Taeniopygia guttata
Anolis carolinensis
Xenopus tropicalis
Xenopus laevis
Tetraodon nigroviridis
Takifugu rubripes
Oryzias latipes
Gasterosteus aculeatus
Danio rerio
Ctenopharyngodon idella
Callorhinchus milii
Homo sapiens
Pan troglodytes
Gorilla gorilla
Pongo abelii
Nomascus leucogenys
Macaca mulatta
Callithrix jacchus
Tarsius syrichta
Microcebus murinus
Otolemur garnettii
Tupaia belangeri
Mus musculus
Rattus norvegicus
Dipodomys ordii
Cavia porcellus
Spermophilus tridecemlineatus
Oryctolagus cuniculus
Ochotona princeps
Canis familiaris
Ailuropoda melanoleura
Felis catus
Bos taurus
Tursiops truncatus
Vicugna pacos
Sus scrofa
Equus caballus
Myotis lucifugus
Pteropus vampyrus
Sorex araneus
Erinaceus europaeus
Echinops telfairi
Loxodonta africana
Procavia capensis
Choloepus hoffmanni
Dasypus novemcinctus
Monodelphis domestica
Macropus eugenii
Ornithorhynchus anatinus
Gallus gallus
Meleagris gallopavo
Taeniopygia guttata
Anolis carolinensis
Xenopus tropicalis
Tetraodon nigroviridis
Takifugu rubripes
Oryzias latipes
Gasterosteus aculeatus
Danio rerio
Ctenopharyngodon idella
Callorhinchus milii
Branchiostoma floridae
Saccoglossus kowalevskii
Homo sapiens
Pan troglodytes
Gorilla gorilla
Pongo abelii
Nomascus leucogenys
Macaca mulatta
Callithrix jacchus
Tarsius syrichta
Microcebus murinus
Otolemur garnettii
Mus musculus
Rattus norvegicus
Cavia porcellus
Spermophilus tridecemlineatus
Oryctolagus cuniculus
Ochotona princeps
Canis familiaris
Ailuropoda melanoleura
Felis catus
Bos taurus
Tursiops truncatus
Vicugna pacos
Equus caballus
Myotis lucifugus
Pteropus vampyrus
Sorex araneus
Erinaceus europaeus
Loxodonta africana
Choloepus hoffmanni
Dasypus novemcinctus
Macropus eugenii
Ornithorhynchus anatinus
Taeniopygia guttata
Anolis carolinensis
Xenopus tropicalis
Cyprinidae sp.
Callorhinchus milii
Branchiostoma floridae
Saccoglossus kowalevskii
Ciona intestinalis
Ciona intestinalis
Caenorhabditis elegans
Caenorhabditis briggsae
Loa loa
Caenorhabditis elegans
Caenorhabditis briggsae
Aplysia Californica
Nematostella vectensis 156401322
Nematostella vectensis 001636342
Amphimedon queenslandica

Figure S3. Protein-coding adaptation affected only the RD early in RLR evolutionary history. After reducing the number of vertebrate sequences by including only representative species, we tested each branch on the RLR phylogeny for protein-coding adaptation targeting specific functional domains (CARD1, CARD2, Helicase+pincer, RD or other regions of the protein sequence) using a branch-sites test (see Methods). Colored circles indicate significant support for protein-coding adaptation specific to the indicated domain (p<0.05 after correction for multiple tests).

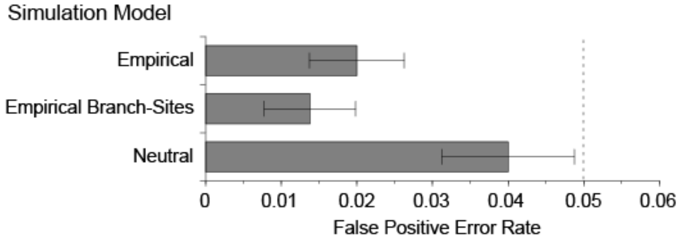

Figure S4. Simulations suggest low expected false-positive error rates for branch-sites tests of positive selection on the RLR phylogeny. We used PAML v4.7 to simulate 100 replicates of codon sequence data along the maximum-likelihood RLR phylogeny using three simulation models. The Empirical model estimated all simulation parameters from our empirical sequence data assuming a 'sites' model, in which an inferred proportion of sites evolve with nonsynonymous/synonymous rate ratio, ω<1, and the remaining sites evolve with ω=1. The Empirical Branch-Sites model is the same as the Empirical model, except all sites are released from selective constraint (ω=1) on the specific branch being tested. The Neutral model allows all sites to evolve neutrally (ω=1) on all branches. All other model parameters were estimated by maximum likelihood. We analyzed each replicate data set using the same procedure used to test our empirical data for protein-coding adaptation on specific branches (see Methods). We plot the mean and standard error in false-positive error rate over the 100 data sets simulated using each model. Dotted line indicates false-positive rate of 0.05.

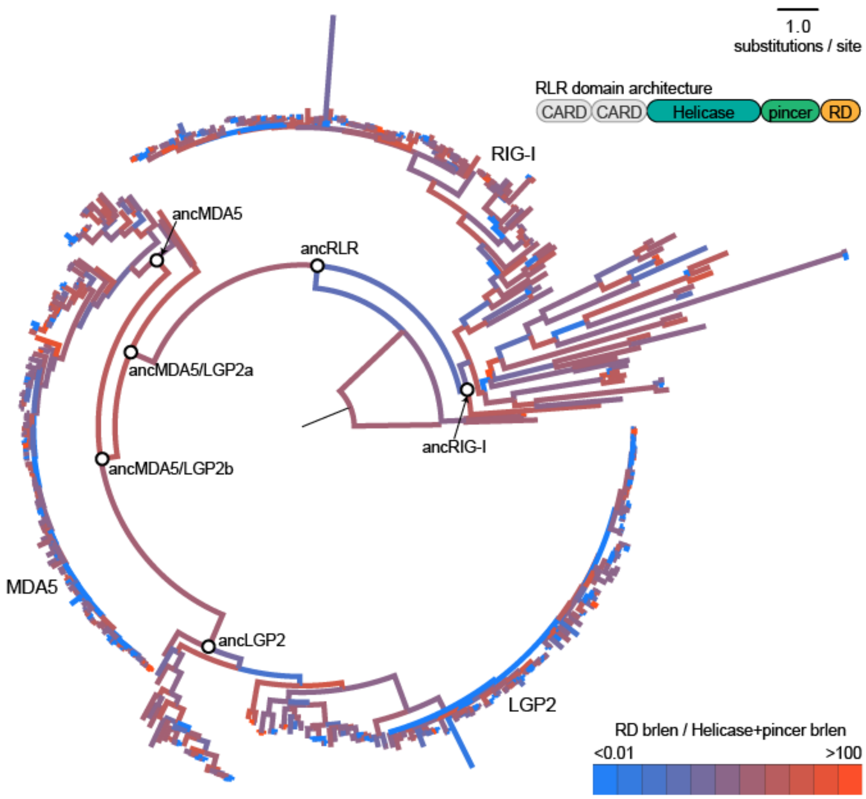

Figure S5. RLR RD evolved more rapidly than combined Helicase+pincer domains along the early branches of the RLR phylogeny between ancRLR and the MDA5/LGP2 lineages. We reconstructed the RLR gene phylogeny by maximum likelihood using two different alignments of all available RLR protein sequences (see Methods, Fig. 1). Branch lengths are scaled to the average number of substitutions/site across PROBALIGN and MAFFT alignments. We colored each branch according to the ratio of the length estimated using only the RD portion of the sequence alignment to the length estimated using the Helicase+pincer portion of the alignment. Blue indicates branches on which the Helicase+pincer evolved more rapidly than the RD, whereas red indicates branches on which the RD evolved faster than the Helicase+pincer.

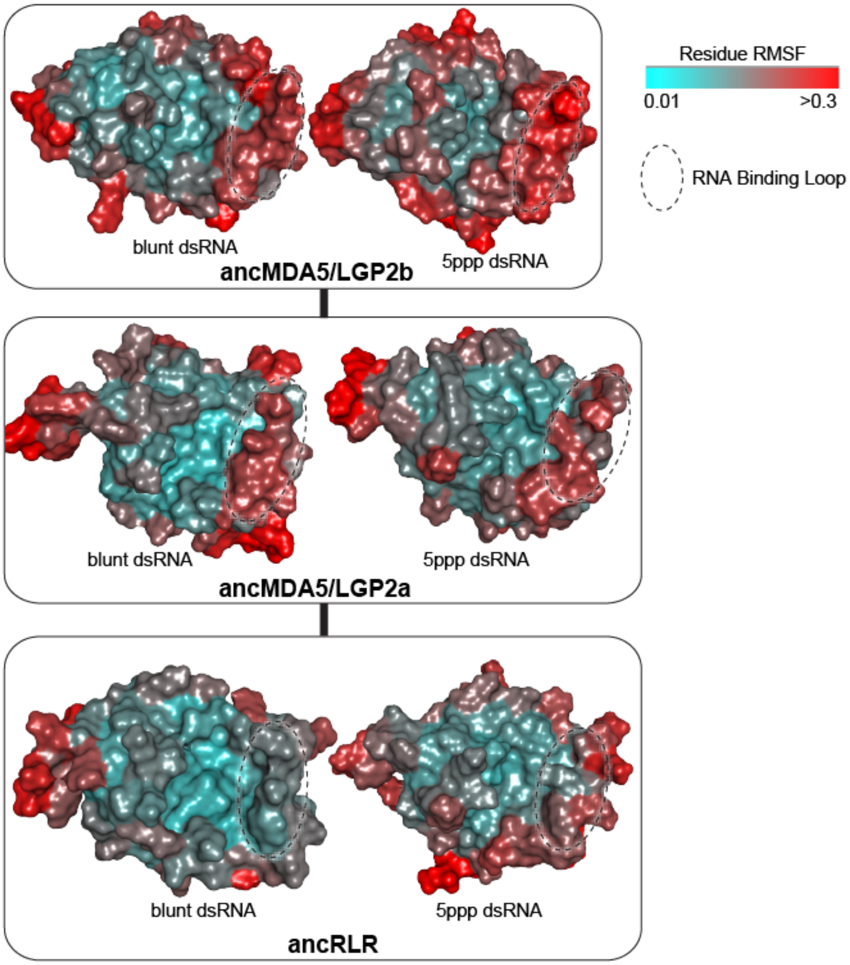

Figure S6. Fluctuation of the RNA-binding loop increased in ancMDA5/LGP2a and ancMDA5/LGP2b, compared to ancRLR. We plot the root mean squared fluctuation (RMSF) of each residue on the molecular surface of ancestral RLR RNA-recognition domains (RD), averaged over replicate molecular dynamics simulations of the combined helicase+pincer+RD bound to each type of RNA (see Methods). Higher values of RMSF indicate that the residue moved more over the course of the dynamics simulations. Dotted oval indicates location of the RNA-binding loop on each structure. See Figs. 1,2 for locations of each ancestral RLR on the phylogeny and ancestral RD sequences, respectively.

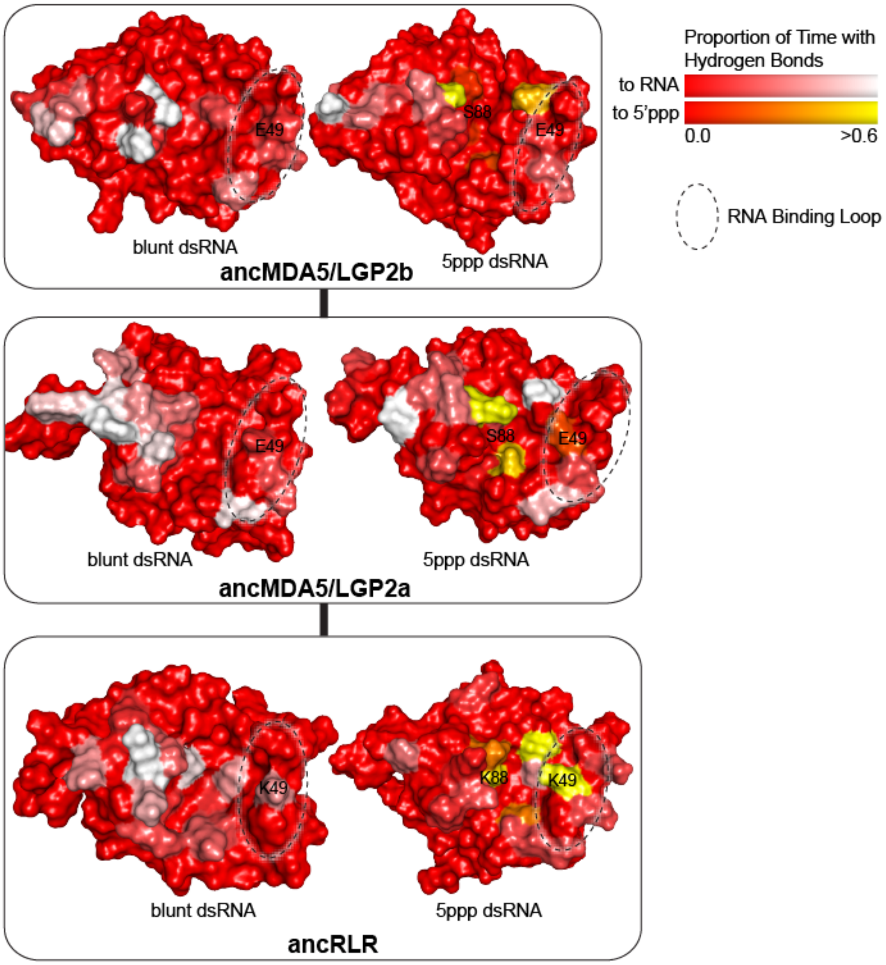

Figure S7. The RNA-binding loop loses hydrogen bonding to RNA ligands in the MDA5/LGP2 lineage after the first RLR gene duplication. We plot the proportion of molecular dynamics time points during which each residue was observed to form hydrogen bonds with the RNA ligand (red-white gradient) or the 5'ppp moiety in particular (red-yellow gradient), averaged over replicate simulations of ancestral RLR helicase+pincer+RD domains (see Methods). The RNA-binding loop and specific residues exhibiting reduced hydrogen bonding to 5'ppp dsRNA in ancMDA5/LGP2a-b are indicated.

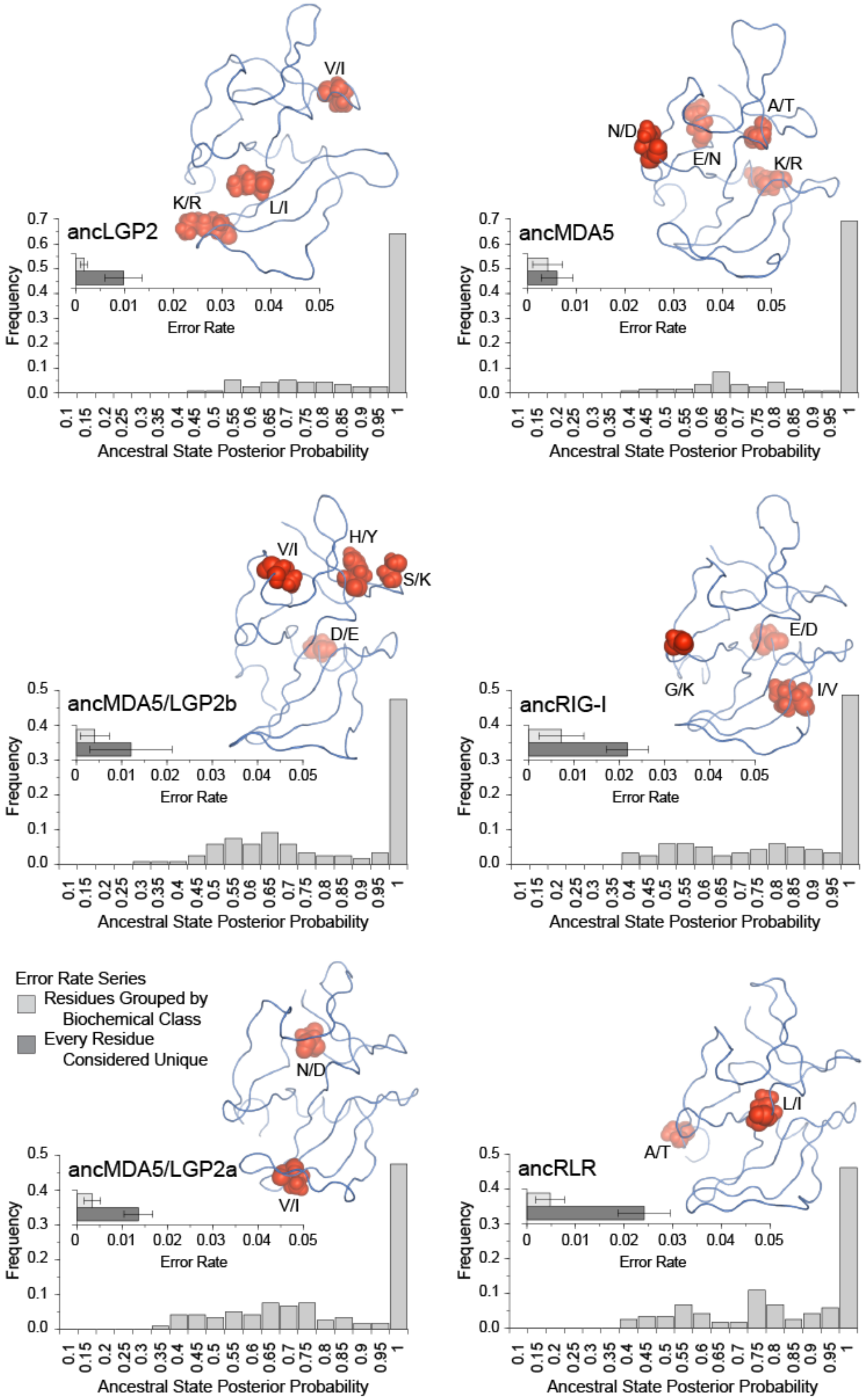

Figure S8. Ancestral RLR RD protein sequences were reconstructed with high confidence and low ambiguity. We reconstructed the ancestral sequences of key nodes on the RLR phylogeny (see Figs. 1,2). For each sequence, we plot the frequency with which individual residues were reconstructed with posterior probability ranging from 0.0 to 1.0, binned every 0.05. Inset into each graph, we simulated protein sequence data along the maximum-likelihood phylogeny using the best-fit evolutionary model and

plot the proportion of ancestral residues incorrectly inferred at each node. Dark series indicate error rates when each residue is considered unique, whereas light series indicate error rates when residues with similar biochemical properties are treated as equivalent; bars indicate standard errors. We show the locations on the RD structural models of residues having alternative ancestral reconstructions with posterior probability > 0.3.

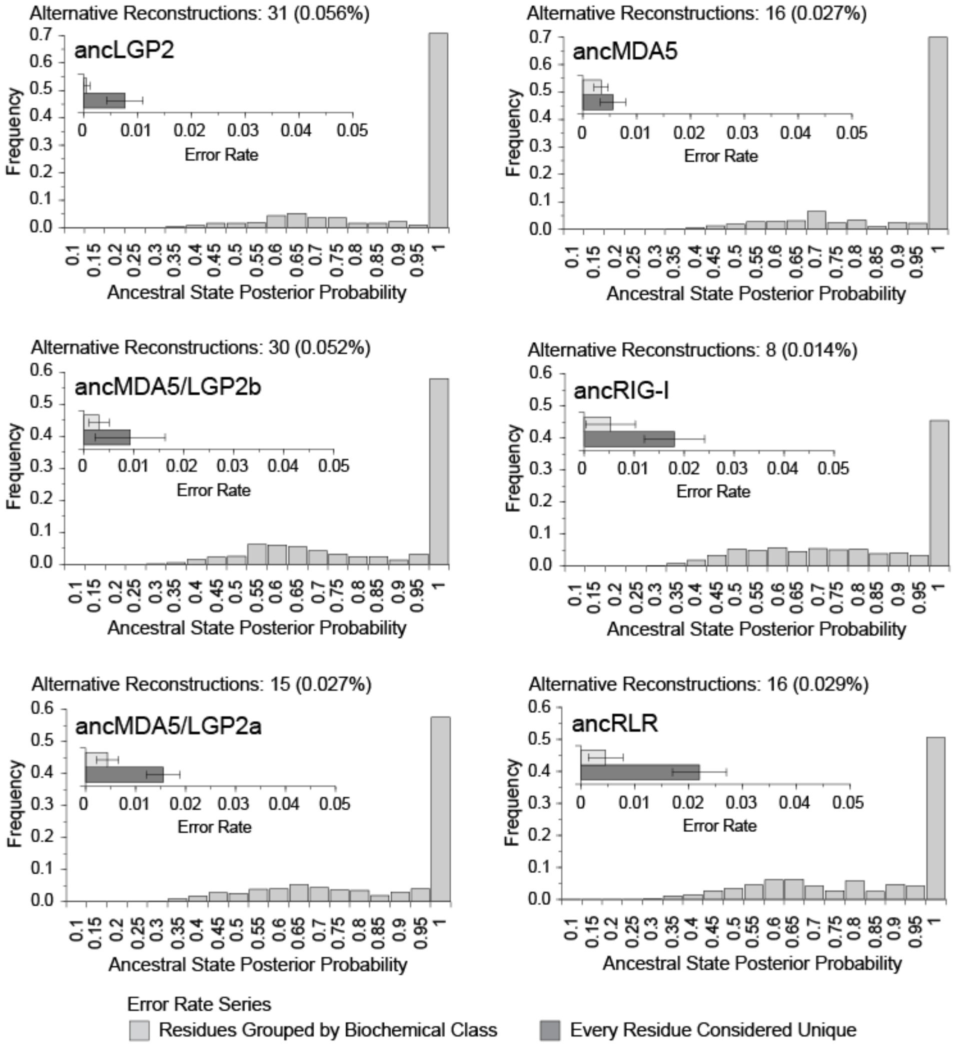

Figure S9. Ancestral RLR helicase+pincer protein sequences were reconstructed with high confidence and low ambiguity. We reconstructed the ancestral helicase+pincer sequences of key nodes on the RLR phylogeny (see Fig. 1). For each sequence, we plot the frequency with which individual residues were reconstructed with posterior probability ranging from 0.0 to 1.0, binned every 0.05. Inset into each graph, we simulated protein sequence data along the maximum-likelihood phylogeny using the best-fit evolutionary model and plot the proportion of ancestral residues incorrectly inferred at each node. Dark series indicate error rates when each residue is considered unique, whereas light series indicate error rates when residues with similar biochemical properties are treated as equivalent; bars indicate standard errors. We indicate the number (and percentage) of helicase+pincer residues having alternative ancestral reconstructions with posterior probability > 0.3.

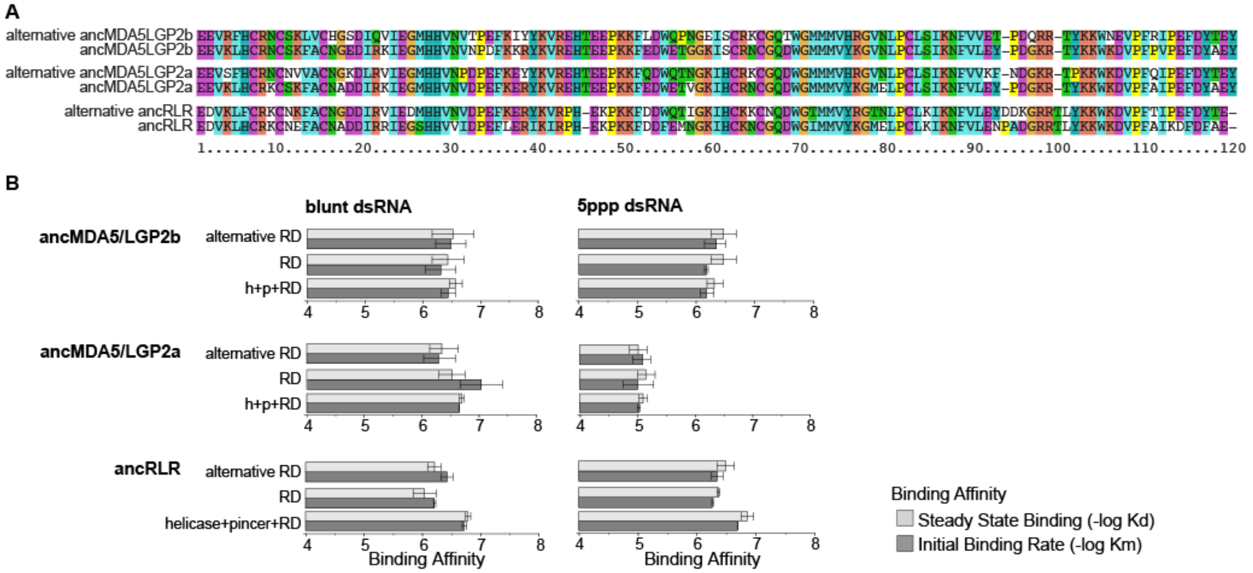

Figure S10. Ancestral sequence reconstructions were similar across different alignments and exhibited similar RNA affinities. We reconstructed ancestral RD sequences at key nodes on the RLR phylogeny (see Fig. 1) using the MAFFT protein sequence alignment (indicated as “alternative”). **A.** We compare ancestral RD protein sequences between the alternative MAFFT reconstruction and the original reconstruction using the PROBALIGN alignment. **B.** We measured steady-state (Kd) and initial (Km) RNA binding affinities for each ancestral RLR to blunt and 5’ppp dsRNA (see Methods). We plot –log-transformed binding affinities, with longer bars indicating higher affinity. Standard errors over three replicates are indicated. We compare RNA binding affinities measured for the PROBALIGN-reconstructed RDs and combined helicase+pincer+RD domains to the alternative MAFFT RD reconstructions.

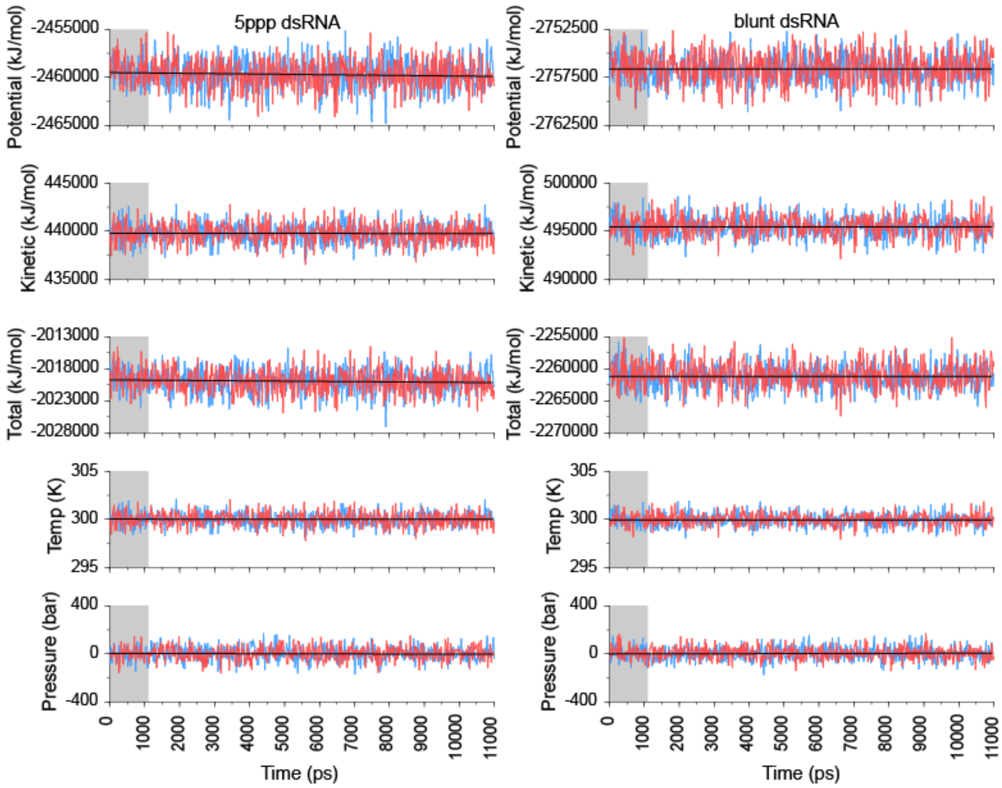

Figure S11 Part 1. ancLGP2.

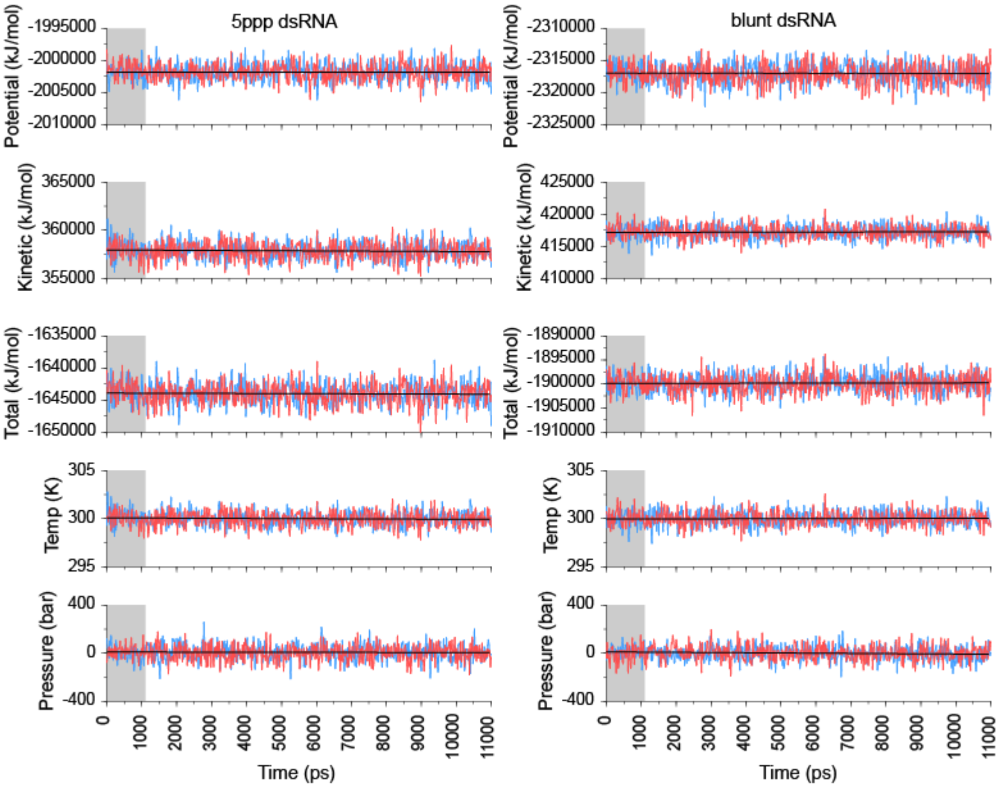

Figure S11 Part 2. ancMDA5.

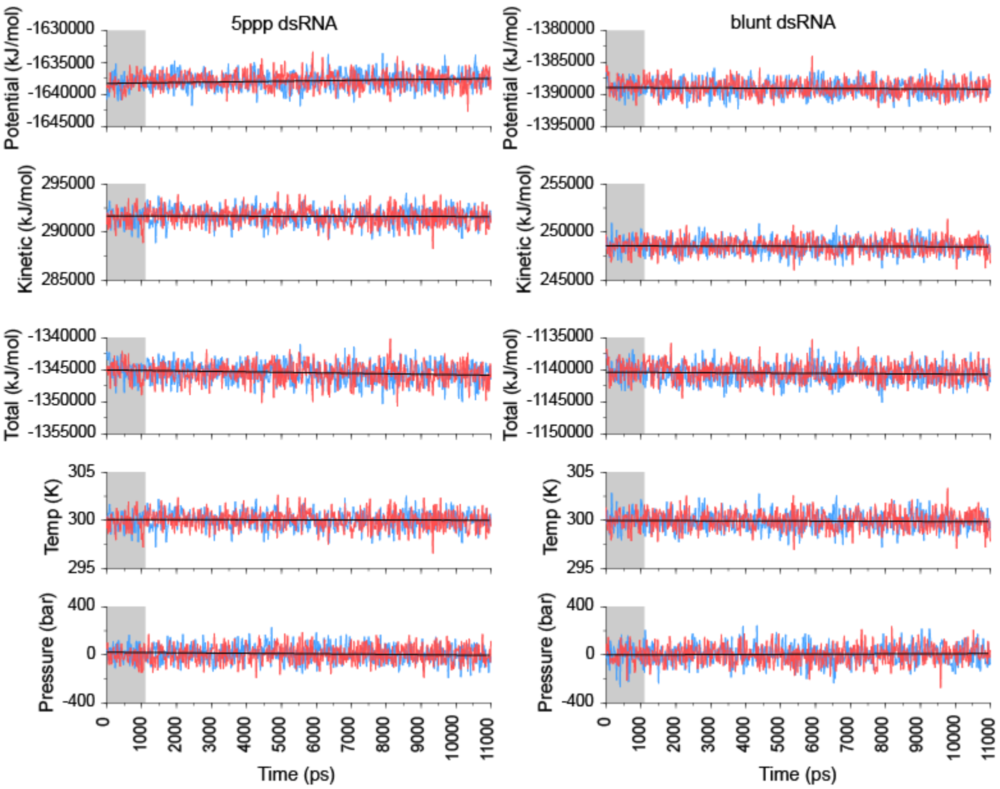

Figure S11 Part 3. ancMDA5/LGP2b.

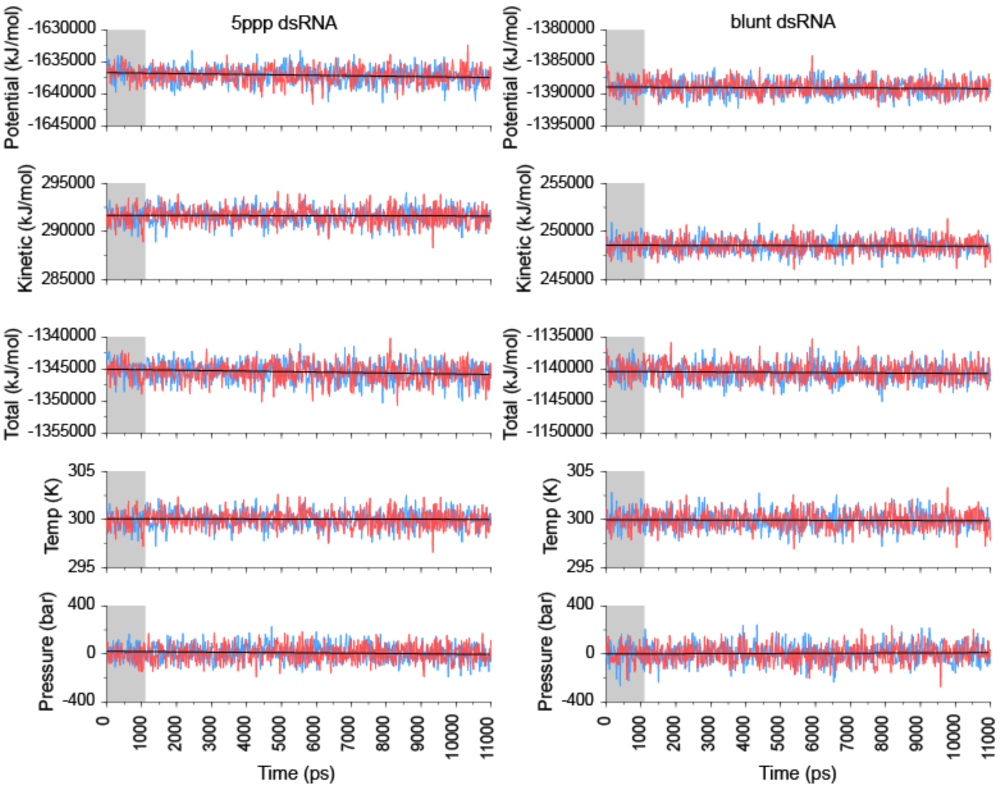

Figure S11 Part 4. ancMDA5/LGP2a.

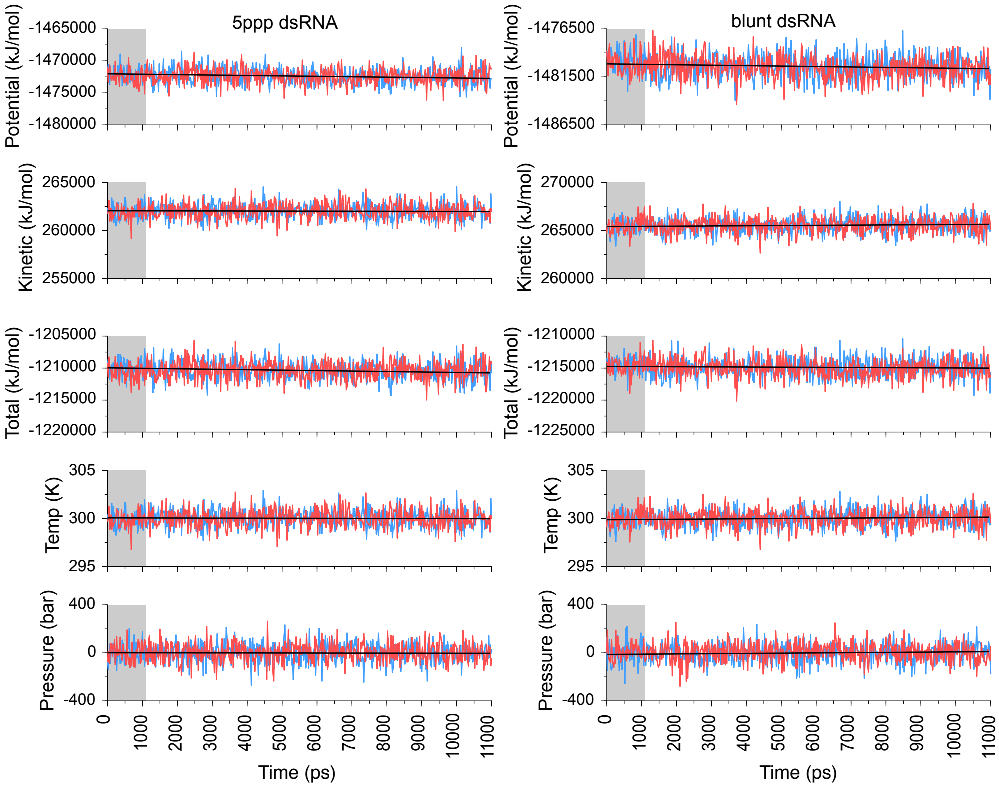

Figure S11 Part 5. ancRIG-I.

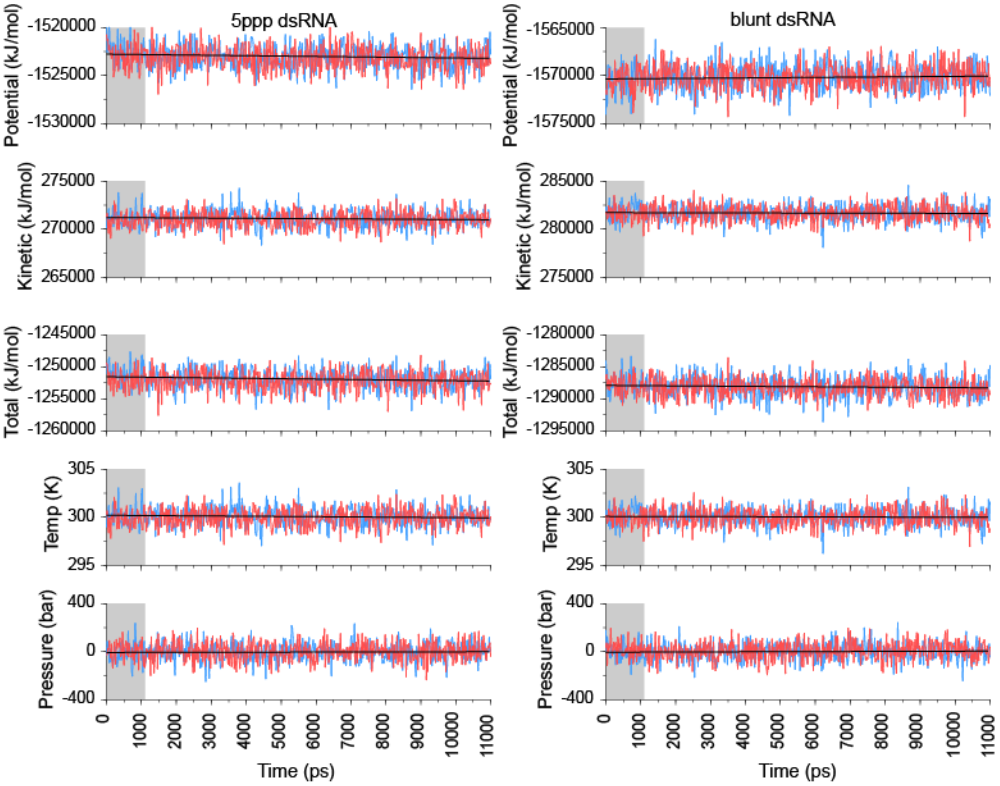

Figure S11 Part 6. ancRLR.

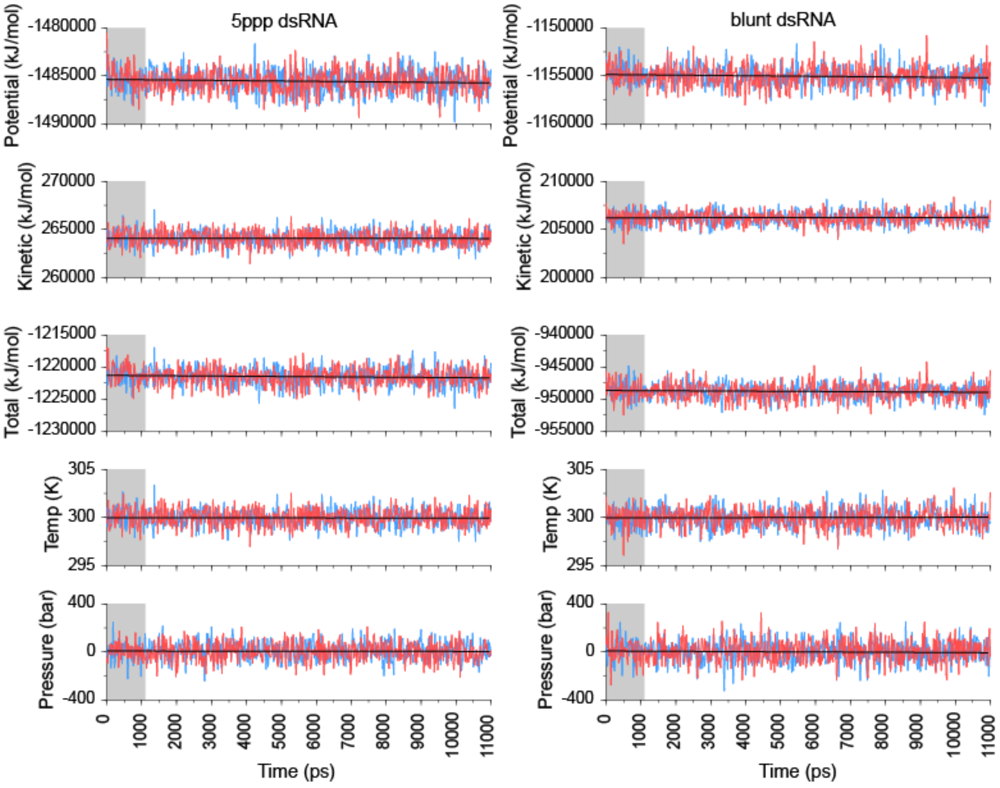

Figure S11 Part 7. ancMDA5/LGP2a^{H63S}.

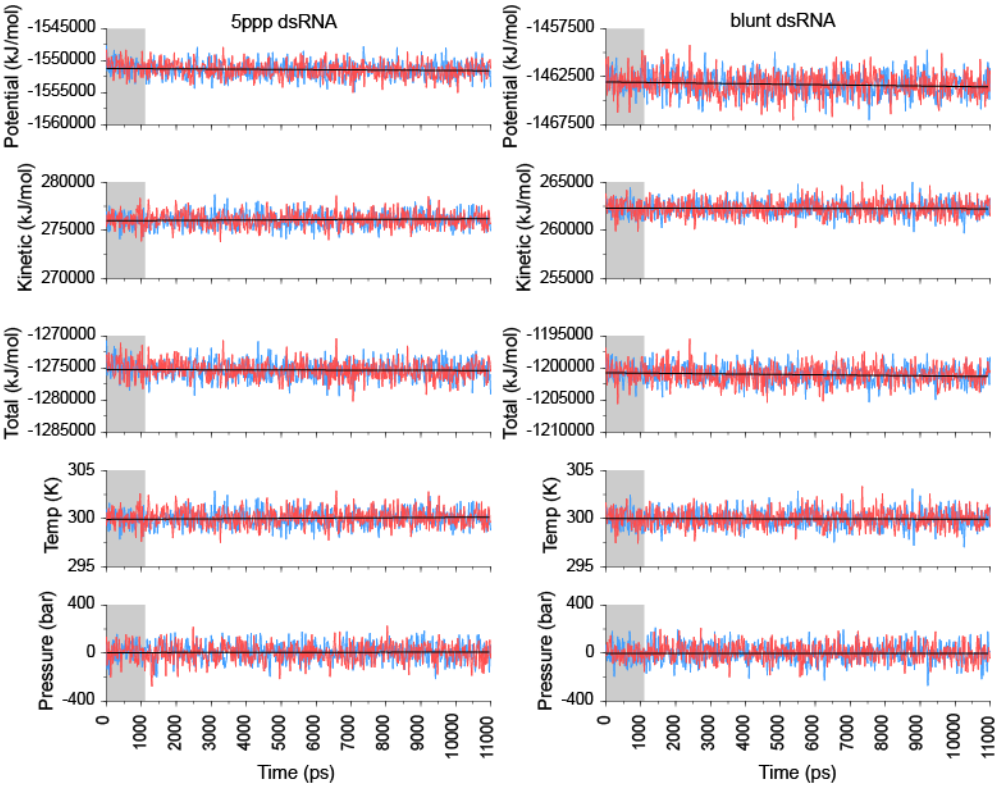

Figure S11 Part 8. ancMDA5/LGP2a$^{TEE47\Delta EK}$.

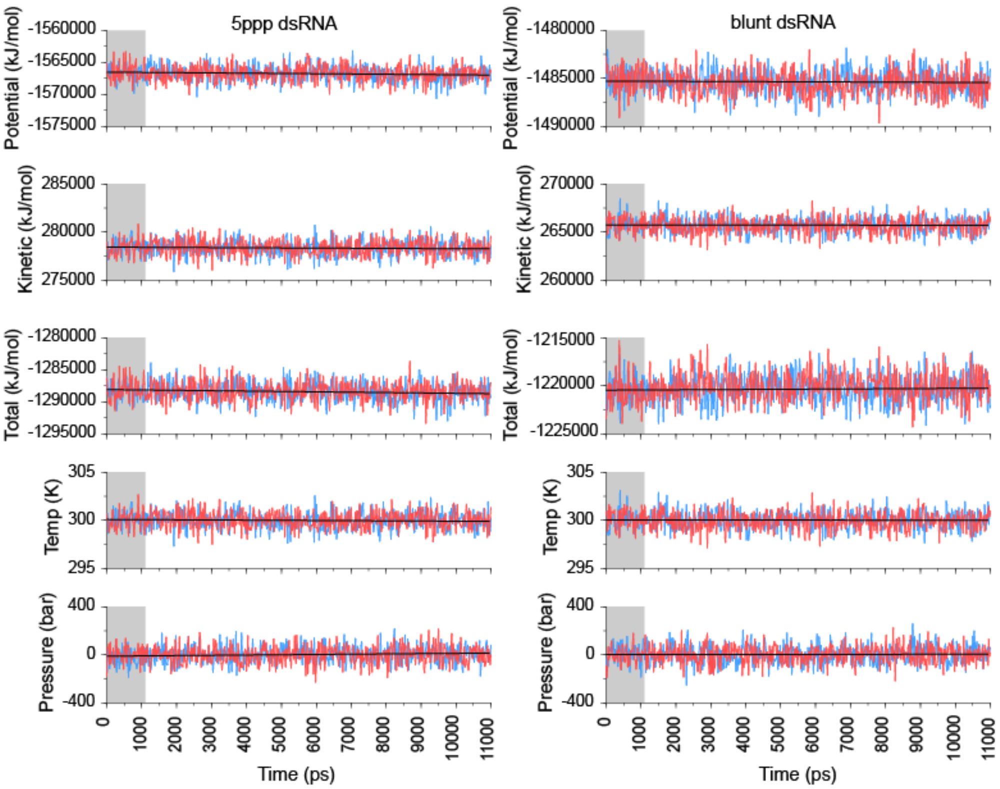

Figure S11 Part 9. ancRLR$^{\Delta EK47TEE,K88S}$.

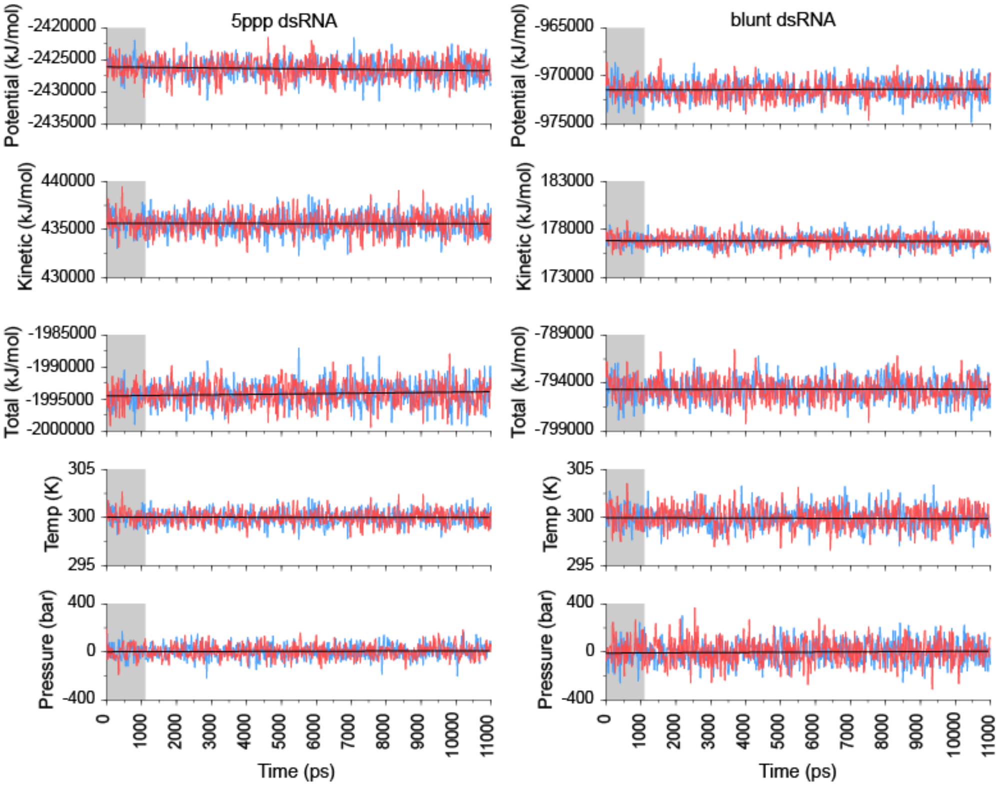

Figure S11 Part 10. Human LGP2.

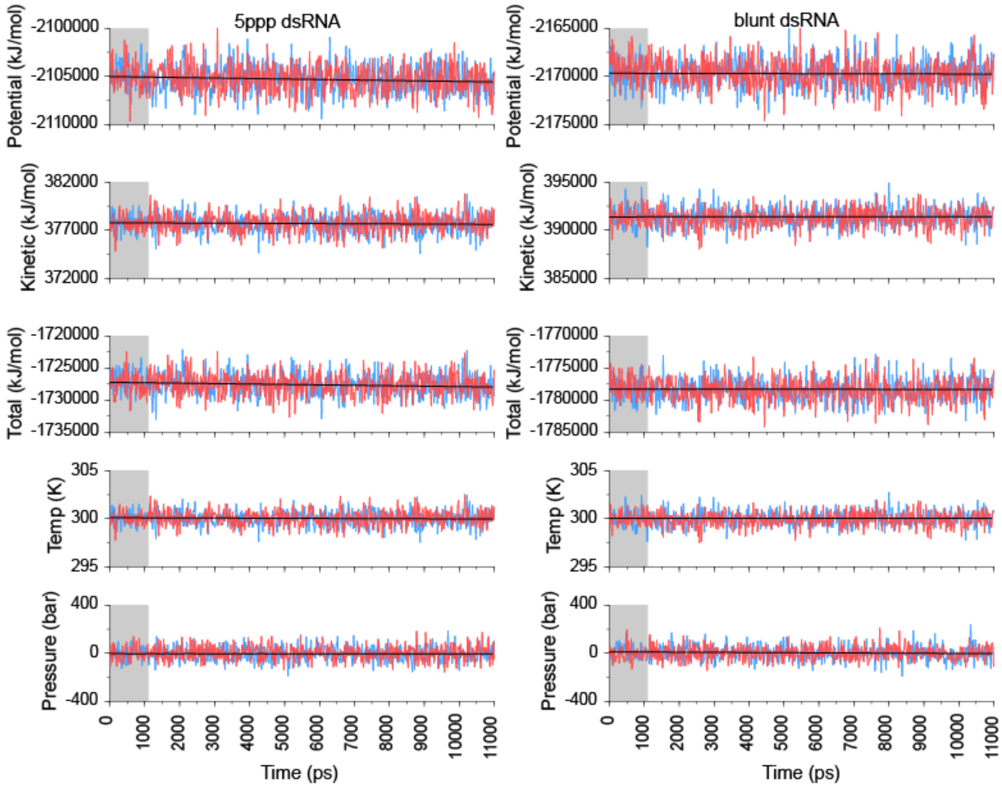

Figure S11 Part 11. Human MDA5.

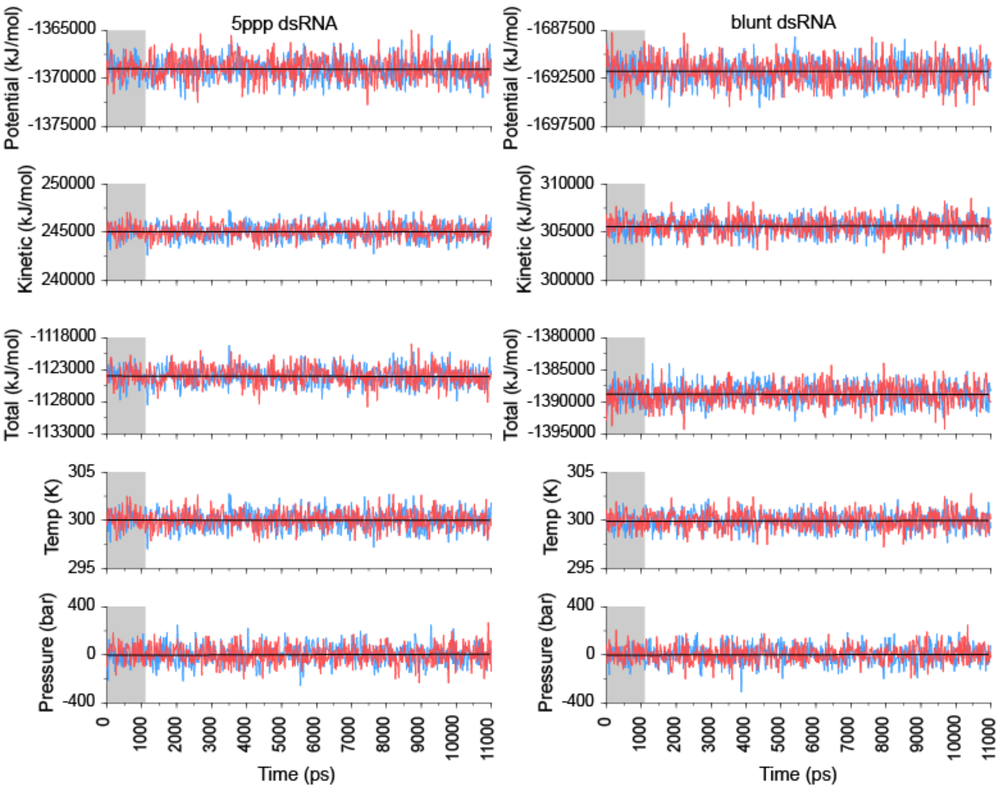

Figure S11 Part 12. Human RIG-I.

Figure S11. Energy parameters were well-behaved across molecular dynamics simulations of human and ancestral RLR RDs bound to 5'ppp and blunt-ended dsRNA. We plot potential, kinetic and total energy, as well as temperature and pressure, across the time course of each molecular dynamics simulation. Red and blue series indicate independent simulations of two RD proteins bound to either end of a double-stranded RNA molecule. We verified that characteristics of the RD-RNA complexes were less correlated between RDs in the same simulation than they were across simulations and considered each RD-RNA complex as an independent replicate for further analysis. Gray box indicates the first nanosecond of simulation, which we excluded from downstream analyses.

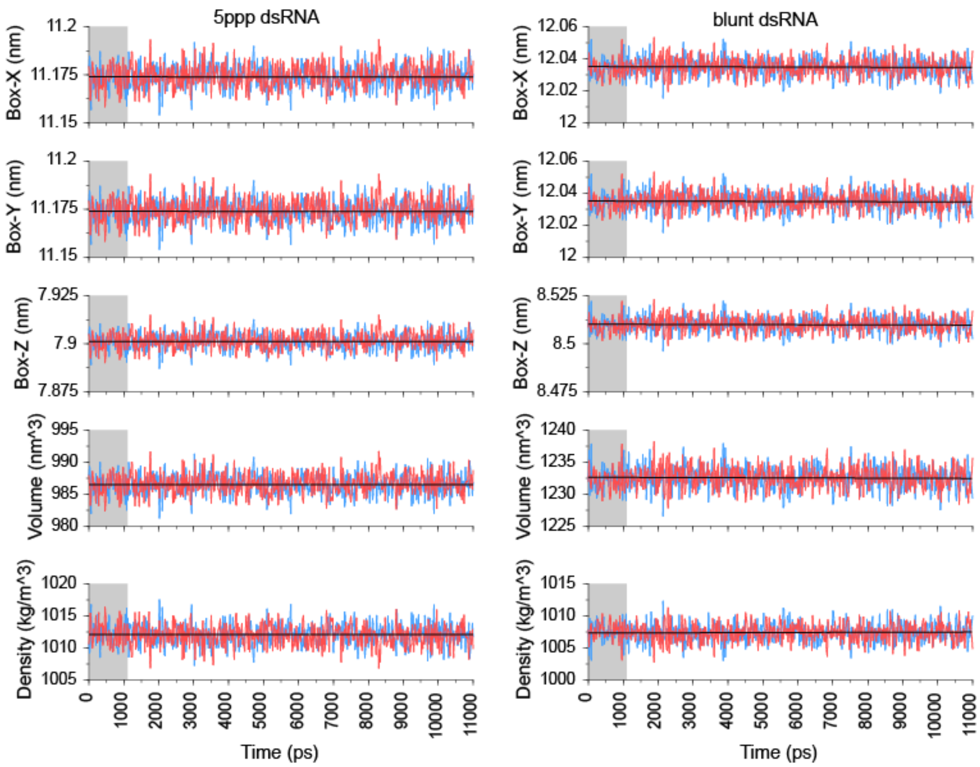

Figure S12 Part 1. ancLGP2.

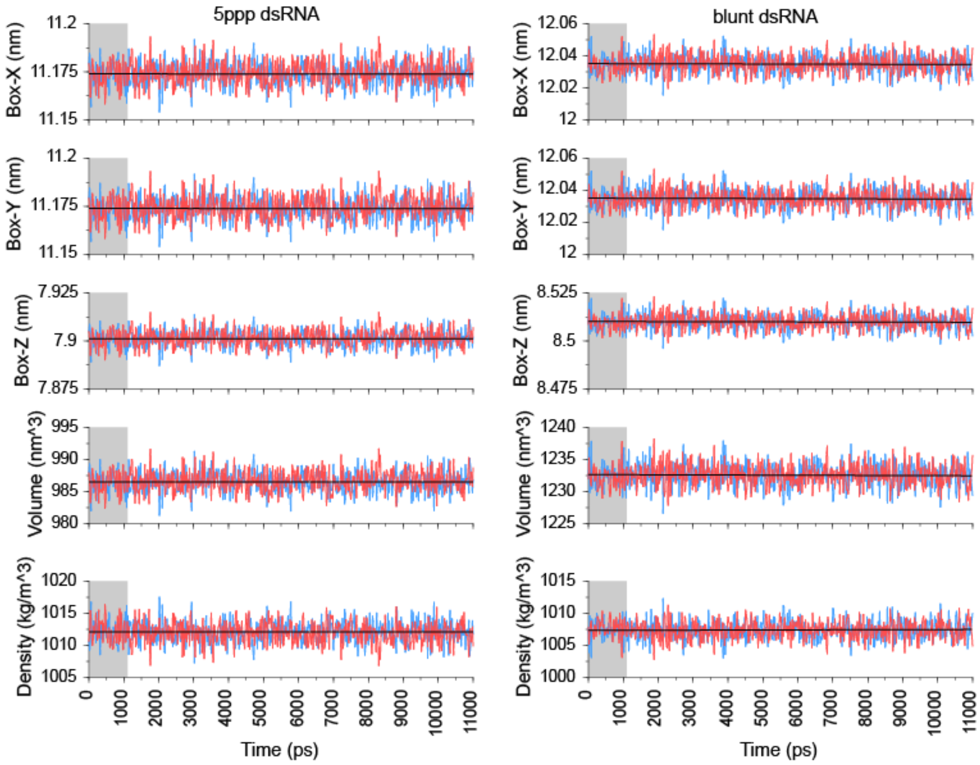

Figure S12 Part 2. ancMDA5.

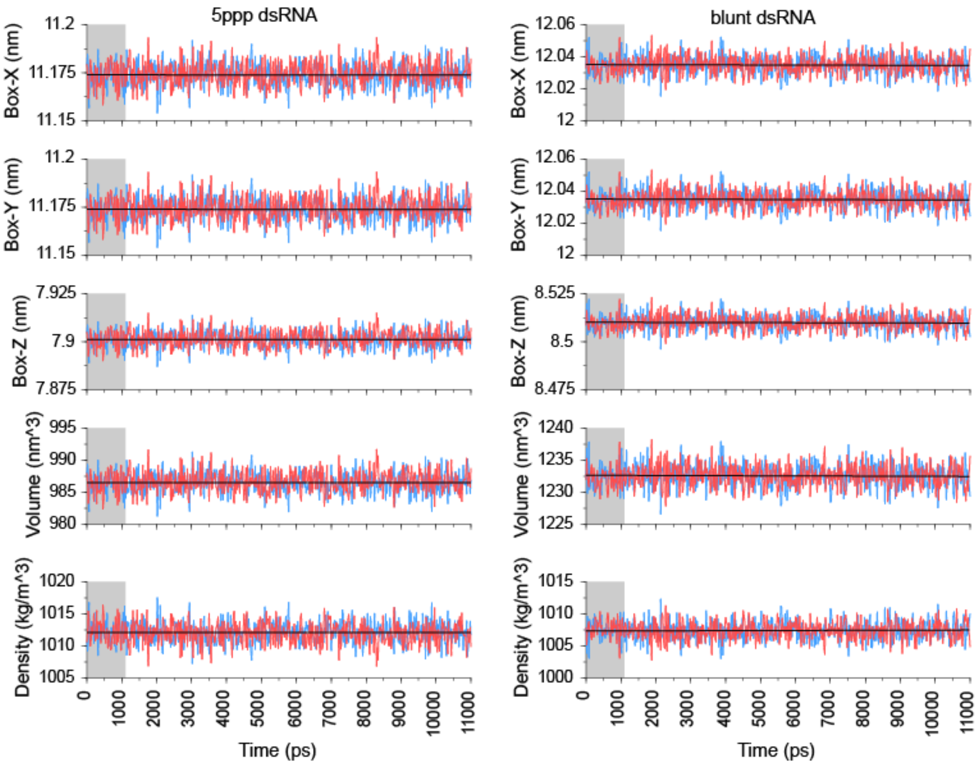

Figure S12 Part 3. ancMDA5/LGP2b.

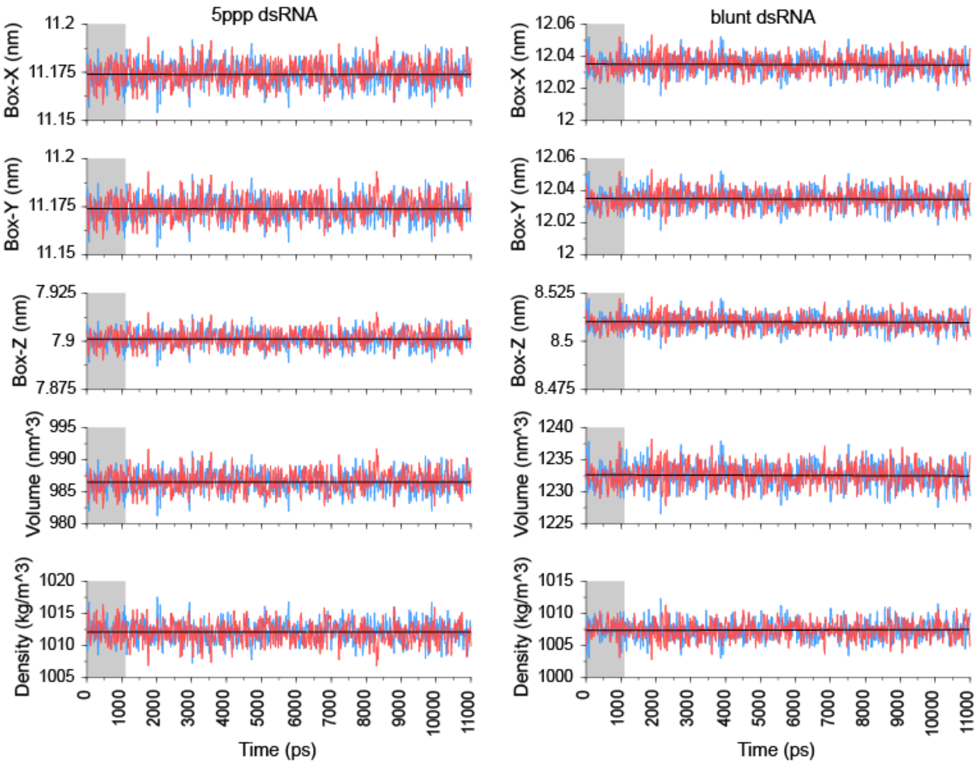

Figure S12 Part 4. ancMDA5/LGP2a.

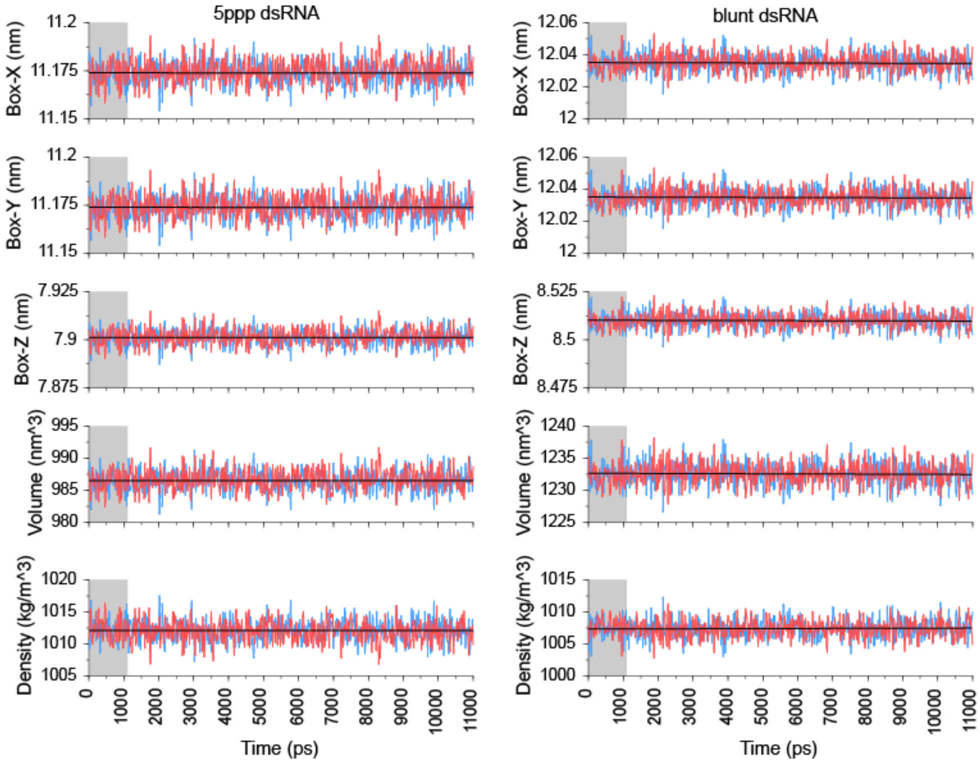

Figure S12 Part 5. ancRIG-I.

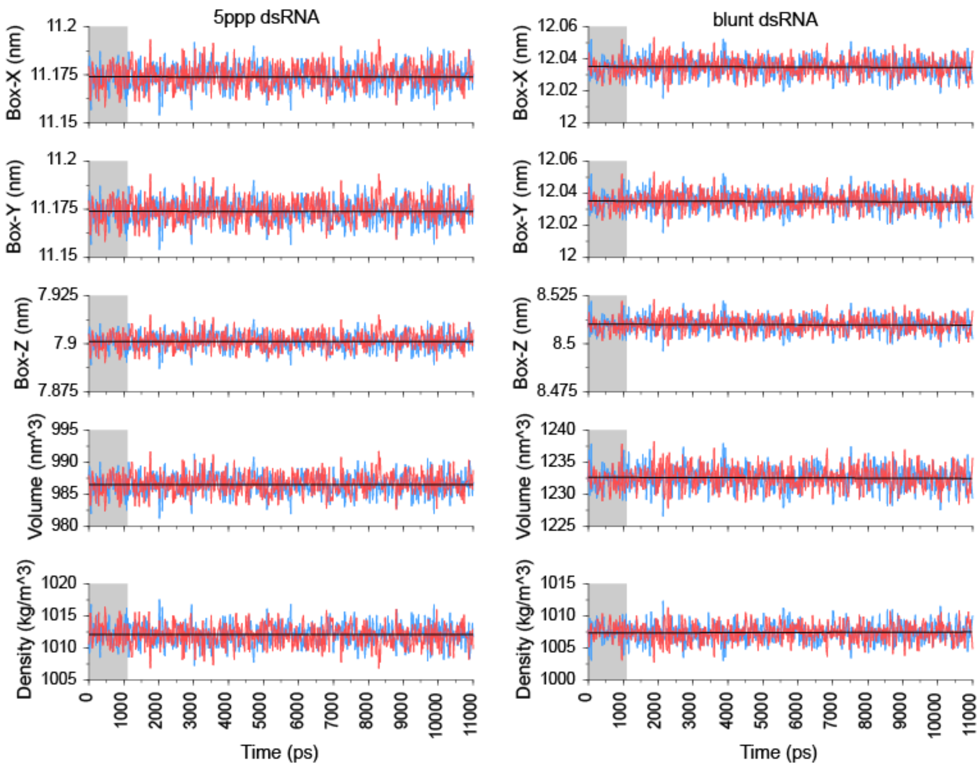

Figure S12 Part 6. ancRLR.

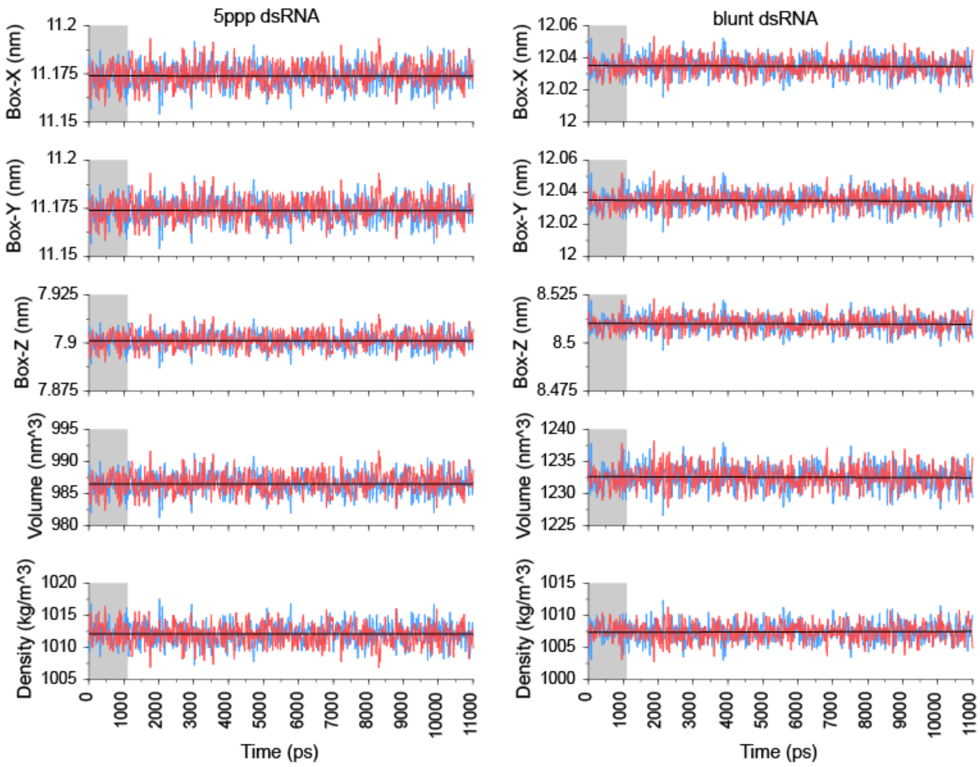

Figure S12 Part 7. ancMDA5/LGP2a^{H63S}.

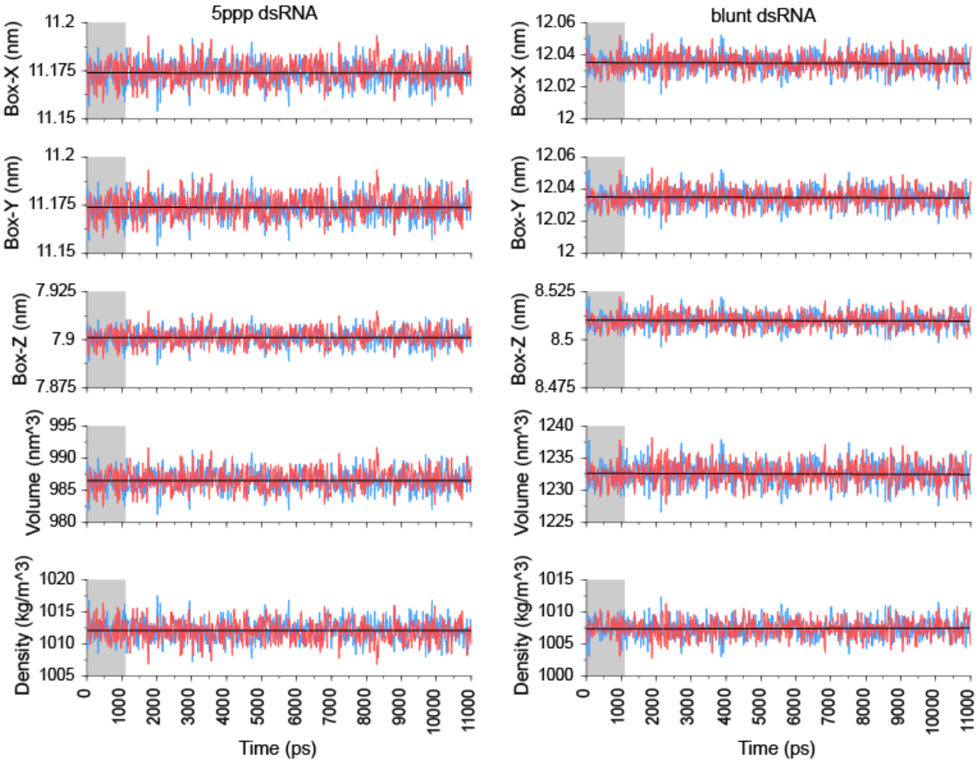

Figure S12 Part 8. ancMDA5/LGP2a$^{TEE47\Delta EK}$.

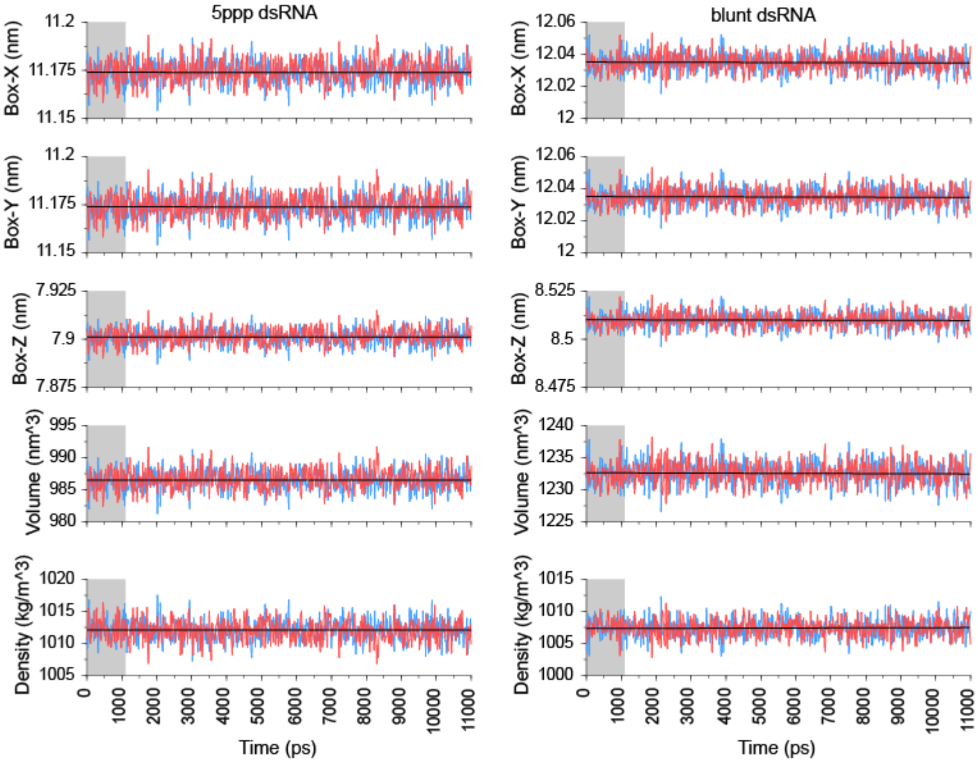

Figure S12 Part 9. ancRLR$^{\Delta EK47TEE,K88S}$.

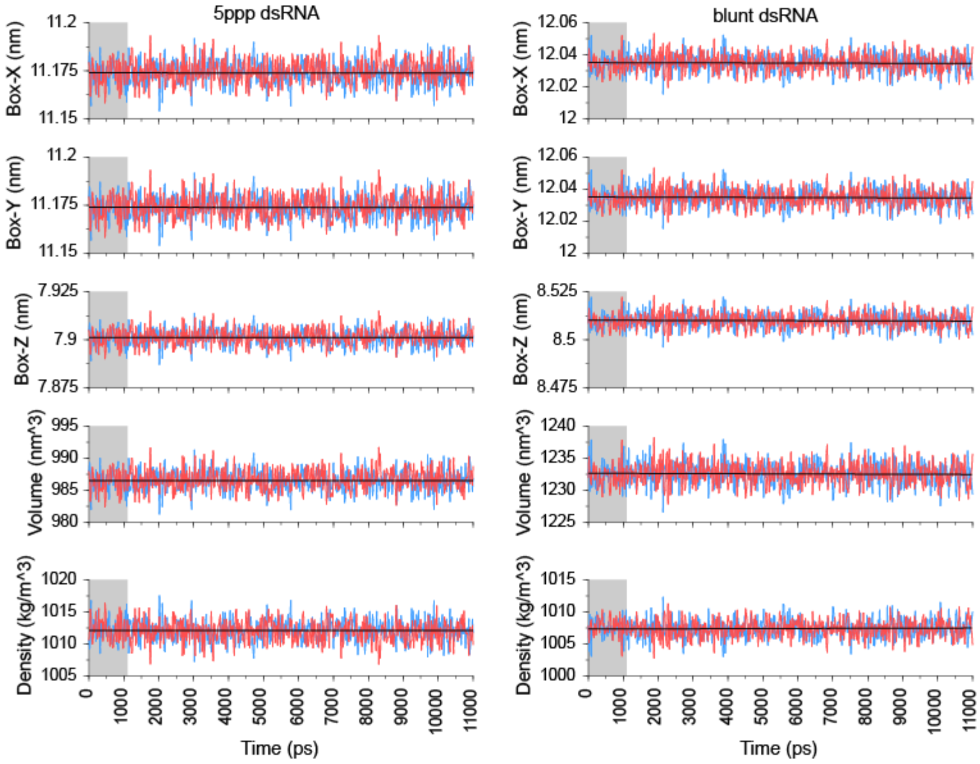

Figure S12 Part 10. Human LGP2.

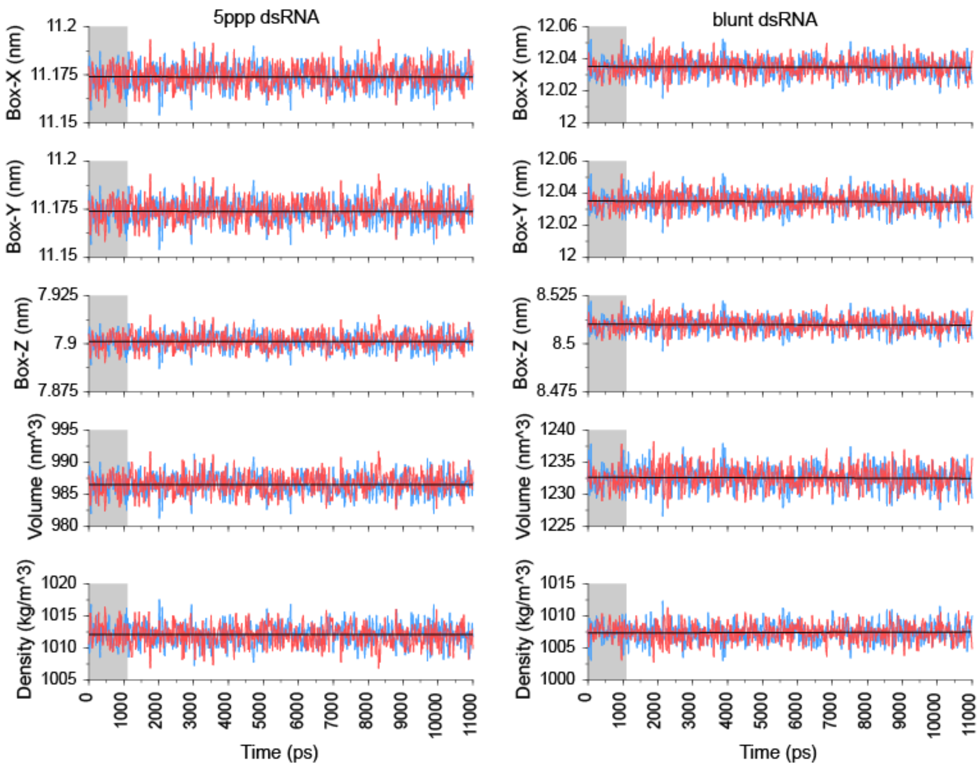

Figure S12 Part 11. Human MDA5.

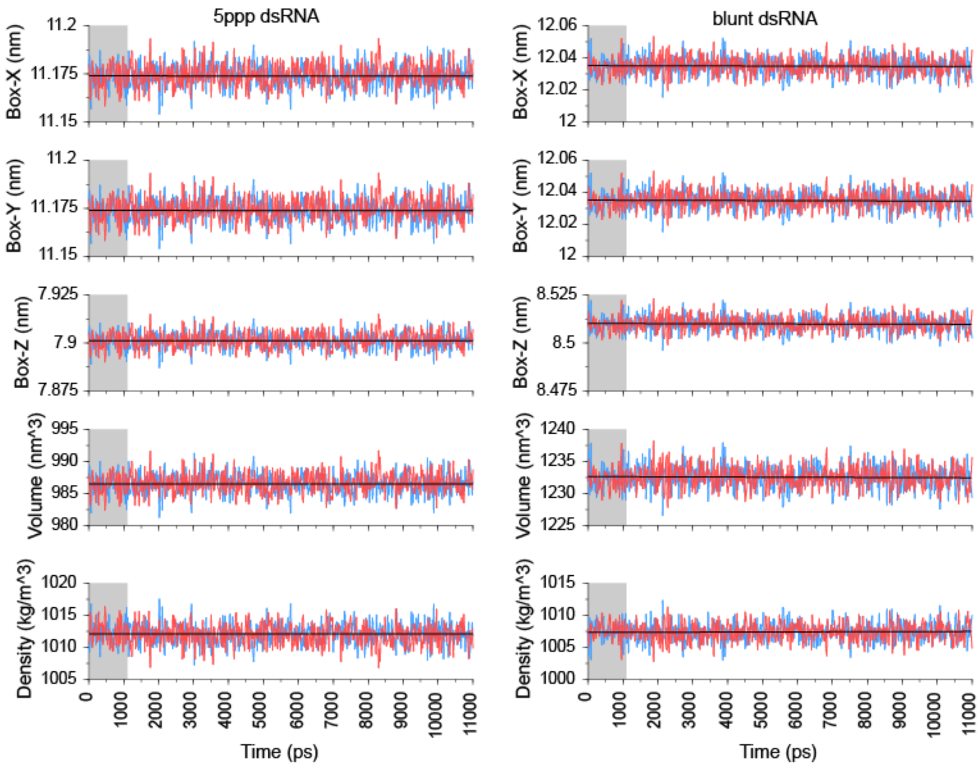

Figure S12 Part 12. Human RIG-I.

Figure S12. Volumetric parameters were well-behaved across molecular dynamics simulations of human and ancestral RLR RDs bound to 5'ppp and blunt-ended dsRNA. We plot the X, Y and Z dimensions of the simulated solvent box, as well as its volume and density, across the time course of each molecular dynamics simulation. Red and blue series indicate independent simulations of two RD proteins bound to either end of a double-stranded RNA molecule. We verified that characteristics of the RD-RNA complexes were less correlated between RDs in the same simulation than they were across simulations and considered each RD-RNA complex as an independent replicate for further analysis. Gray box indicates the first nanosecond of simulation, which we excluded from downstream analyses.

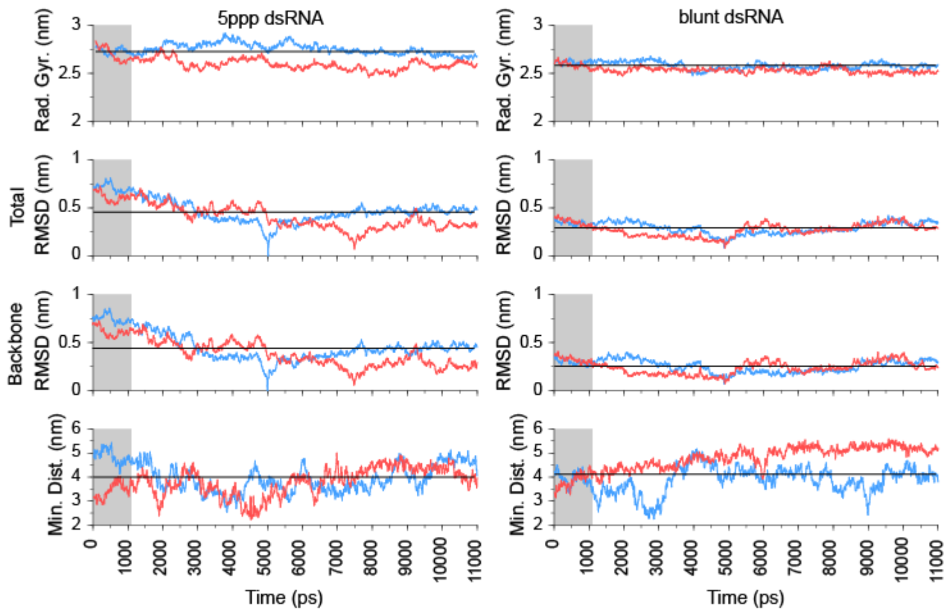

Figure S13 Part 1. ancLGP2.

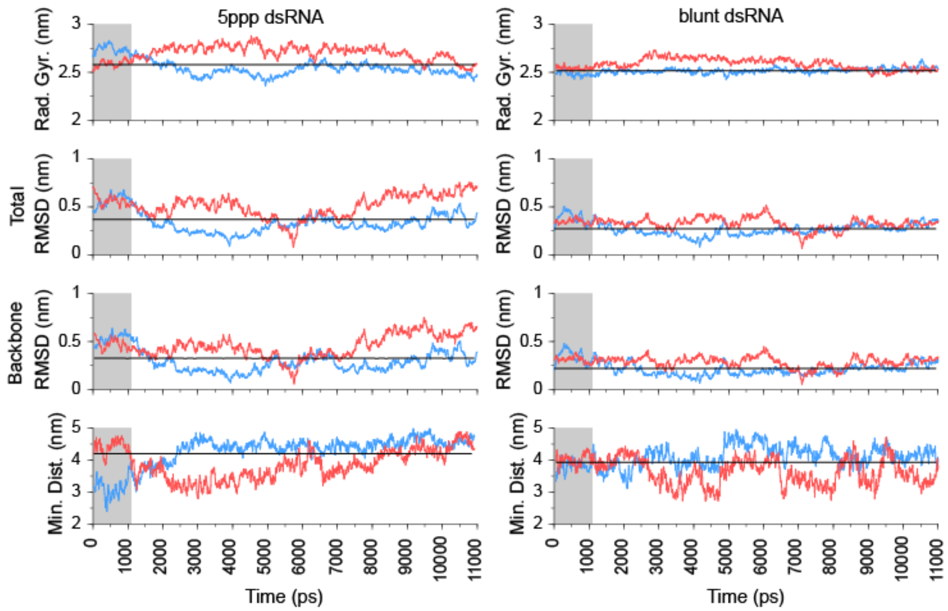

Figure S13 Part 2. ancMDA5.

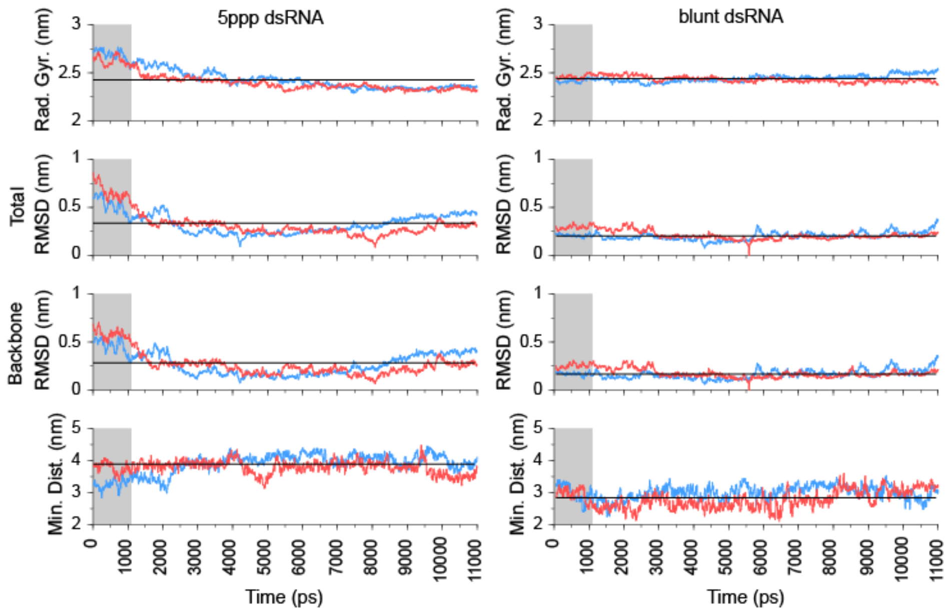

Figure S13 Part 3. ancMDA5/LGP2b.

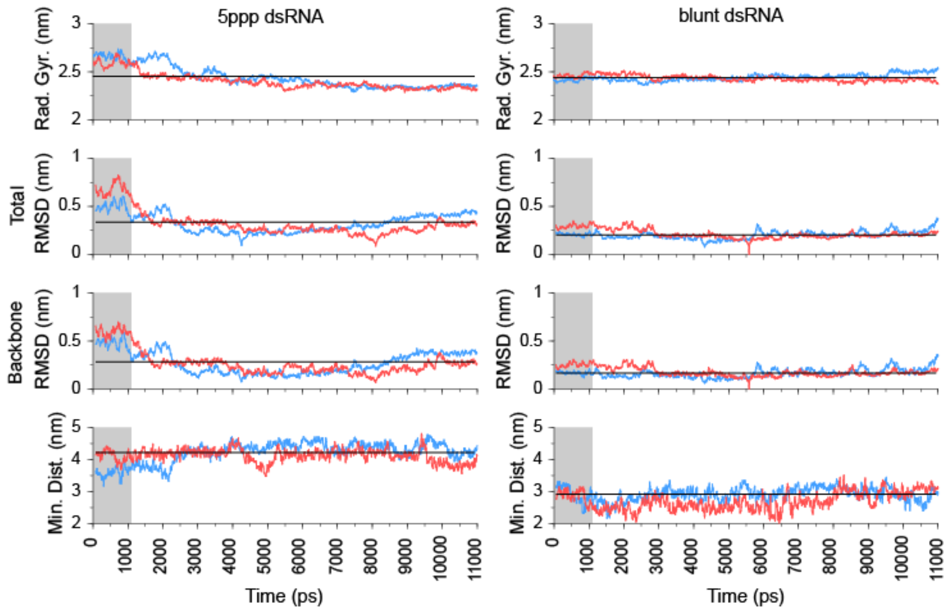

Figure S13 Part 4. ancMDA5/LGP2a.

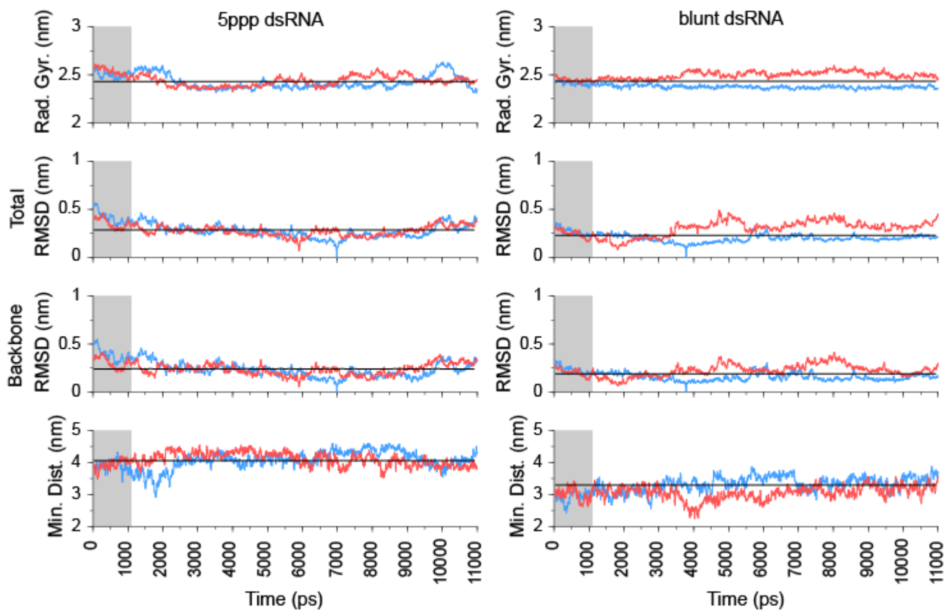

Figure S13 Part 5. ancRIG-I.

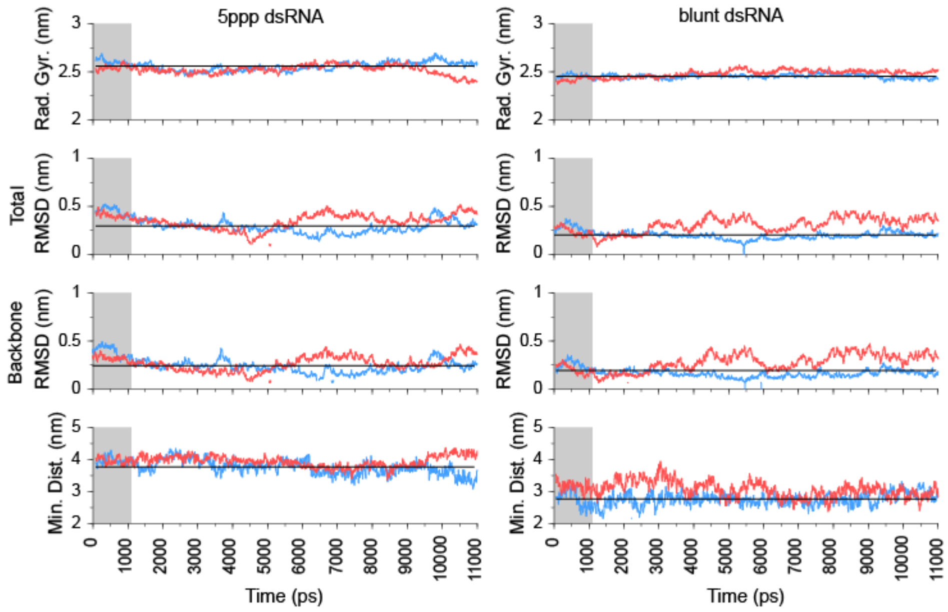

Figure S13 Part 6. ancRLR.

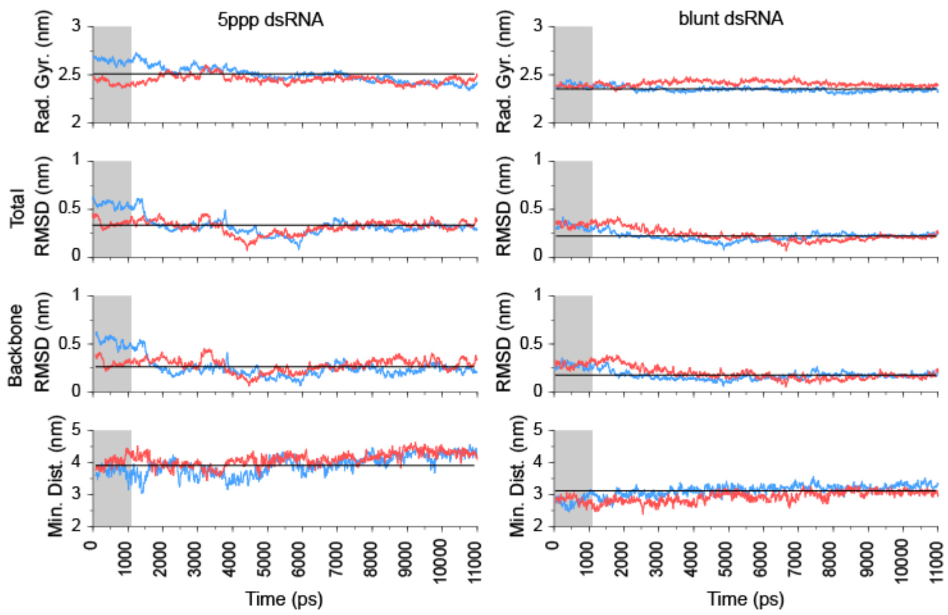

Figure S13 Part 7. ancMDA5/LGP2a^{H63S}.

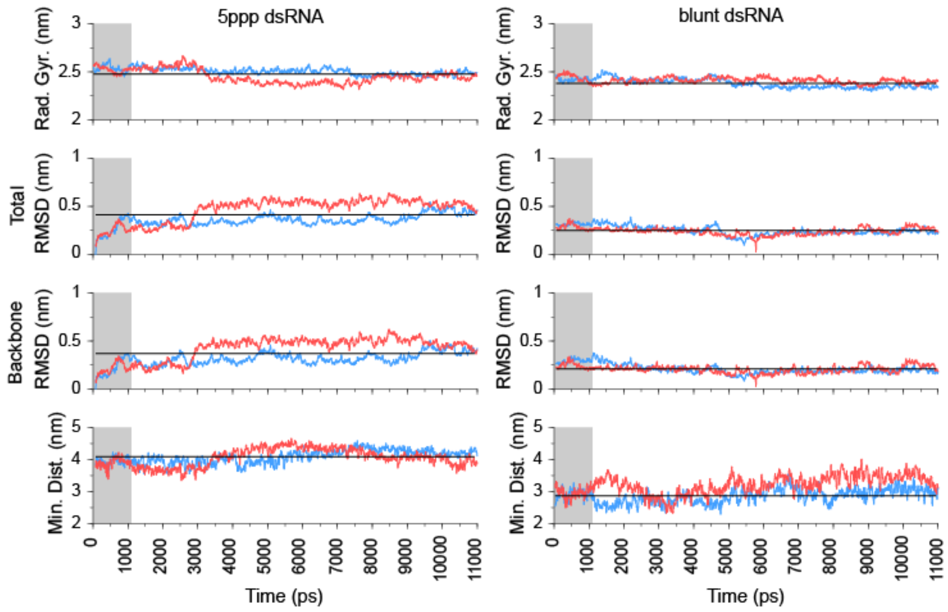

Figure S13 Part 8. ancMDA5/LGP2a$^{TEE47\Delta EK}$.

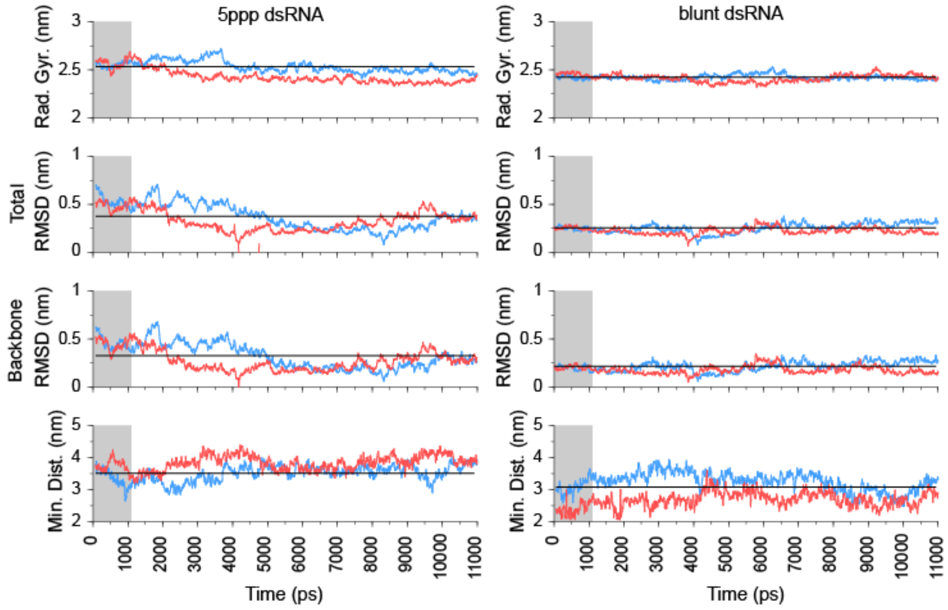

Figure S13 Part 9. ancRLR$^{\Delta EK47TEE,K88S}$.

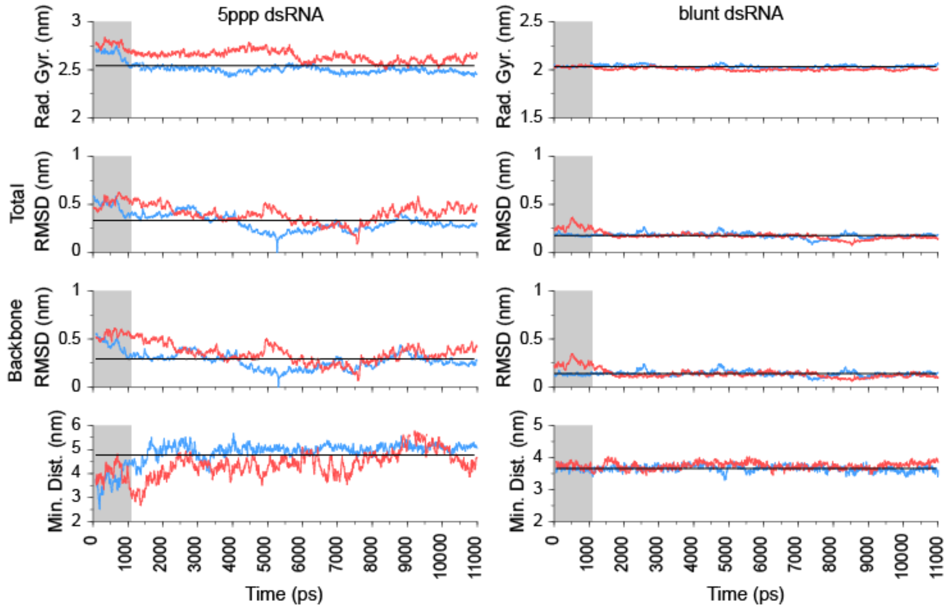

Figure S13 Part 10. Human LGP2.

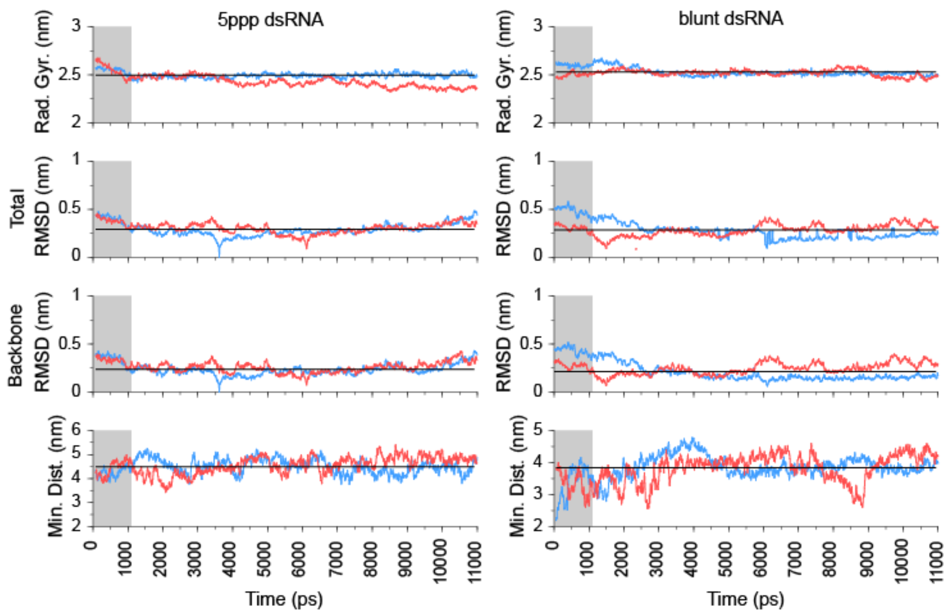

Figure S13 Part 11. Human MDA5.

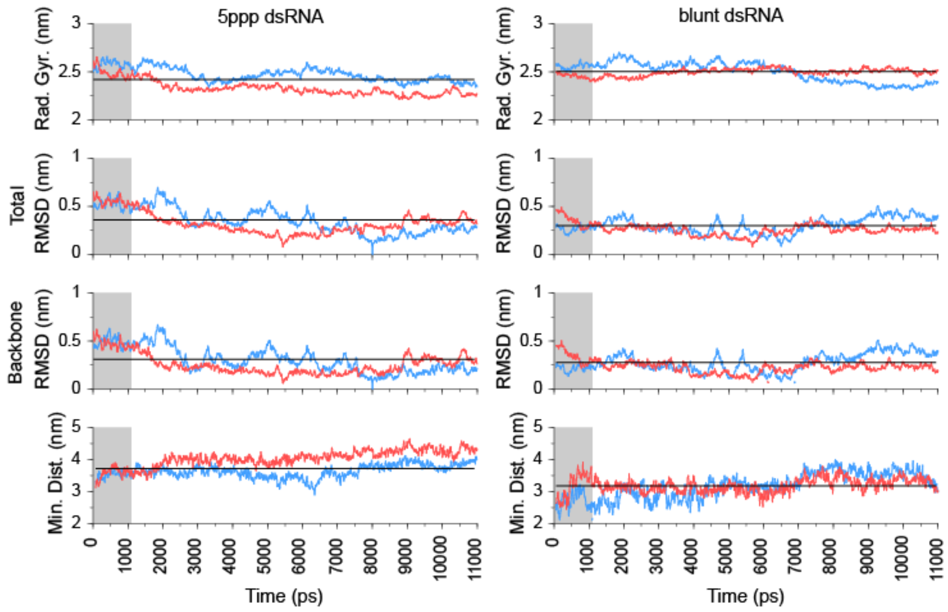

Figure S13 Part 12. Human RIG-I.

Figure S13. Physical characteristics of the RLR-RNA complex were well-behaved across molecular dynamics simulations of human and ancestral RLR RDs bound to 5'ppp and blunt-ended dsRNA. We plot the radius of gyration, total and background RMSD and minimum atom-atom distance, across the time course of each molecular dynamics simulation. Red and blue series indicate independent simulations of two RD proteins bound to either end of a double-stranded RNA molecule. We verified that characteristics of the RD-RNA complexes were less correlated between RDs in the same simulation than they were across simulations and considered each RD-RNA complex as an independent replicate for further analysis. Gray box indicates the first nanosecond of simulation, which we excluded from downstream analyses.

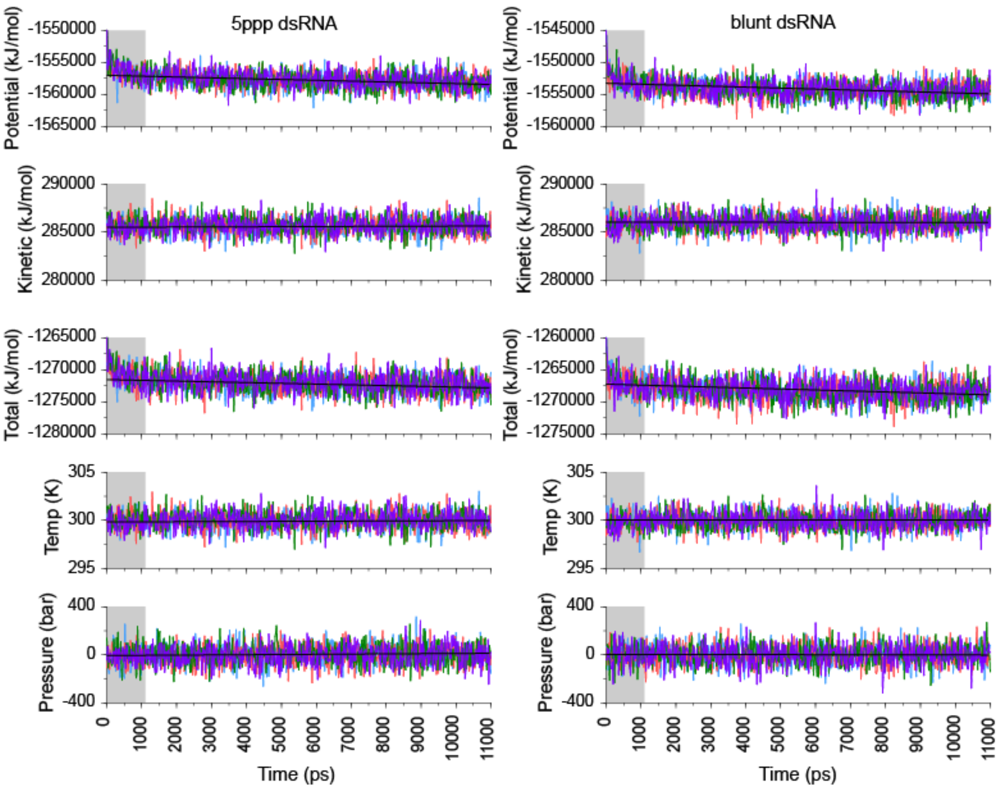

Figure S14 Part 1. ancMDA5/LGP2b.

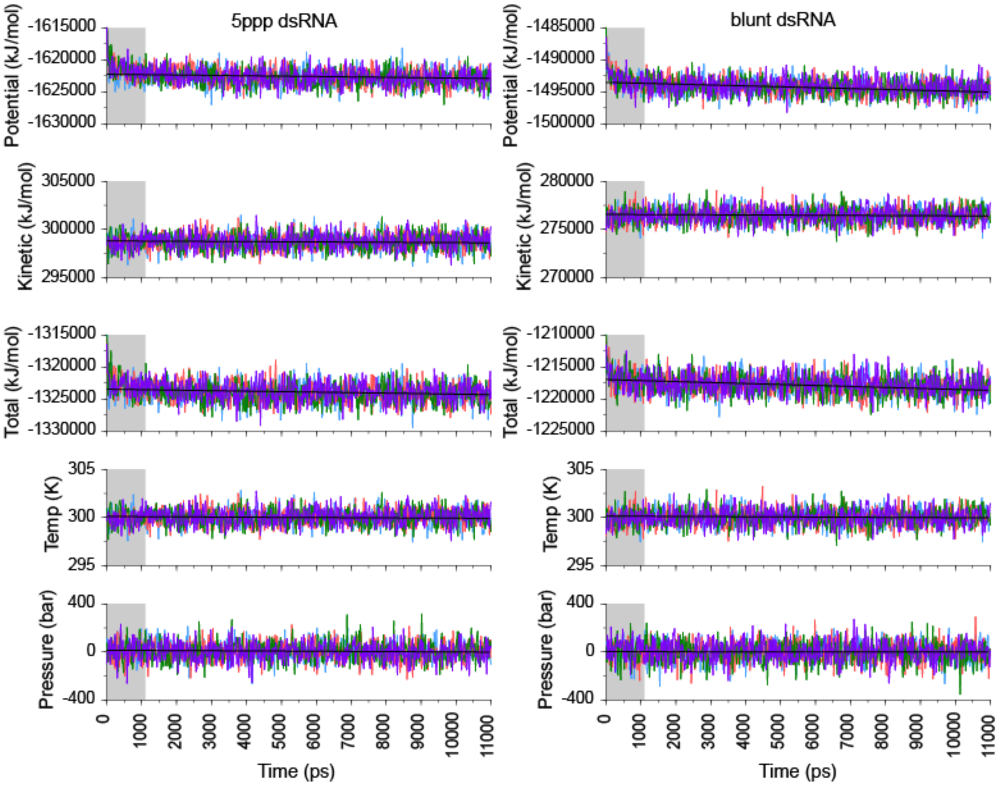

Figure S14 Part 2. ancMDA5/LGP2a.

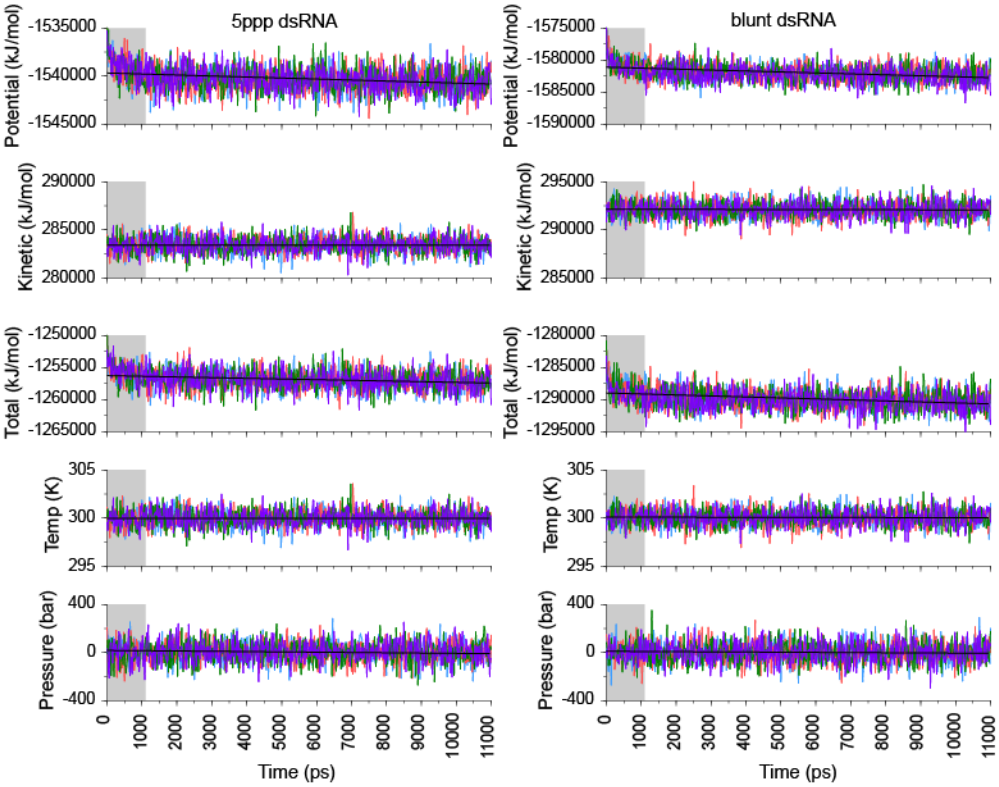

Figure S14 Part 3. ancRLR.

Figure S14. Energy parameters were well-behaved across molecular dynamics simulations of ancestral RLR helicase+pincer+RD domains bound to 5'ppp and blunt-ended dsRNA. We plot potential, kinetic and total energy, as well as temperature and pressure, across the time course of each molecular dynamics simulation. Each series indicates an independent simulation of one helicase+pincer+RD protein bound to a double-stranded RNA molecule. Gray box indicates the first nanosecond of simulation, which we excluded from downstream analyses.

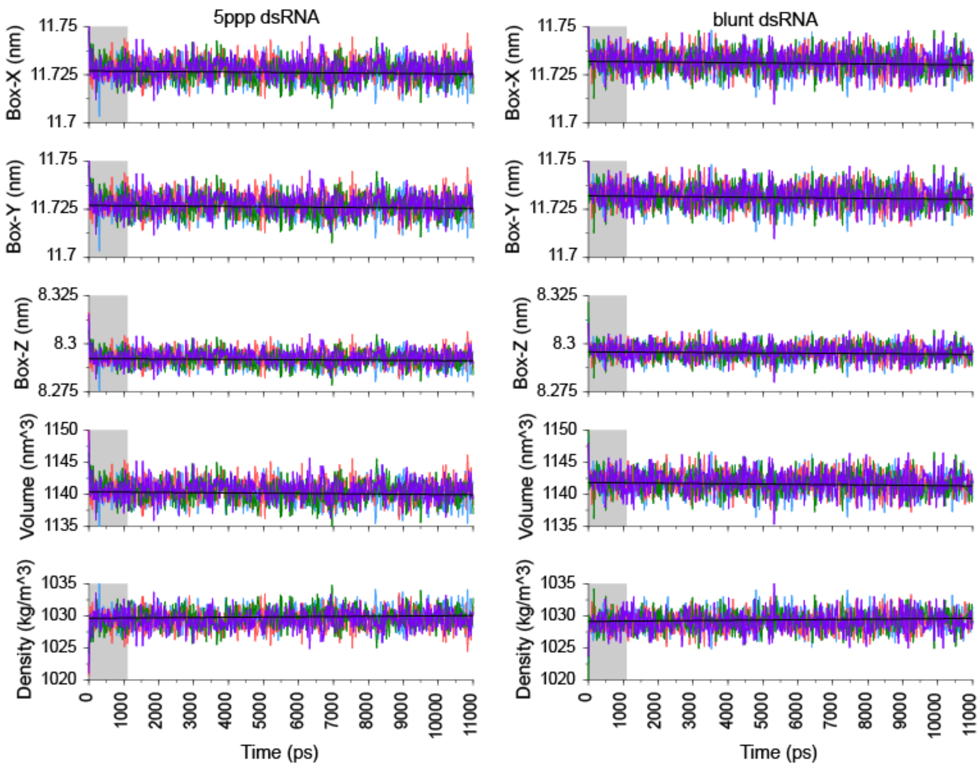

Figure S15 Part 1. ancMDA5/LGP2b.

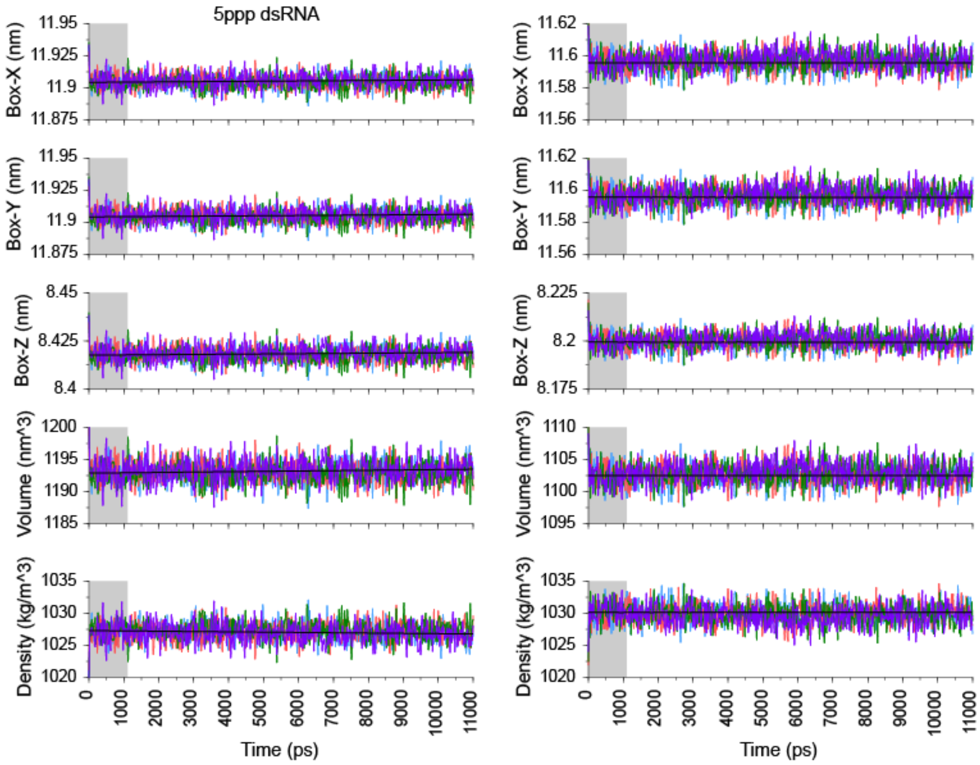

Figure S15 Part 2. ancMDA5/LGP2a.

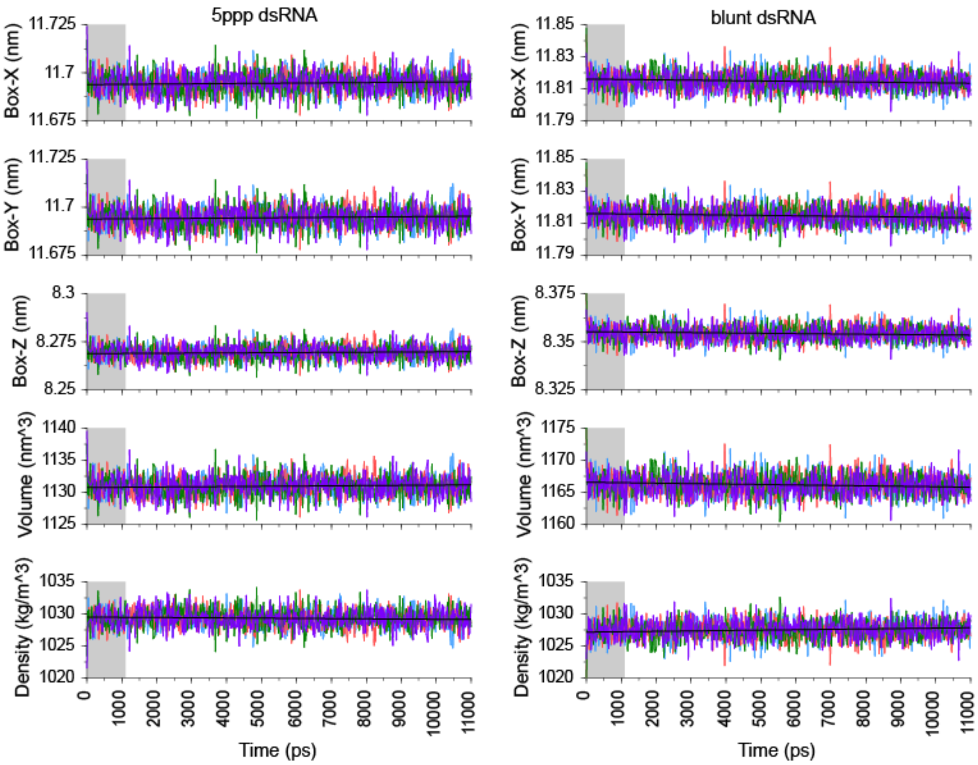

Figure S15 Part 3. ancRLR.

Figure S15. Volumetric parameters were well-behaved across molecular dynamics simulations of ancestral RLR helicase+pincer+RD domains bound to 5'ppp and blunt-ended dsRNA. We plot the X, Y and Z dimensions of the simulated solvent box, as well as its volume and density, across the time course of each molecular dynamics simulation. Each series indicates an independent simulation of one helicase+pincer+RD protein bound to a double-stranded RNA molecule. Gray box indicates the first nanosecond of simulation, which we excluded from downstream analyses.

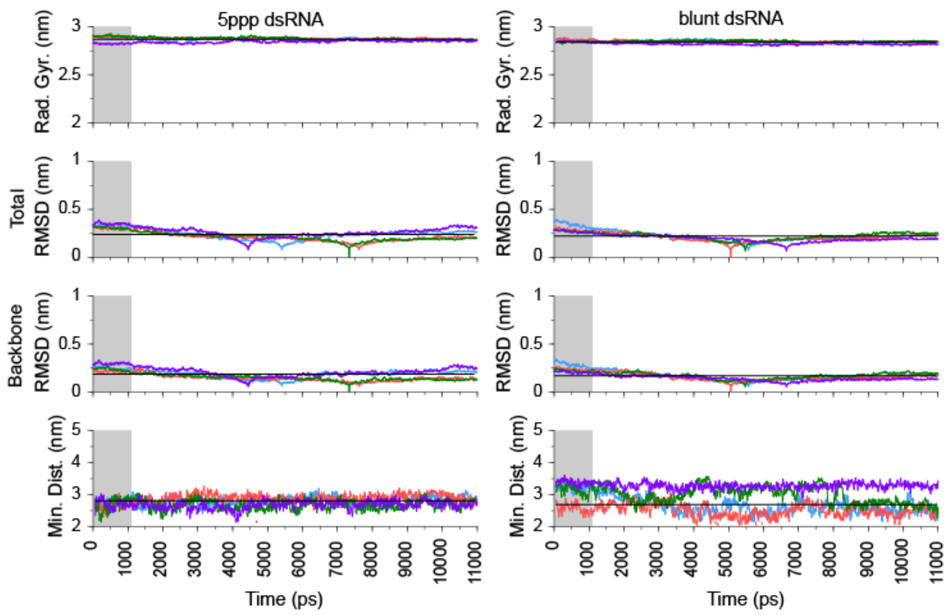

Figure S16 Part 1. ancMDA5/LGP2b.

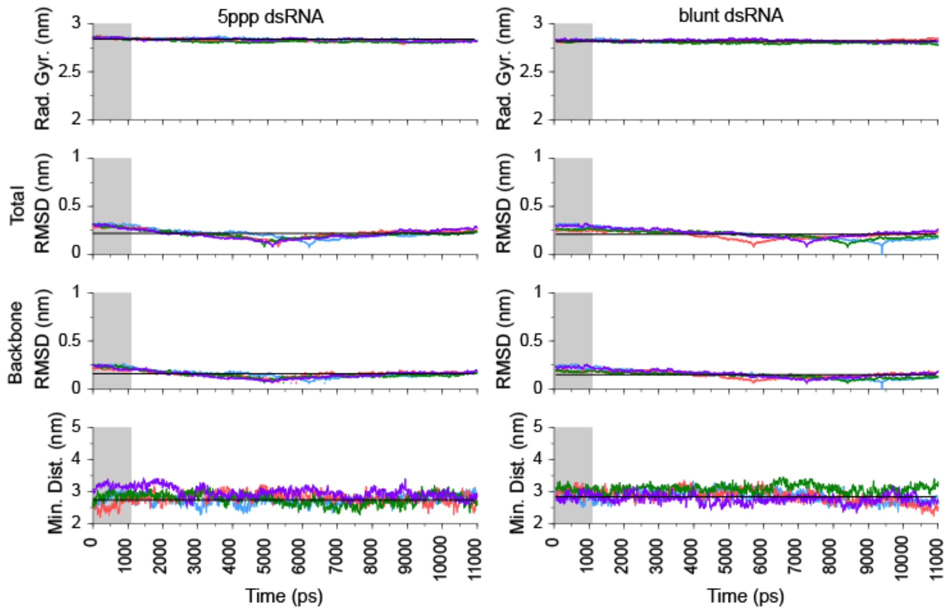

Figure S16 Part 2. ancMDA5/LGP2a.

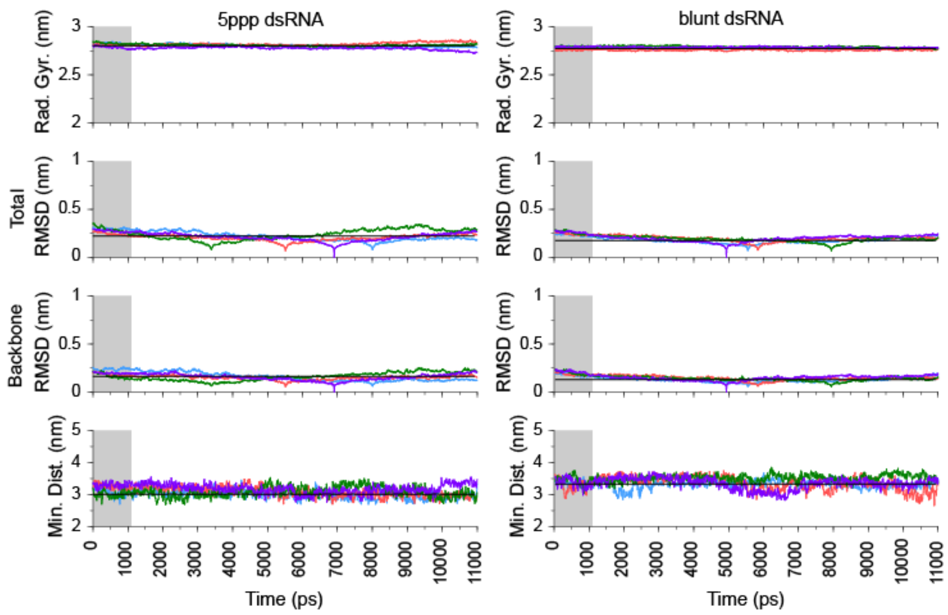

Figure S16 Part 3. ancRLR.

Figure S16. Physical characteristics of the RLR-RNA complex were well-behaved across molecular dynamics simulations of ancestral RLR helicase+pincer+RD domains bound to 5'ppp and blunt-ended dsRNA. We plot the radius of gyration, total and background RMSD and minimum atom-atom distance, across the time course of each molecular dynamics simulation. Each series indicates an independent simulation of one helicase+pincer+RD protein bound to a double-stranded RNA molecule. Gray box indicates the first nanosecond of simulation, which we excluded from downstream analyses.

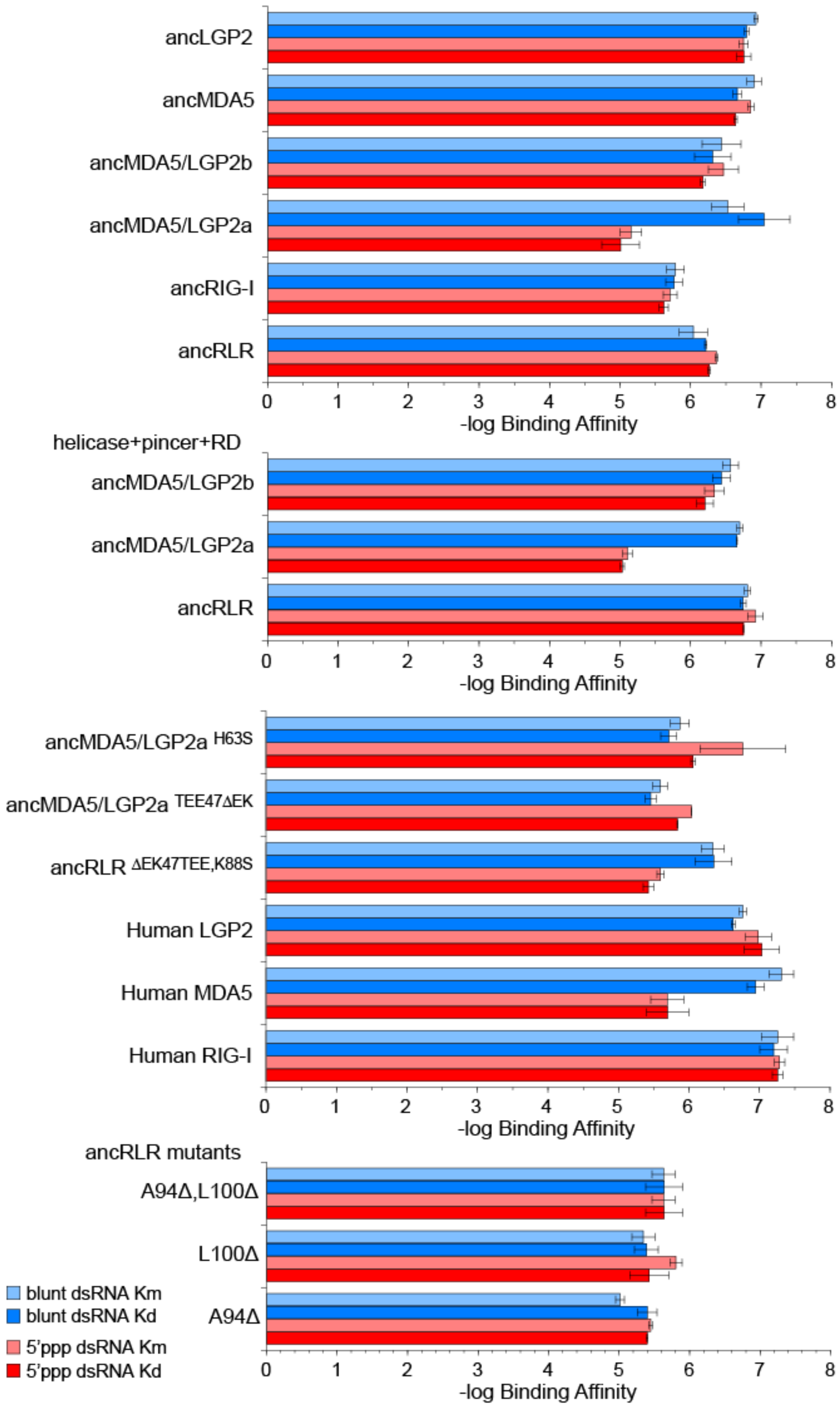

Figure S17. We summarize binding kinetics results for all ancestral, human and mutant RLR constructs used in the study. We measured the steady-state binding affinities (Kd, dark series) and initial RNA binding rates (Km, light series) of ancestral, mutant, and human RLRs bound to blunt-ended (blue) and 5'ppp dsRNA (red) using a label-free kinetics assay (see Methods). For each RLR and RNA type we plot –log binding affinities, with longer bars indicating stronger affinity. Standard errors over three replicates are indicated. Constructs other than those listed as "helicase+pincer+RD" consisted of only the RD domain.

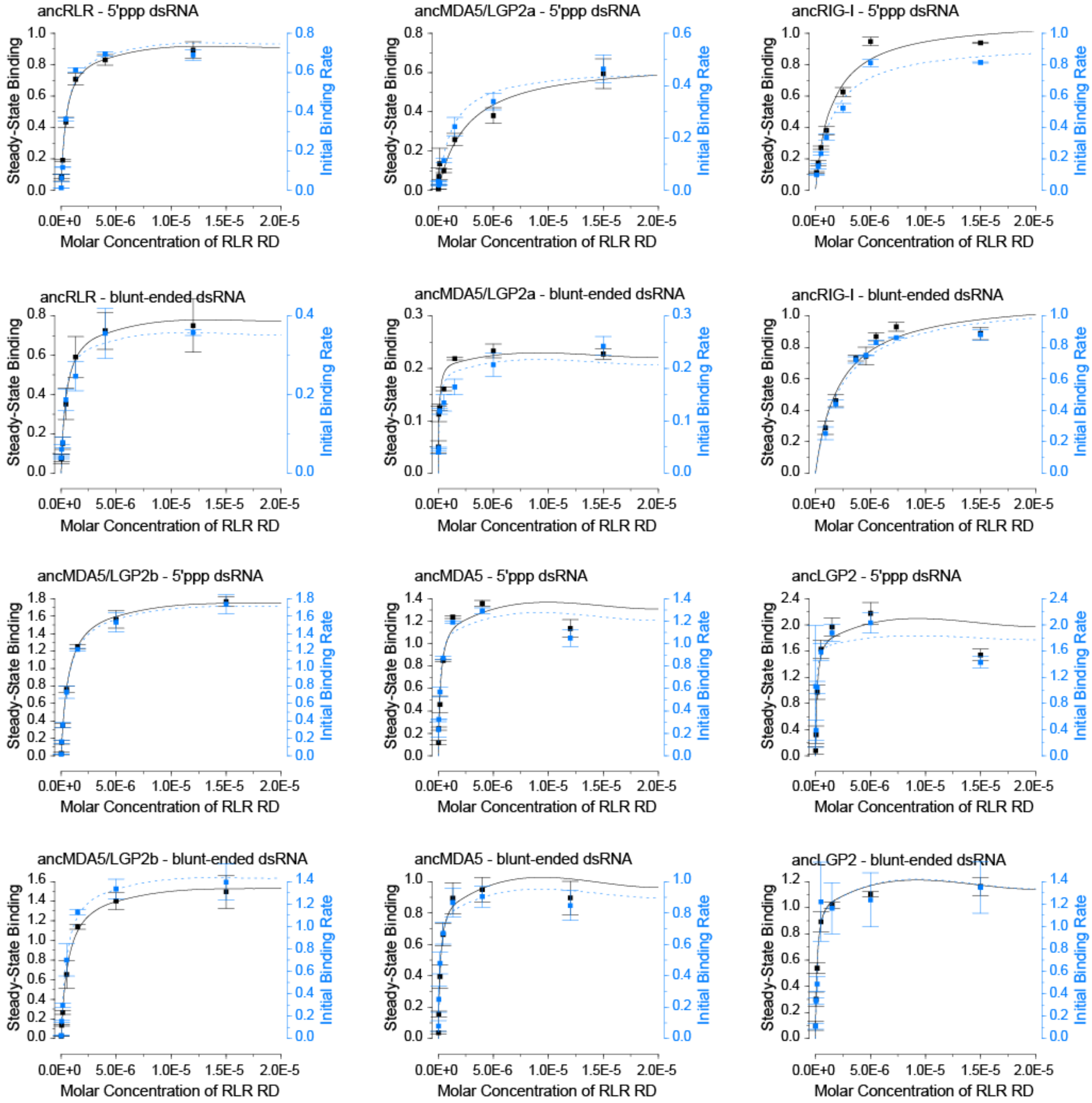

Figure S18, Part 1.

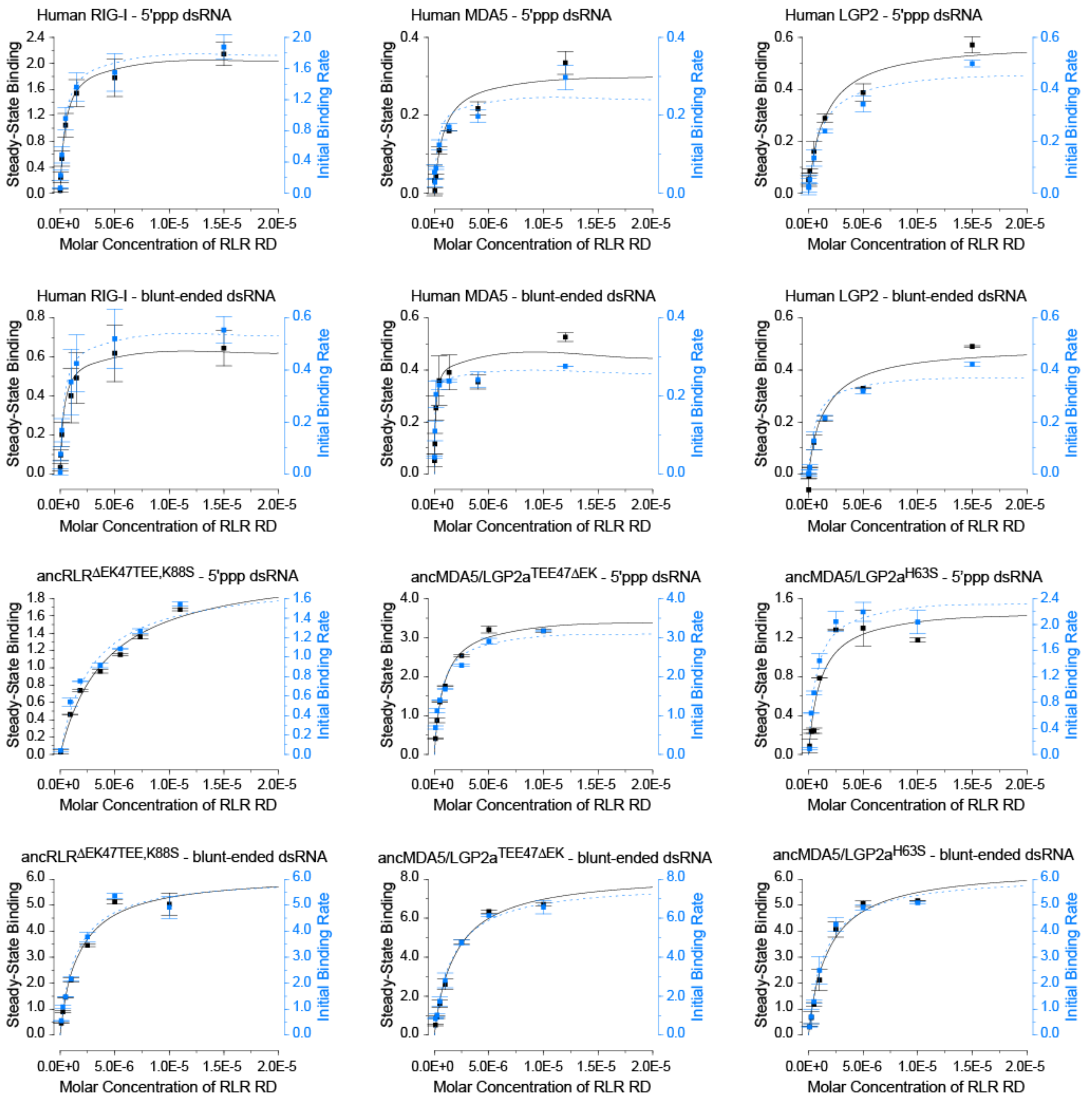

Figure S18. We show binding kinetics curves for all ancestral, human and mutant RLR RDs used in the study. We measured the steady-state binding affinities (Kd, black dots) and initial RNA binding rates (Km, blue dots) of ancestral, mutant and human RLR RDs bound to blunt-ended and 5'ppp dsRNA molecules (see Methods). For each RD and RNA, we plot the average and standard error in RD-RNA binding across RD concentration. We used nonlinear regression to fit a single-site binding curve to each plot.

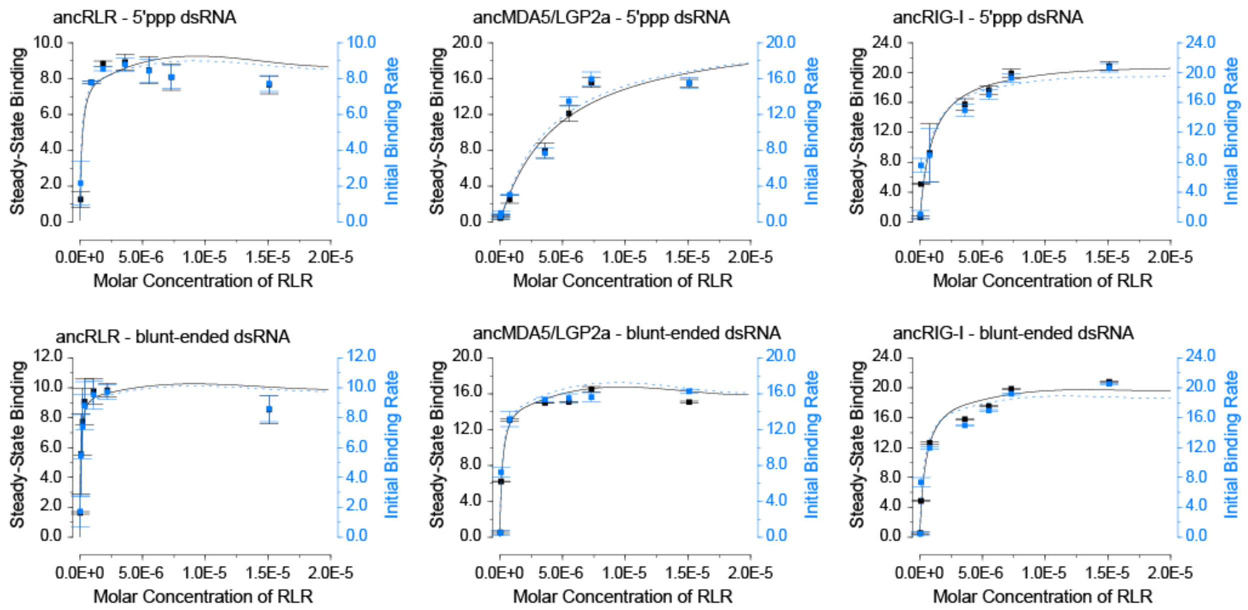

Figure S19. We show binding kinetics curves for all ancestral RLR helicase+pincer+RDs used in the study. We measured the steady-state binding affinities (Kd, black dots) and initial RNA binding rates (Km, blue dots) of ancestral RLR helicase+pincer+RD constructs bound to blunt-ended and 5'ppp dsRNA molecules (see Methods). For each RLR and RNA, we plot the average and standard error in RLR-RNA binding across RLR concentration. We used nonlinear regression to fit a single-site binding curve to each plot.

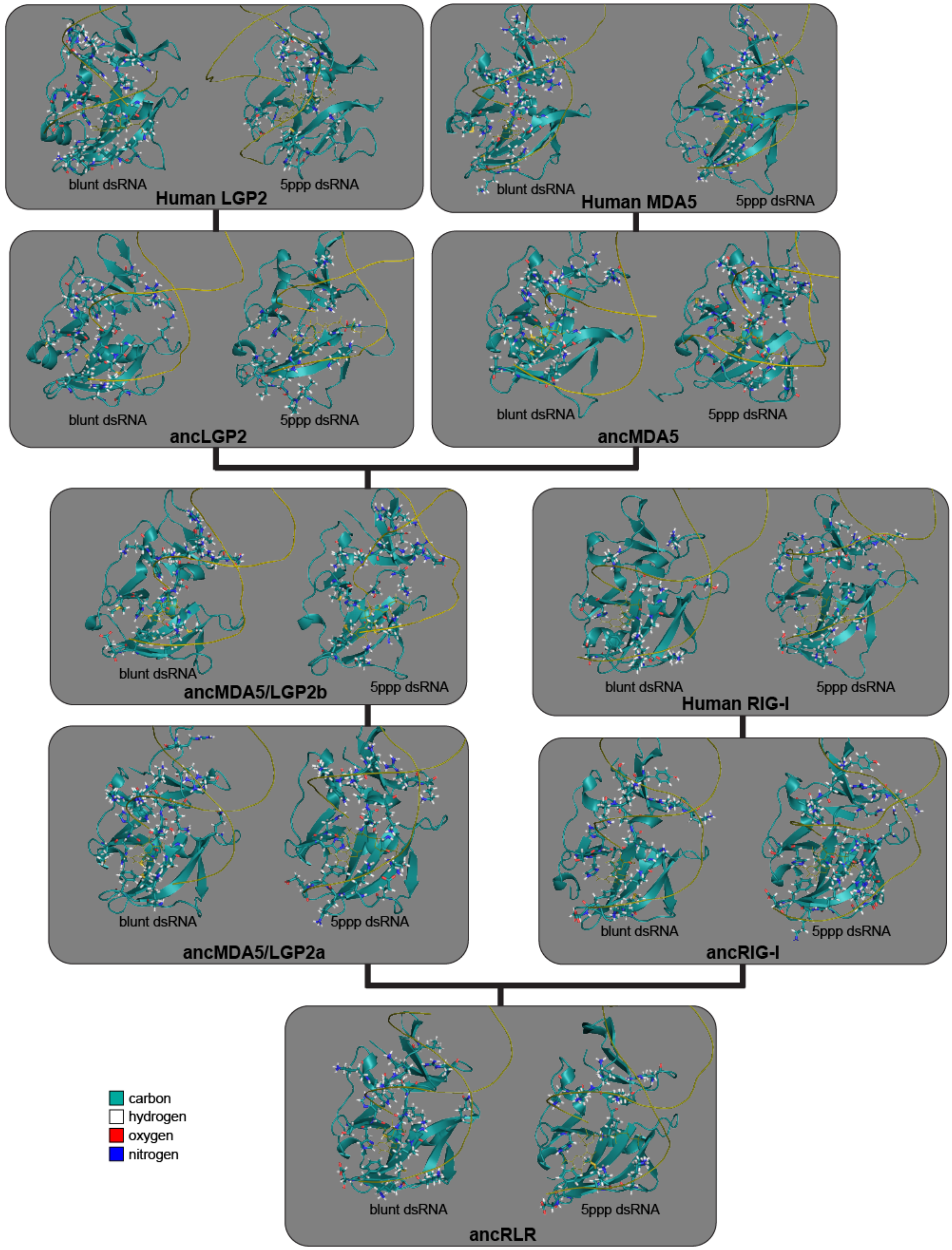

Figure S20. Ionizable residues nearby RNA ligands are heavily protonated during molecular dynamics simulations. We show the central structure of each RD-RNA from replicate molecular

dynamics simulations. Residues within 4 angstroms of the RNA are shown as stick models, with atoms colored according to type.

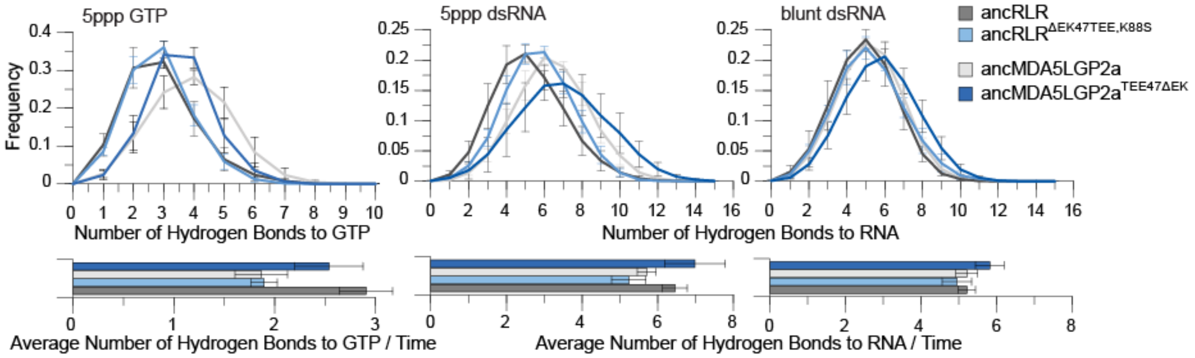

Figure S21. Hydrogen bonding between ancestral RNA recognition domains (RDs) and RNA ligands changes from ancRLR to ancMDA5/LGP2a. We performed replicate molecular dynamics simulations of each ancestral or mutant RLR RD bound to blunt-ended or 5'ppp dsRNA (see Methods). Top panels plot the proportion of simulation samples from which we observed each number of hydrogen bonds between the RD and its RNA ligand, with bars indicating standard errors. Bottom panels plot the average and standard error of the number of hydrogen bonds between the RD and its RNA ligand over all sampled molecular dynamics time points.

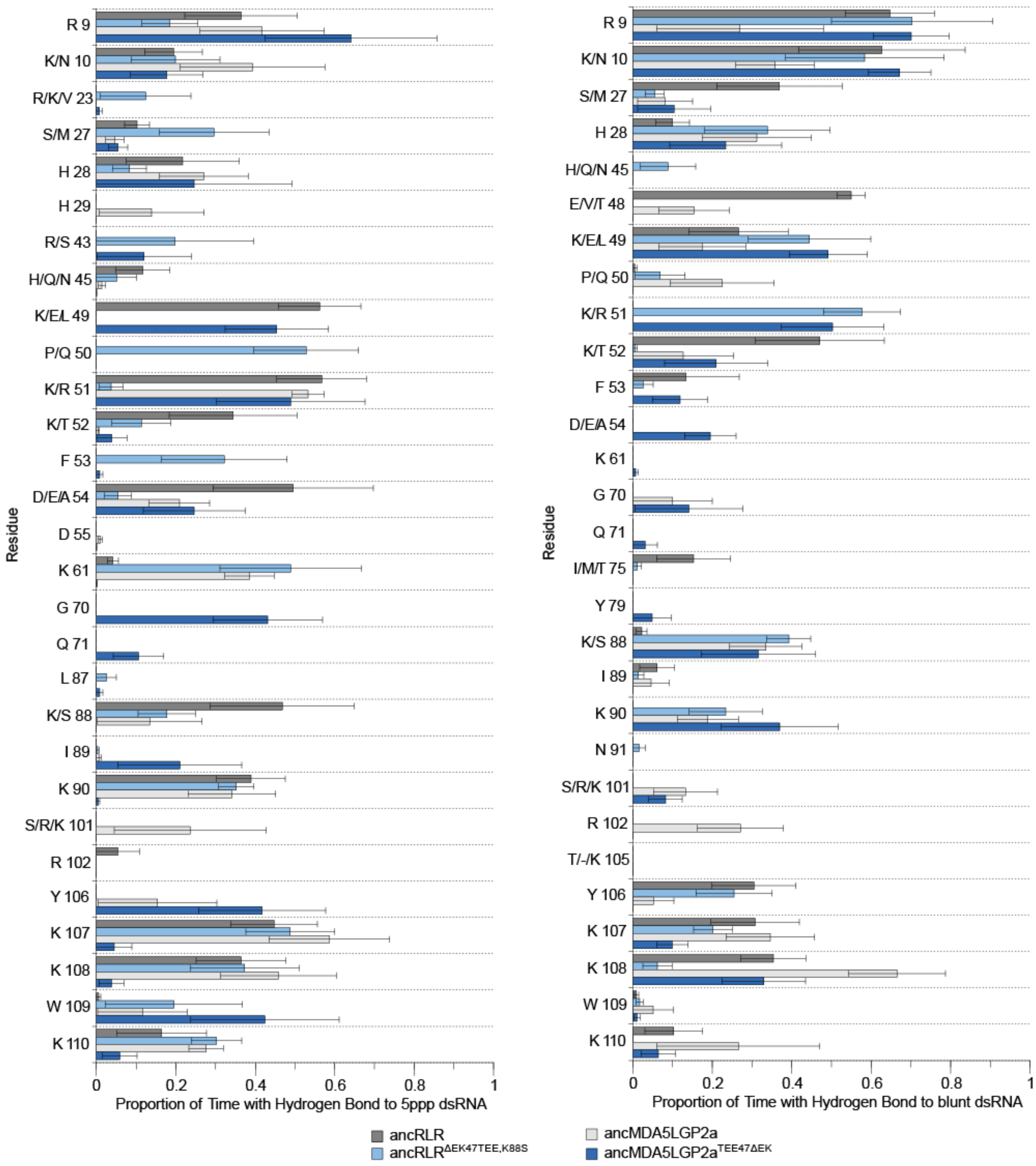

Figure S22. The capacity of specific residues to form hydrogen bonds to the RNA ligand changes between ancRLR and ancMDA5/LGP2a. We performed replicate molecular dynamics simulations of each RLR RD bound to blunt-ended and 5'ppp dsRNA (see Methods). We plot the average proportion of time each residue formed hydrogen bonds with its RNA ligand over the course of molecular dynamics simulations. Bars indicate standard errors over four replicate dynamics simulations.

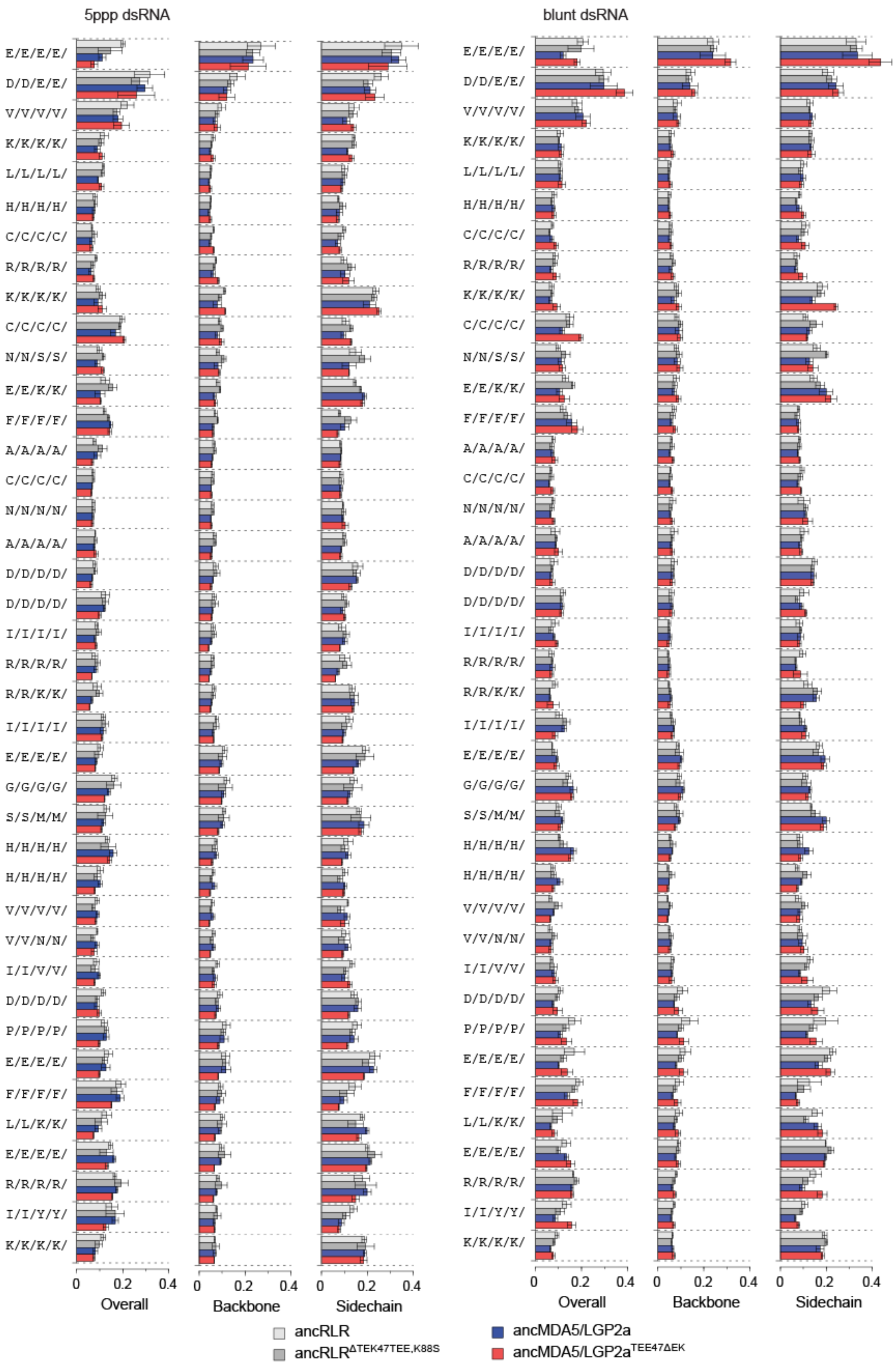

Figure S23, part 1.

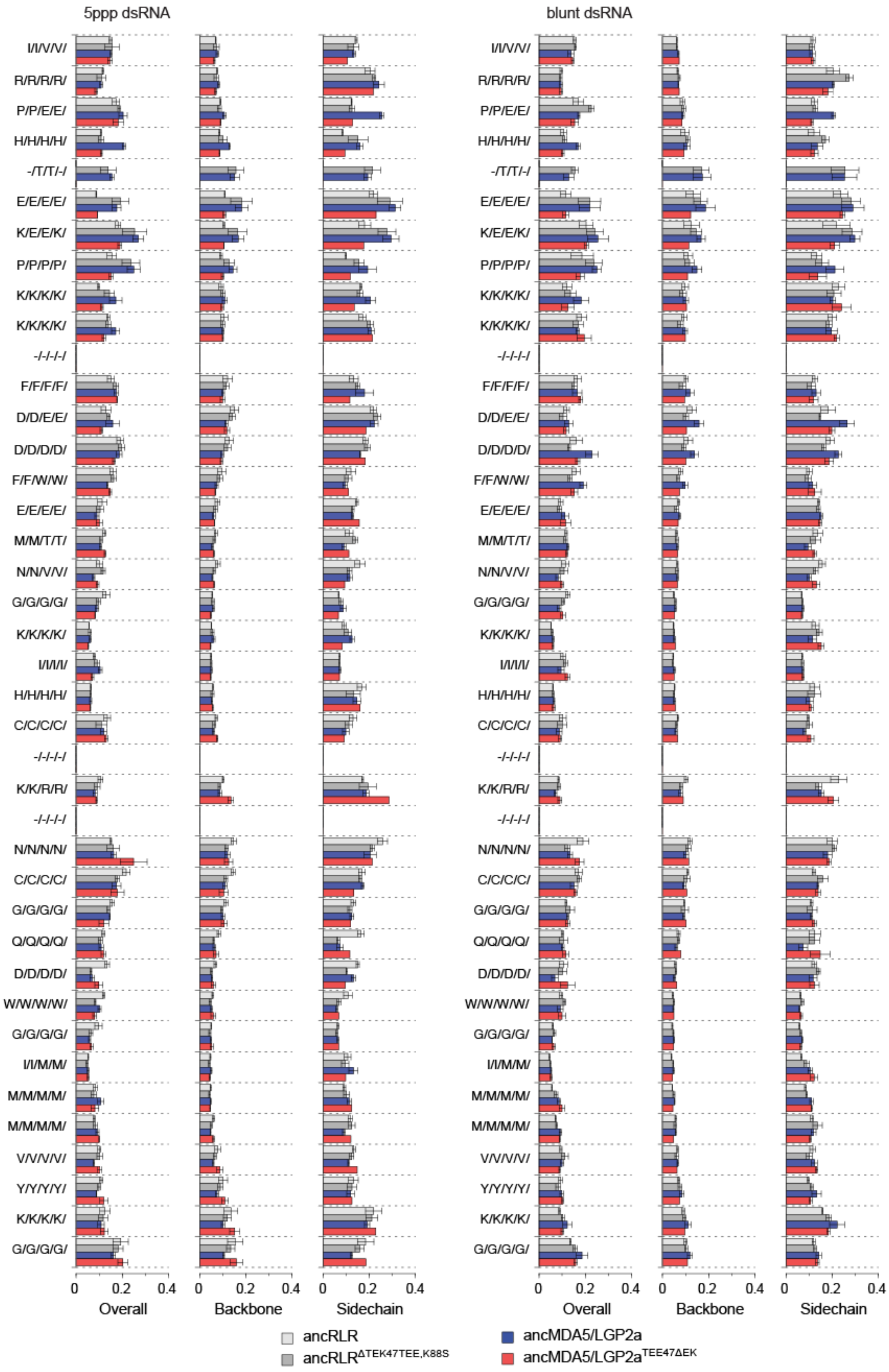

Figure S23, part 2.

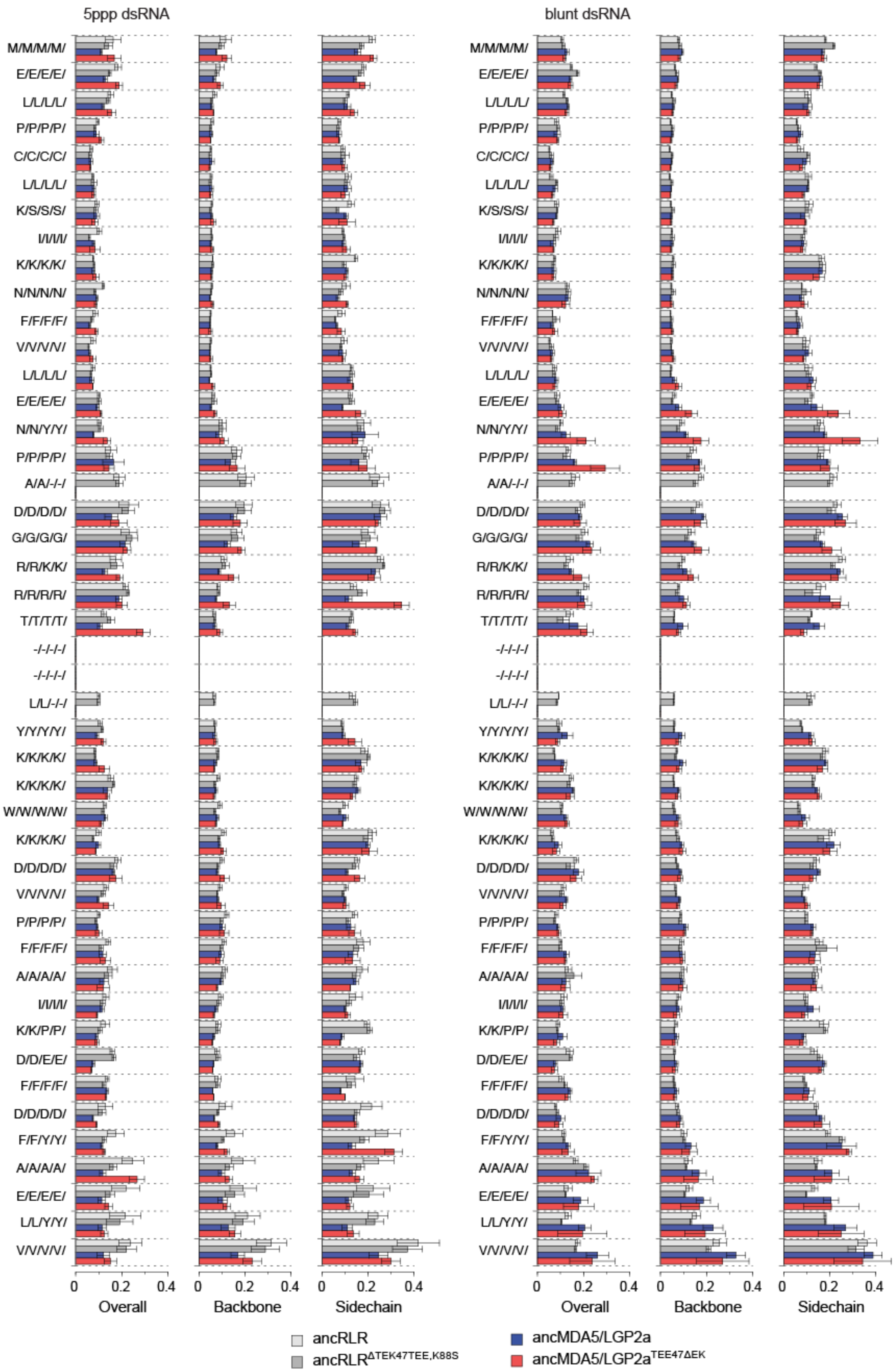
5ppp dsRNA
blunt dsRNA
M/M/M/M/
E/E/E/E/
L/L/L/L/
P/P/P/P/
C/C/C/C/
L/L/L/L/
K/S/S/S/
I/I/I/I/
K/K/K/K/
N/N/N/N/
F/F/F/F/
V/V/V/V/
L/L/L/L/
E/E/E/E/
N/N/Y/Y/
P/P/P/P/
A/A/-/-/
D/D/D/D/
G/G/G/G/
R/R/K/K/
R/R/R/R/
T/T/T/T/
-/-/-/-/
-/-/-/-/
L/L/-/-/
Y/Y/Y/Y/
K/K/K/K/
K/K/K/K/
W/W/W/W/
K/K/K/K/
D/D/D/D/
V/V/V/V/
P/P/P/P/
F/F/F/F/
A/A/A/A/
I/I/I/I/
K/K/P/P/
D/D/E/E/
F/F/F/F/
D/D/D/D/
F/F/Y/Y/
A/A/A/A/
E/E/E/E/
L/L/Y/Y/
V/V/V/V/
0
0.2
0.4
Overall
Backbone
Sidechain
ancRLR
ancRLR$^{\Delta TEK47TEE,K88S}$
ancMDA5/LGP2a
ancMDA5/LGP2a$^{TEE47\Delta EK}$

Figure S23. Root mean squared fluctuation (RMSF) differences between ancRLR and ancMDA5/LGP2a. We performed replicate molecular dynamics simulations of each RLR RD bound to blunt-ended and 5’ppp dsRNA (see Methods). We plot the overall, backbone and sidechain RMSFs of each residue, averaged over four dynamics simulations. Bars indicate standard errors.

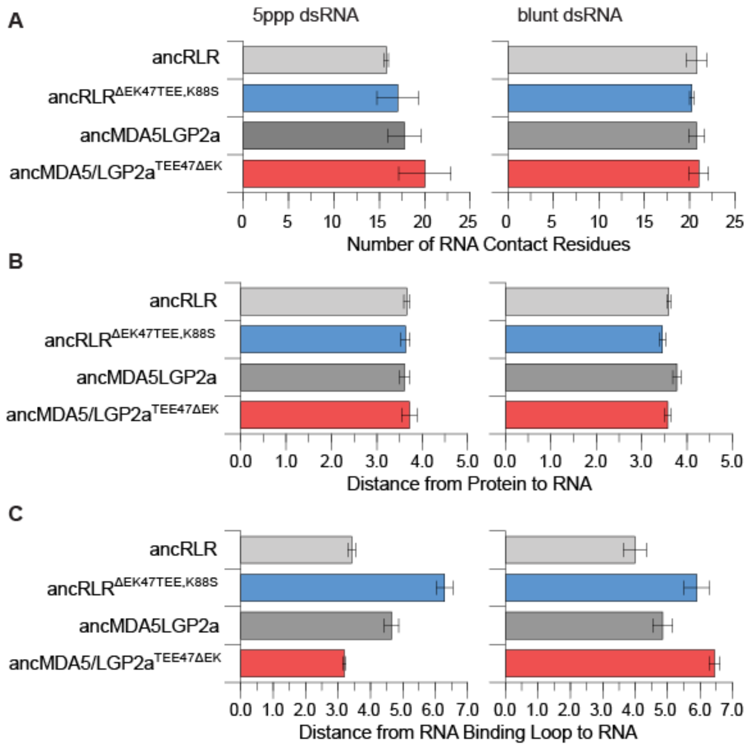

Figure S24. Reduced distance from the RNA binding loop to the 5’ppp dsRNA ligand correlates with greater affinity for 5’ppp dsRNA in ancRLR and ancMDA5/LGP2a RNA-recognition domains (RDs). We calculated the distance (in angstroms) between each residue and the RNA molecule over the course of replicate molecular dynamics simulations (see Methods). **A.** We plot the number of potential RNA contact residues, which we defined as residues having an average minimum distance to the RNA < 4 angstroms. **B.** We plot the average distance from each ancestral RD to the RNA molecule across all residues in the RD-RNA interface (average distance to RNA < 8 angstroms). **C.** We plot the average distance between all residues in the canonical RNA-binding loop (see Fig. 2) and the RNA molecule. Bars indicate standard errors over replicate dynamics simulations.

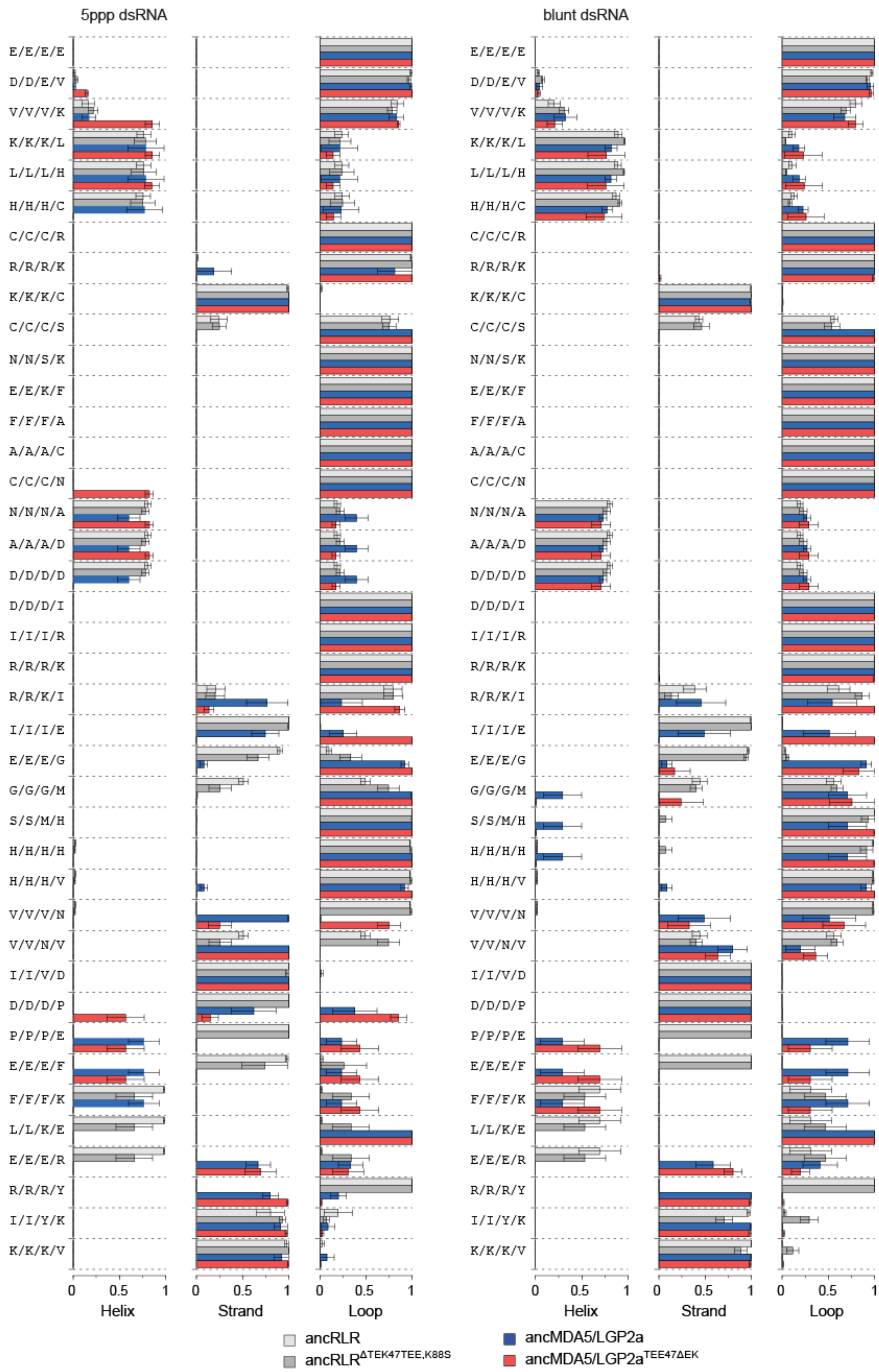

Figure S25, Part 1.

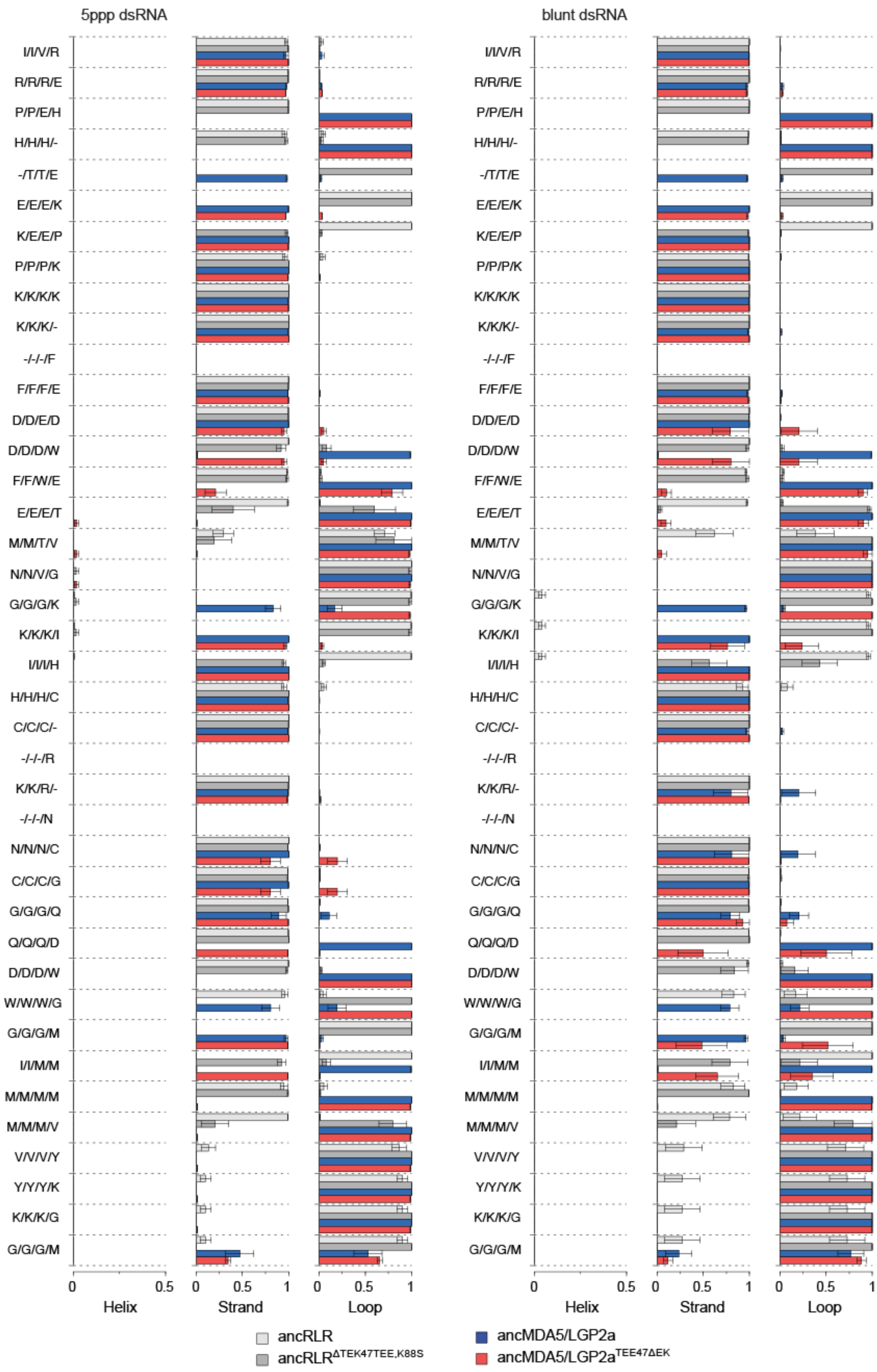

Figure S25, Part 2.

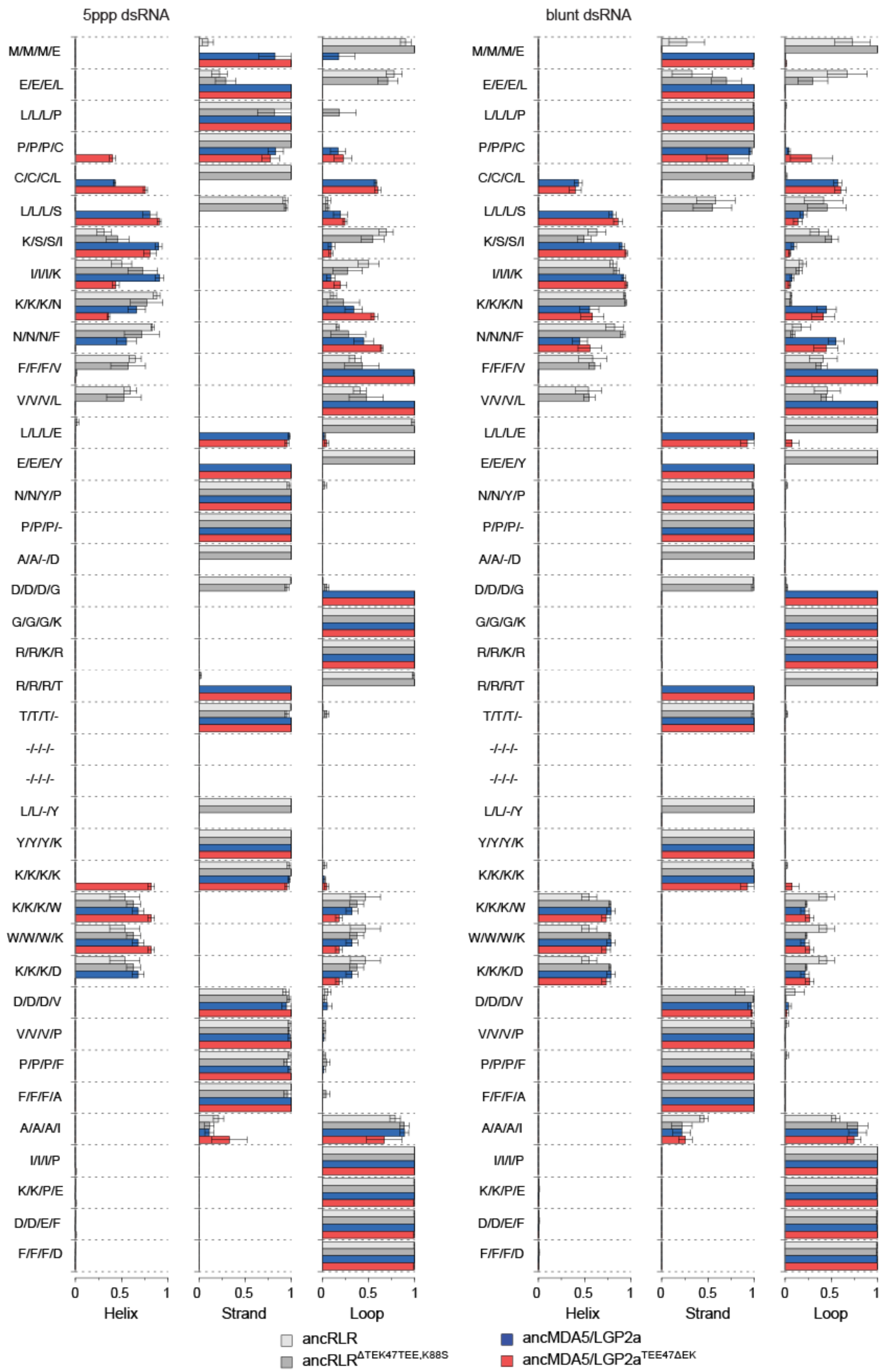

5ppp dsRNA
blunt dsRNA
M/M/M/E
E/E/E/L
L/L/L/P
P/P/P/C
C/C/C/L
L/L/L/S
K/S/S/I
I/I/I/K
K/K/K/N
N/N/N/F
F/F/F/V
V/V/V/L
L/L/L/E
E/E/E/Y
N/N/Y/P
P/P/P/-
A/A/-/D
D/D/D/G
G/G/G/K
R/R/K/R
R/R/R/T
T/T/T/-
-/-/-/-
-/-/-/-
L/L/-/Y
Y/Y/Y/K
K/K/K/K
K/K/K/W
W/W/W/K
K/K/K/D
D/D/D/V
V/V/V/P
P/P/P/F
F/F/F/A
A/A/A/I
I/I/I/P
K/K/P/E
D/D/E/F
F/F/F/D
0 0.5 1
Helix
Strand
Loop
ancRLR
ancRLR$^{\Delta TEK47TEE,K88S}$
ancMDA5/LGP2a
ancMDA5/LGP2a$^{TEE47\Delta EK}$

Figure S25. Protein secondary structure changes between ancRLR ancMDA5/LGP2a. We calculated protein secondary structure of ancRLR, ancMDA5/LGP2a and mutant RLR RDs across each molecular dynamics simulation (see Methods). For each residue, we plot the average proportion of molecular dynamics time points the residue was inferred as α-helix, β-strand or unstructured loop. Bars indicate standard errors over replicate dynamics simulations.

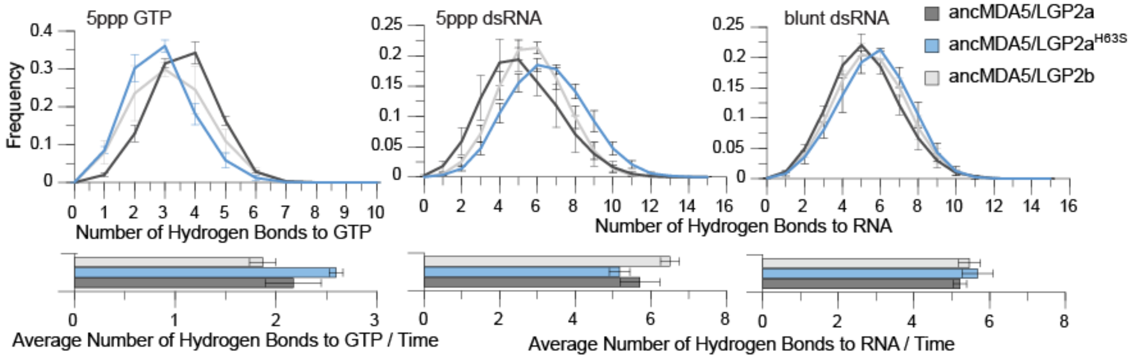

Figure S26. Hydrogen bonding between ancestral RNA recognition domains (RDs) and RNA ligands changes from ancMDA5/LGP2a to ancMDA5/LGP2b. We performed replicate molecular dynamics simulations of each ancestral or mutant RLR RD bound to blunt-ended or 5'ppp dsRNA (see Methods). Top panels plot the proportion of simulation samples from which we observed each number of hydrogen bonds between the RD and its RNA ligand, with bars indicating standard errors. Bottom panels plot the average and standard error of the number of hydrogen bonds between the RD and its RNA ligand over all sampled molecular dynamics time points.

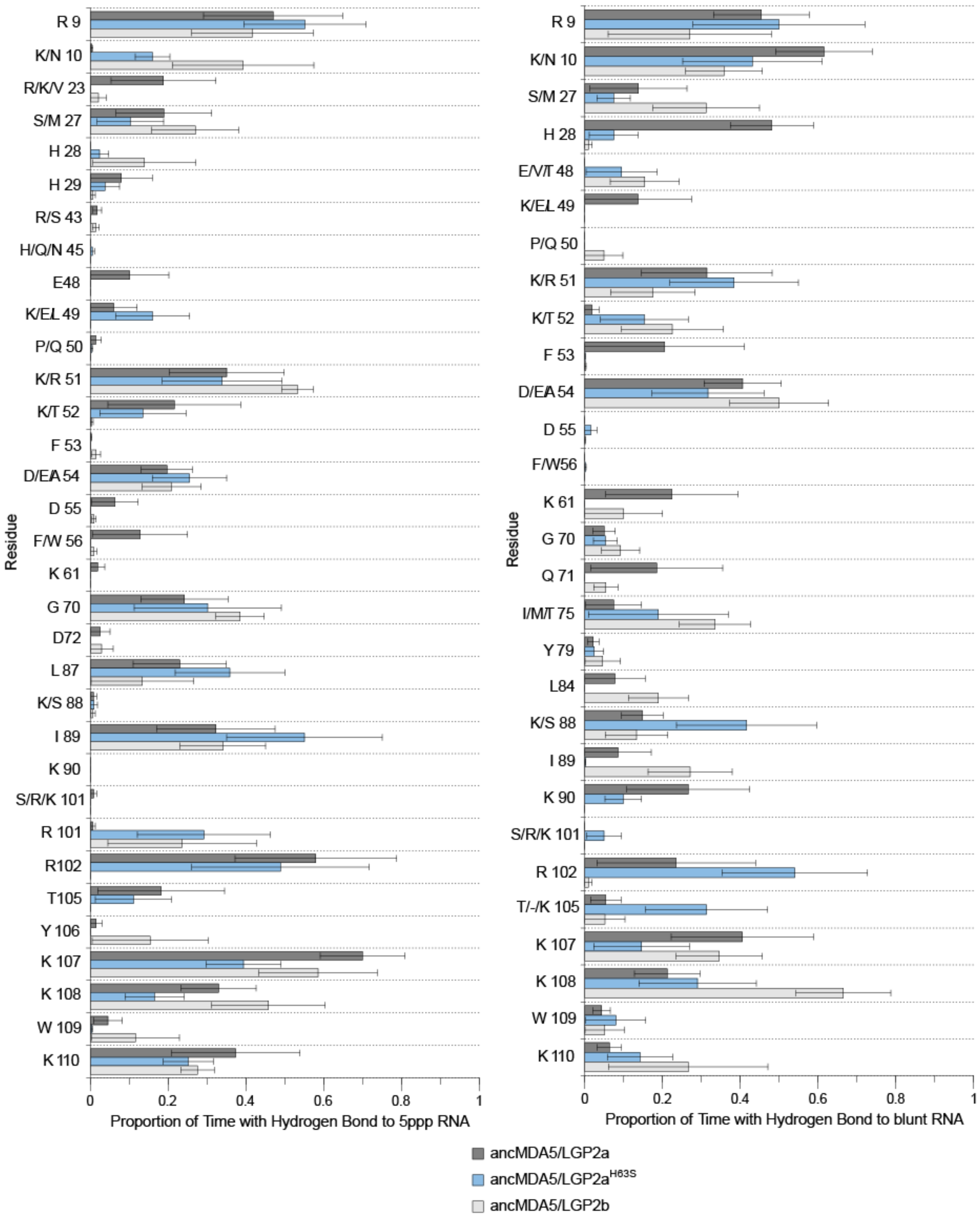

Figure S27. The capacity of specific residues to form hydrogen bonds to the RNA ligand changes between ancMDA5/LGP2a and ancMDA5/LGP2b. We performed replicate molecular dynamics simulations of each RLR RD bound to blunt-ended and 5'ppp dsRNA (see Methods). We plot the average proportion of time each residue formed hydrogen bonds with its RNA ligand over the course of molecular dynamics simulations. Bars indicate standard errors over four replicate dynamics simulations.

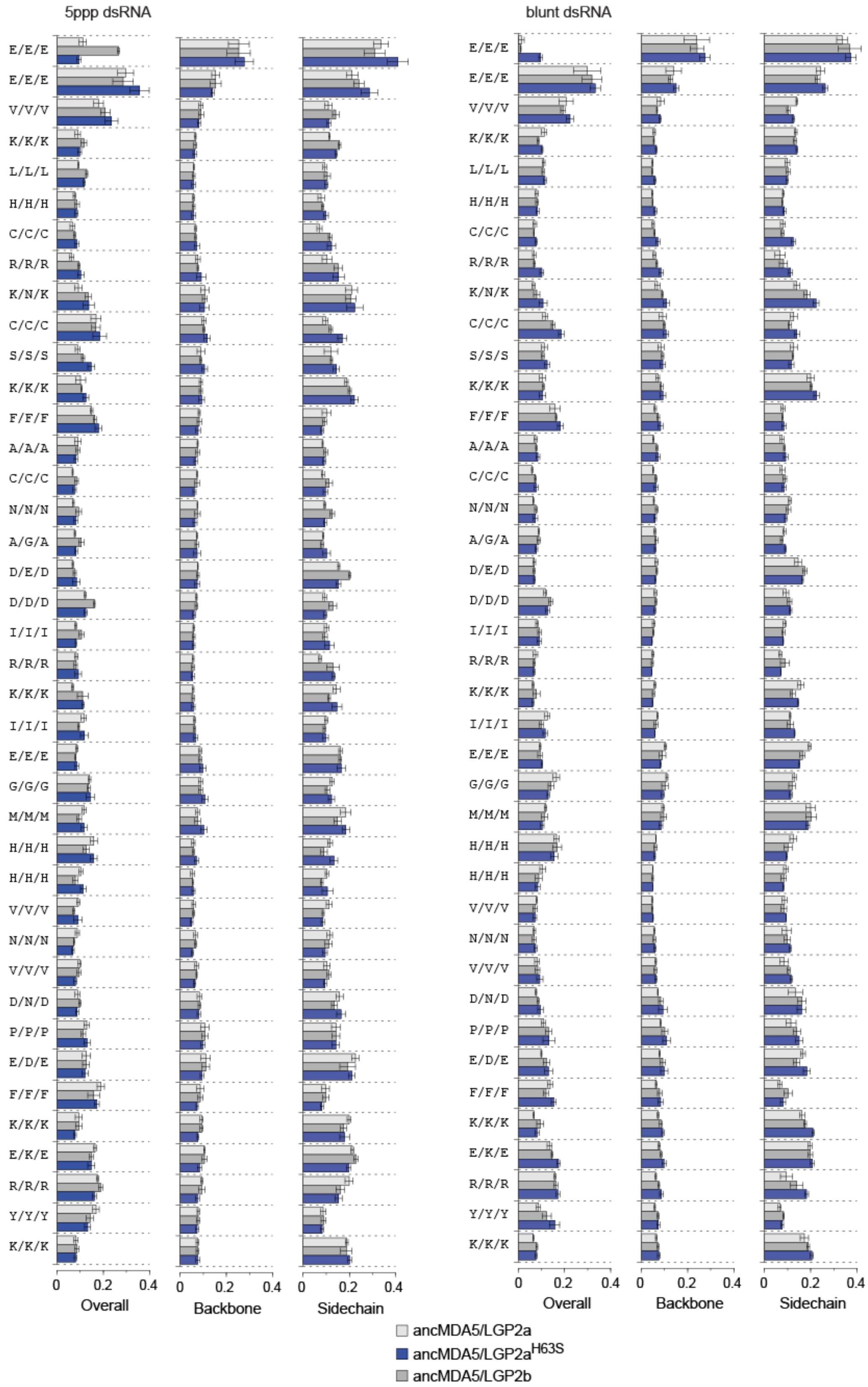

Figure S28, Part 1.

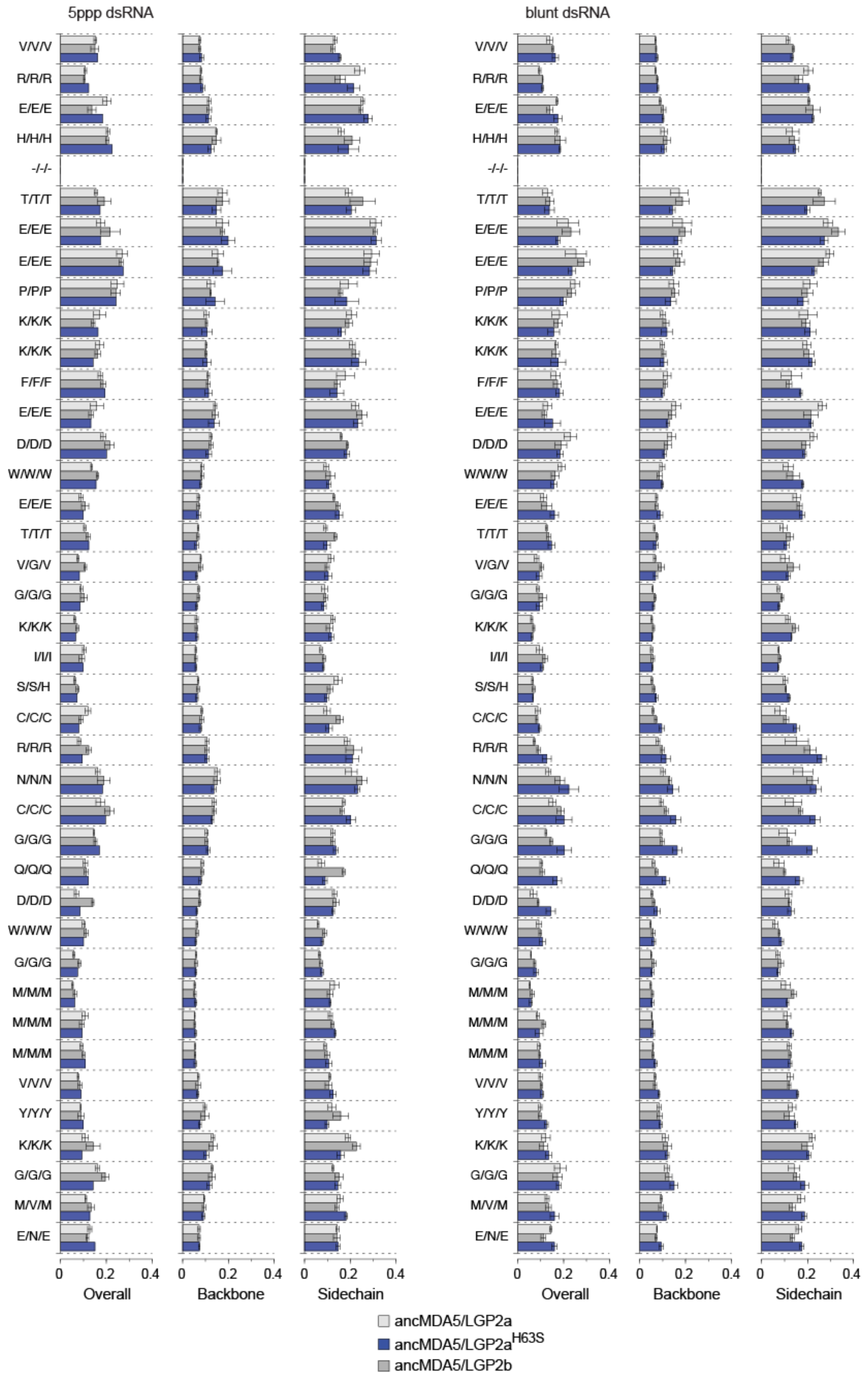

Figure S28, Part 2.

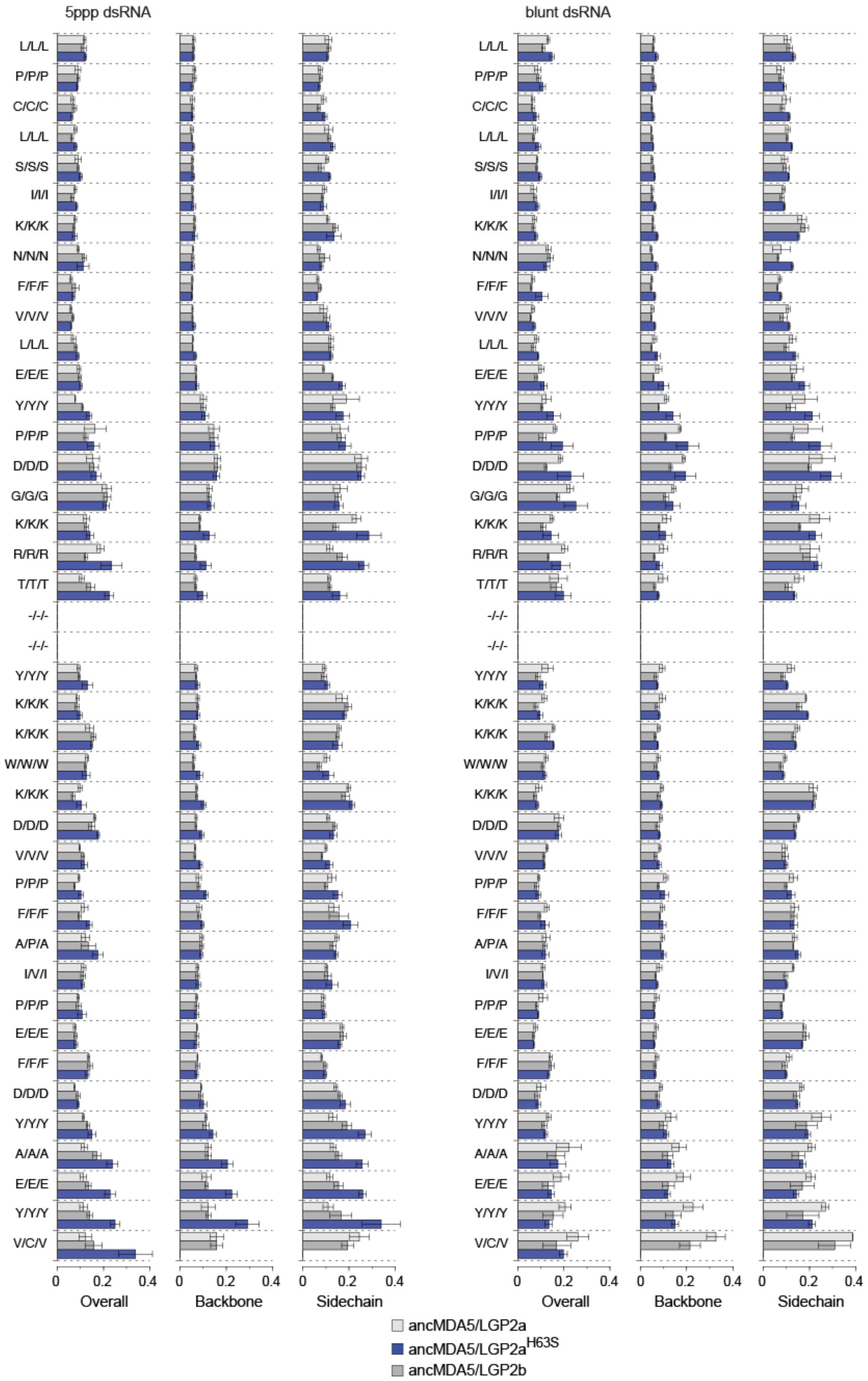

5ppp dsRNA
blunt dsRNA
L/L/L
P/P/P
C/C/C
L/L/L
S/S/S
I/I/I
K/K/K
N/N/N
F/F/F
V/V/V
L/L/L
E/E/E
Y/Y/Y
P/P/P
D/D/D
G/G/G
K/K/K
R/R/R
T/T/T
-/-/-
-/-/-
Y/Y/Y
K/K/K
K/K/K
W/W/W
K/K/K
D/D/D
V/V/V
P/P/P
F/F/F
A/P/A
I/V/I
P/P/P
E/E/E
F/F/F
D/D/D
Y/Y/Y
A/A/A
E/E/E
Y/Y/Y
V/C/V
0
0.2
0.4
Overall
Backbone
Sidechain
ancMDA5/LGP2a
ancMDA5/LGP2a^{H63S}
ancMDA5/LGP2b

Figure S28. Root mean squared fluctuation (RMSF) differences between ancMDA5/LGP2a and ancMDA5/LGP2b. We performed replicate molecular dynamics simulations of each RLR RD bound to blunt-ended and 5'ppp dsRNA (see Methods). We plot the overall, backbone and sidechain RMSFs of each residue, averaged over four dynamics simulations. Bars indicate standard errors.

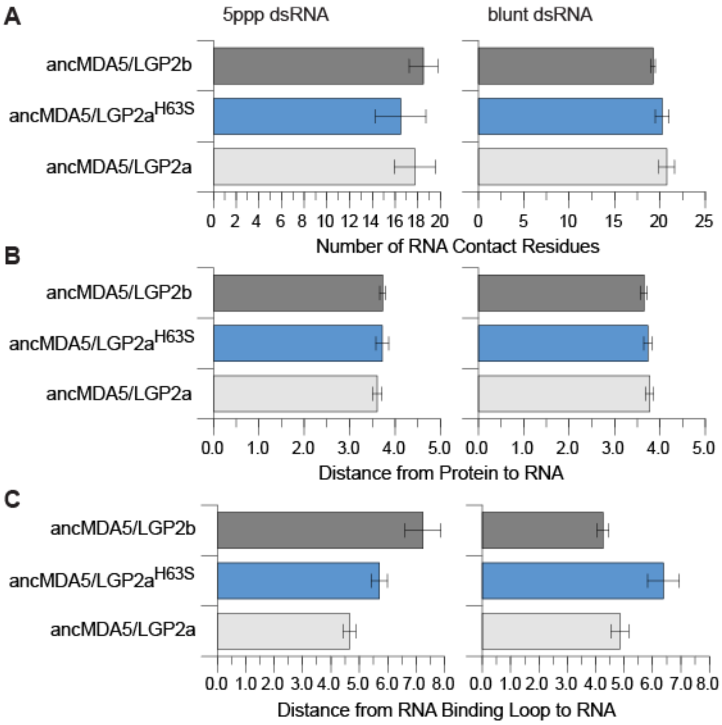

Figure S29. Reduced distance between the RD and its RNA ligand does not correlate with greater affinity for 5'ppp dsRNA in ancMDA5/LGP2a and ancMDA5/LGP2b RNA-recognition domains (RDs). We calculated the distance (in angstroms) between each residue and the RNA molecule over the course of replicate molecular dynamics simulations (see Methods). **A.** We plot the number of potential RNA contact residues, which we defined as residues having an average minimum distance to the RNA < 4 angstroms. **B.** We plot the average distance from each ancestral RD to the RNA molecule across all residues in the RD-RNA interface (average distance to RNA < 8 angstroms). **C.** We plot the average distance between all residues in the canonical RNA-binding loop (see Fig. 2) and the RNA molecule. Bars indicate standard errors over replicate dynamics simulations.

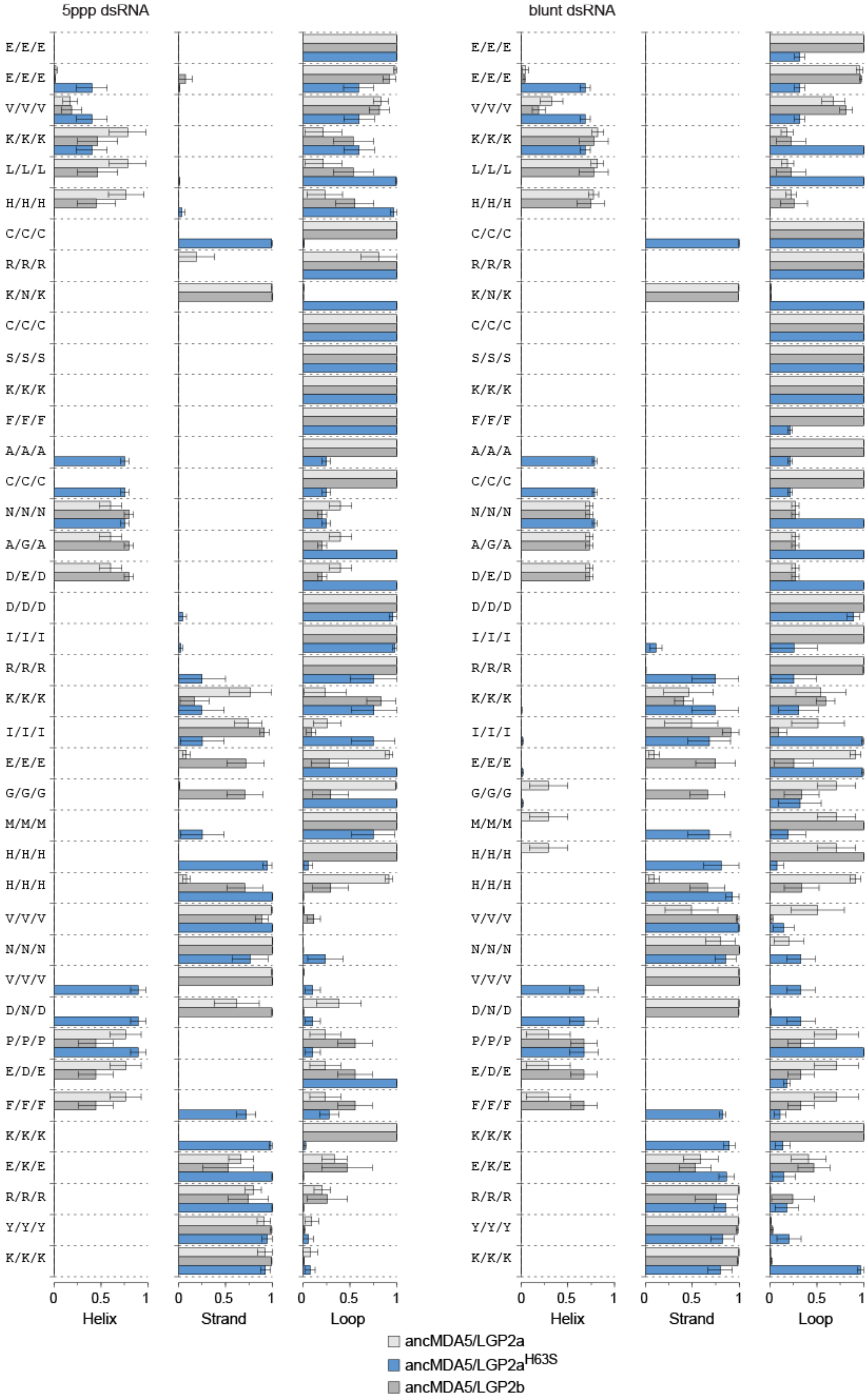

Figure S30, part 1.

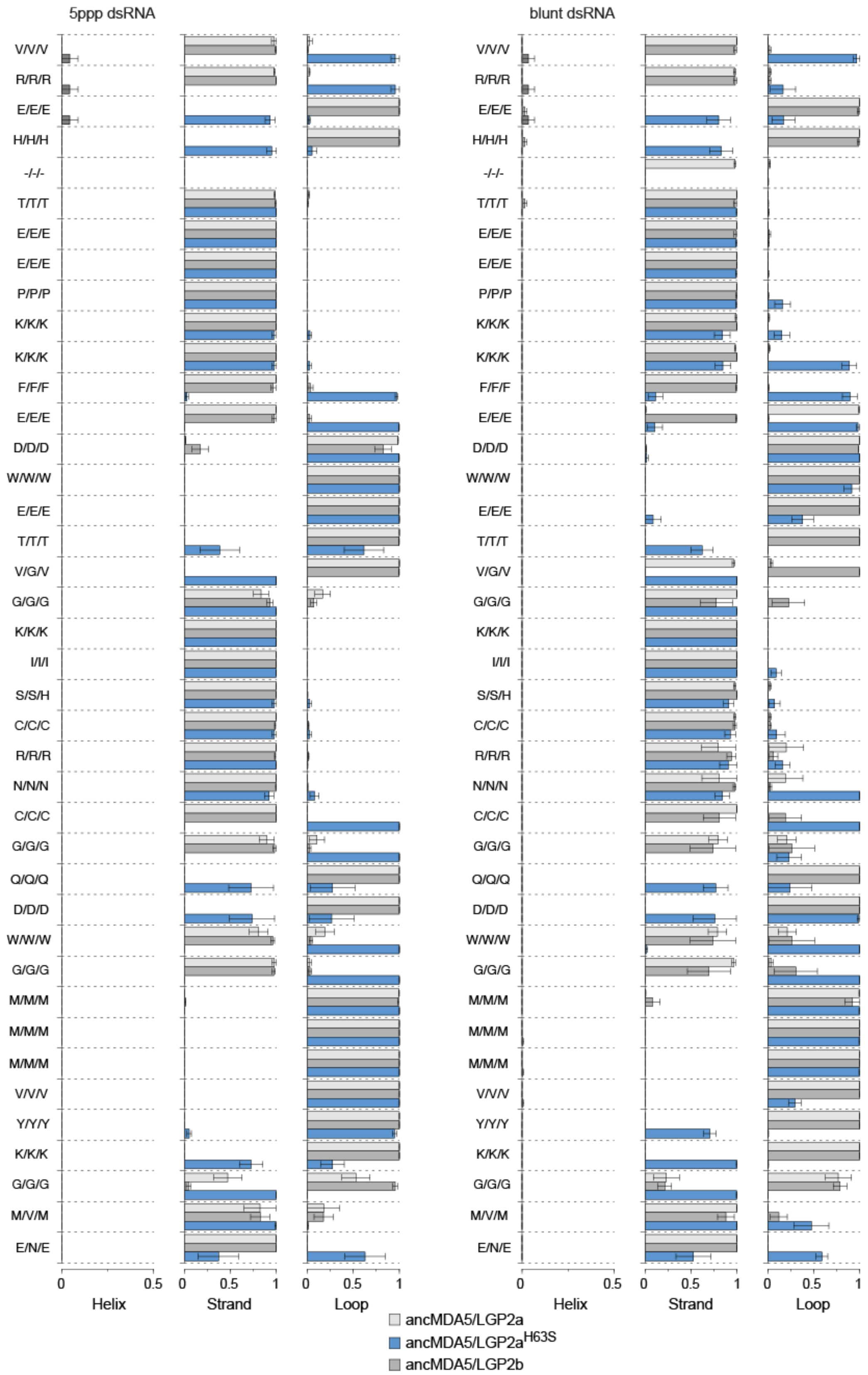

Figure S30, part 2.

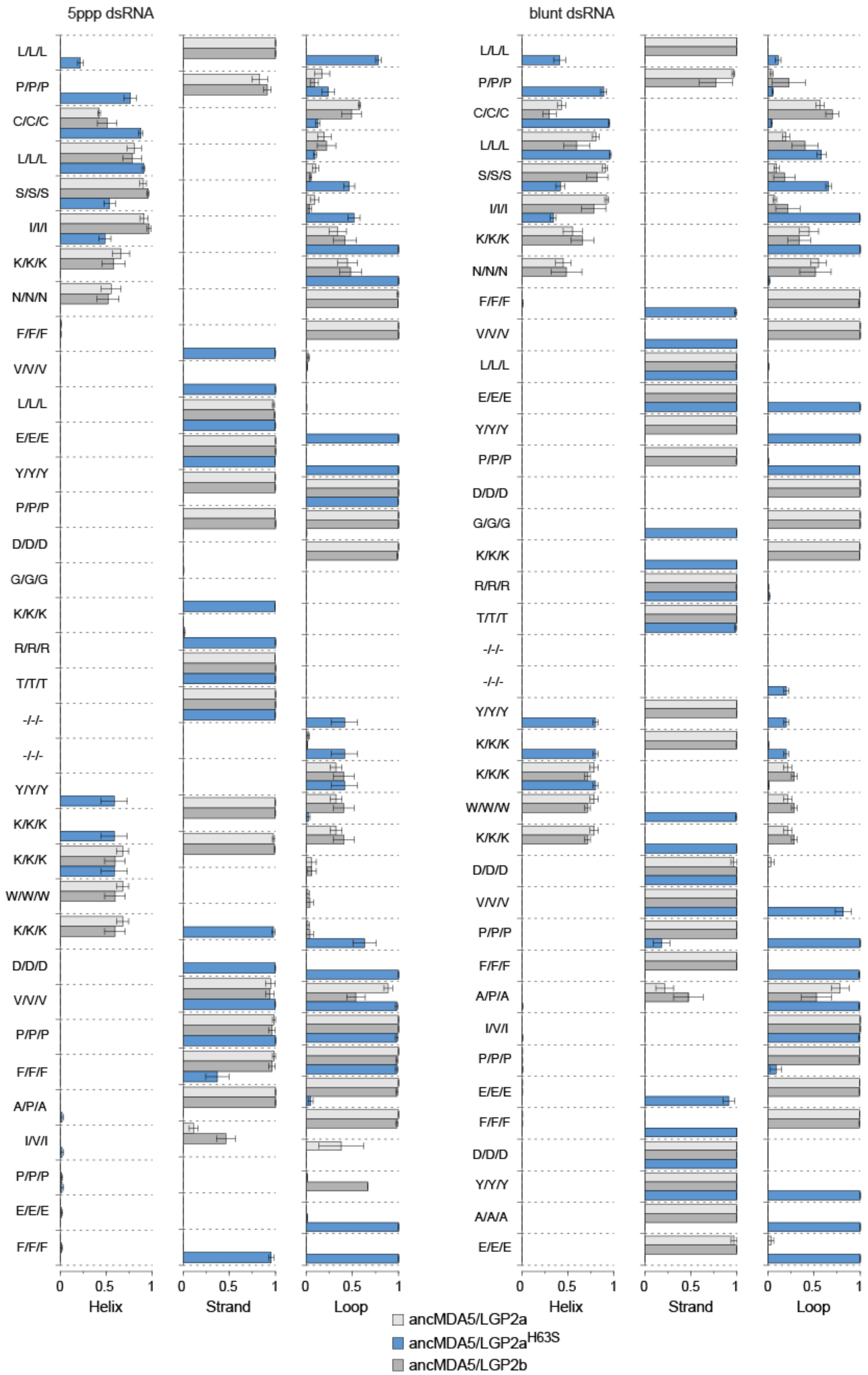

5ppp dsRNA
blunt dsRNA
L/L/L
P/P/P
C/C/C
L/L/L
S/S/S
I/I/I
K/K/K
N/N/N
F/F/F
V/V/V
L/L/L
E/E/E
Y/Y/Y
P/P/P
D/D/D
G/G/G
K/K/K
R/R/R
T/T/T
-/-/-
-/-/-
Y/Y/Y
K/K/K
K/K/K
W/W/W
K/K/K
D/D/D
V/V/V
P/P/P
F/F/F
A/P/A
I/V/I
P/P/P
E/E/E
F/F/F
A/A/A
0
0.5
1
Helix
Strand
Loop
ancMDA5/LGP2a
ancMDA5/LGP2a^{H63S}
ancMDA5/LGP2b

Figure S30. Protein secondary structure changes between ancMDA5/LGP2a ancMDA5/LGP2b. We calculated protein secondary structure of ancRLR, ancMDA5/LGP2a and mutant RLR RDs across each molecular dynamics simulation (see Methods). For each residue, we plot the average proportion of molecular dynamics time points the residue was inferred as α-helix, β-strand or unstructured loop. Bars indicate standard errors over replicate dynamics simulations.

Supplementary Tables

109115463 Macaca mulatta
111154107 Macaca mulatta
114052719 Macaca mulatta
114624048 Pan troglodytes
114624054 Pan troglodytes
114667313 Pan troglodytes
148231712 Xenopus laevis
149408122 Homo sapiens
149408137 Rattus norvegicus
149730873 Equus caballus
153945886 Mus musculus
154147577 Sus scrofa
156393350 Nematostella vectensis
156393452 Nematostella vectensis
156401322 Nematostella vectensis
157822253 Rattus norvegicus
170593727 Brugia malayi
17539846 Caenorhabditis elegans
194224905 Equus caballus
197098648 Pongo abelii
198430581 Ciona intestinalis
213513760 Salmo salar
254911056 Salmo salar
257096036 Mus musculus
257096038 Mus musculus
260809266 Branchiostoma floridae
260809268 Branchiostoma floridae
260809270 Branchiostoma floridae
260817577 Branchiostoma floridae
268536792 Caenorhabditis briggsae
268536796 Caenorhabditis briggsae
268567267 Caenorhabditis briggsae
27881482 Homo sapiens
27886568 Homo sapiens
291235953 Saccoglossus kowalevskii
291383111 Oryctolagus cuniculus
291391627 Oryctolagus cuniculus
291406113 Oryctolagus cuniculus
296190081 Callithrix jacchus
296202916 Callithrix jacchus
296202918 Callithrix jacchus
296204692 Callithrix jacchus
297273051 Macaca mulatta
297471684 Bos taurus
297668722 Pongo abelii
297684253 Pongo abelii
301773535 Ailuropoda melanoleuca
301778989 Ailuropoda melanoleuca
301785794 Ailuropoda melanoleuca
302370918 Gallus gallus
304434770 Oncorhynchus mykiss
308455471 Caenorhabditis remanei
308466625 Caenorhabditis remanei
308469221 Caenorhabditis remanei
308469293 Caenorhabditis remanei
312069940 Loa loa
312079013 Loa loa
312284067 Sus scrofa
326922819 Meleagris gallopavo
327272183 Anolis carolinensis
327283103 Anolis carolinensis
332234337 Nomascus leucogenys
332814684 Pan troglodytes
339247851 Trichinella spiralis
339259444 Trichinella spiralis
340369687 Amphimedon queenslandica
344268047 Loxodonta africana
344271077 Loxodonta africana
345308281 Ornithorhynchus anatinus
348562460 Cavia porcellus
348569861 Cavia porcellus
348585927 Cavia porcellus
354485032 Cricetulus griseus
354493094 Cricetulus griseus
356460981 Sus scrofa
359320815 Canis lupus familiaris
363743602 Gallus gallus
380422390 Danio rerio
390331517 Strongylocentrotus purpuratus
390331519 Strongylocentrotus purpuratus
390331521 Strongylocentrotus purpuratus
390331523 Strongylocentrotus purpuratus
390336956 Strongylocentrotus purpuratus
390341546 Strongylocentrotus purpuratus
390342381 Strongylocentrotus purpuratus
390347586 Strongylocentrotus purpuratus
390354545 Strongylocentrotus purpuratus
390463706 Callithrix jacchus
395519645 Sarcophilus harrisii
395532380 Sarcophilus harrisii
395826380 Otolemur garnettii
395826382 Otolemur garnettii
395826384 Otolemur garnettii
395844909 Otolemur garnettii
395855808 Otolemur garnettii
395855810 Otolemur garnettii
397485570 Pan paniscus
397485572 Pan paniscus
397500570 Pan paniscus
397520025 Pan paniscus
397520027 Pan paniscus
402888490 Papio anubis
402897212 Papio anubis
402900290 Papio anubis
403258897 Saimiri boliviensis boliviensis
403297888 Saimiri boliviensis boliviensis

403304470 Saimiri boliviensis boliviensis
403304472 Saimiri boliviensis boliviensis
410897044 Takifugu rubripes
410902733 Takifugu rubripes
426221009 Ovis aries
426222380 Ovis aries
426238043 Ovis aries
426337537 Gorilla gorilla gorilla
426348251 Gorilla gorilla gorilla
426361539 Gorilla gorilla gorilla
432867922 Oryzias latipes
432934592 Oryzias latipes
441677777 Nomascus leucogenys
449491219 Taeniopygia guttata
449507056 Taeniopygia guttata
449514052 Taeniopygia guttata
459185383 Ciona intestinalis
466035138 Orcinus orca
466073108 Orcinus orca
466077086 Orcinus orca
470628436 Tursiops truncatus
470640590 Tursiops truncatus
471369945 Trichechus manatus latirostris
471417714 Trichechus manatus latirostris
471417716 Trichechus manatus latirostris
471418994 Trichechus manatus latirostris
472344976 Odobenus rosmarus divergens
472381721 Odobenus rosmarus divergens
472382387 Odobenus rosmarus divergens
478508066 Ceratotherium simum simum
478508301 Ceratotherium simum simum
478508303 Ceratotherium simum simum
478520621 Ceratotherium simum simum
488545868 Dasypus novemcinctus
488545870 Dasypus novemcinctus
488545872 Dasypus novemcinctus
488565638 Dasypus novemcinctus
488565640 Dasypus novemcinctus
488594425 Dasypus novemcinctus
498991746 Maylandia zebra
499012355 Maylandia zebra
499013353 Maylandia zebra
504127371 Ochotona princeps
504137312 Ochotona princeps
504181289 Ochotona princeps
505768383 Sorex araneus
505800717 Sorex araneus
505803065 Sorex araneus
507535823 Jaculus jaculus
507535825 Jaculus jaculus
507542883 Jaculus jaculus
507549246 Jaculus jaculus
507639468 Octodon degus
507648173 Octodon degus
507650869 Echinops telfairi
507655253 Octodon degus
507665028 Echinops telfairi
507688690 Echinops telfairi
507926800 Condylura cristata
507952430 Condylura cristata
507954670 Condylura cristata
511838976 Mustela putorius furo
511891446 Mustela putorius furo
511891448 Mustela putorius furo
511891450 Mustela putorius furo
511891452 Mustela putorius furo
511891454 Mustela putorius furo
511906606 Mustela putorius furo
511906608 Mustela putorius furo
511945175 Mustela putorius furo
511971782 Mustela putorius furo
511971784 Mustela putorius furo
511971786 Mustela putorius furo
511971788 Mustela putorius furo
511971790 Mustela putorius furo
512015167 Mustela putorius furo
512015169 Mustela putorius furo
512808626 Heterocephalus glaber
512808628 Heterocephalus glaber
512808630 Heterocephalus glaber
512813647 Xenopus (Silurana) tropicalis
512840572 Heterocephalus glaber
512865974 Xenopus (Silurana) tropicalis
512874269 Xenopus (Silurana) tropicalis
512874273 Xenopus (Silurana) tropicalis
512874277 Xenopus (Silurana) tropicalis
512924800 Heterocephalus glaber
512946853 Heterocephalus glaber
512946855 Heterocephalus glaber
512946857 Heterocephalus glaber
512952063 Heterocephalus glaber
512995649 Heterocephalus glaber
513226256 Gallus gallus
513226259 Gallus gallus
513226262 Gallus gallus
514455710 Cavia porcellus
514750528 Anas platyrhynchos
514776939 Anas platyrhynchos
514796163 Anas platyrhynchos
523704380 Ictalurus punctatus
523704386 Ictalurus punctatus
523704392 Ictalurus punctatus
524866761 Aplysia californica
524866763 Aplysia californica
524892613 Aplysia californica
524895767 Aplysia californica
524958007 Mesocricetus auratus
524975237 Mesocricetus auratus
525003555 Ficedula albicollis
525024579 Ficedula albicollis
525027443 Ficedula albicollis
527246408 Melopsittacus undulatus
527247366 Melopsittacus undulatus
527269403 Melopsittacus undulatus
528491003 Danio rerio
528908090 Bos taurus
528908092 Bos taurus
528958082 Bos taurus
528958084 Bos taurus
529425898 Falco peregrinus
529429213 Falco peregrinus
529430101 Falco peregrinus
530604799 Chrysemys picta bellii
530604801 Chrysemys picta bellii

530604803 Chrysemys picta bellii
530604805 Chrysemys picta bellii
530604807 Chrysemys picta bellii
530606212 Chrysemys picta bellii
530606214 Chrysemys picta bellii
532005375 Microtus ochrogaster
532005377 Microtus ochrogaster
532039126 Microtus ochrogaster
532052660 Microtus ochrogaster
532059804 Ictidomys tridecemlineatus
532059806 Ictidomys tridecemlineatus
532059808 Ictidomys tridecemlineatus
532072212 Ictidomys tridecemlineatus
532080950 Ictidomys tridecemlineatus
533158924 Chinchilla lanigera
533158926 Chinchilla lanigera
533161308 Chinchilla lanigera
533161310 Chinchilla lanigera
533175819 Chinchilla lanigera
541953364 Falco cherrug
541971298 Falco cherrug
541981722 Falco cherrug
542152859 Zonotrichia albicollis
542168361 Zonotrichia albicollis
542174161 Zonotrichia albicollis
542181785 Oreochromis niloticus
542181789 Oreochromis niloticus
542228550 Oreochromis niloticus
543245956 Geospiza fortis
543271683 Geospiza fortis
543291626 Geospiza fortis
543348865 Pseudopodoces humilis
543373588 Pseudopodoces humilis
543376777 Pseudopodoces humilis
543714794 Columba livia
543739380 Columba livia
543745715 Columba livia
544475442 Macaca fascicularis
544493999 Macaca fascicularis
544494001 Macaca fascicularis
544494003 Macaca fascicularis
544500072 Macaca fascicularis
544500074 Macaca fascicularis
544500076 Macaca fascicularis
545180520 Equus caballus
545201667 Equus caballus
545510597 Canis lupus familiaris
545517283 Canis lupus familiaris
545857927 Sus scrofa
545857930 Sus scrofa
545874461 Sus scrofa
548341928 Pundamilia nyererei
548347500 Pundamilia nyererei
548347502 Pundamilia nyererei
548347926 Pundamilia nyererei
548453393 Capra hircus
548476237 Capra hircus
548509077 Capra hircus
548509079 Capra hircus
551490362 Xiphophorus maculatus
551507740 Xiphophorus maculatus
554529333 Myotis brandtii
554539925 Myotis brandtii
554562932 Myotis brandtii
554562934 Myotis brandtii
554814916 Haplochromis burtoni
554814918 Haplochromis burtoni
554819328 Haplochromis burtoni
554819330 Haplochromis burtoni
554819332 Haplochromis burtoni
554819334 Haplochromis burtoni
555970659 Bos mutus
555974569 Bos mutus
555994926 Bos mutus
556736769 Pantholops hodgsonii
556738700 Pantholops hodgsonii
556745786 Pantholops hodgsonii
556987117 Latimeria chalumnae
557005377 Latimeria chalumnae
557018441 Latimeria chalumnae
557284378 Alligator sinensis
557319289 Alligator sinensis
557329201 Alligator sinensis
558101469 Myotis lucifugus
558101471 Myotis lucifugus
558124456 Pelodiscus sinensis
558141560 Myotis lucifugus
558141564 Myotis lucifugus
558162372 Pelodiscus sinensis
558162376 Pelodiscus sinensis
558170072 Myotis lucifugus
558198829 Pelodiscus sinensis
560914247 Camelus ferus
560927981 Camelus ferus
560932894 Camelus ferus
560932896 Camelus ferus
560932898 Camelus ferus
560947414 Vicugna pacos
560953594 Vicugna pacos
560989496 Vicugna pacos
562854944 Tupaia chinensis
562864169 Tupaia chinensis
564242519 Alligator mississippiensis
564251110 Alligator mississippiensis
564253027 Alligator mississippiensis
564341167 Rattus norvegicus
564374222 Rattus norvegicus
564374224 Rattus norvegicus
564374226 Rattus norvegicus
568918427 Mus musculus
573877187 Lepisosteus oculatus
573883999 Lepisosteus oculatus
573897889 Lepisosteus oculatus
573901160 Lepisosteus oculatus
583986710 Neolamprologus brichardi
584006652 Neolamprologus brichardi
584006654 Neolamprologus brichardi
584043505 Myotis davidii
584064706 Myotis davidii
584077036 Myotis davidii
585149822 Leptonychotes weddellii
585173429 Leptonychotes weddellii
585173431 Leptonychotes weddellii
585191864 Leptonychotes weddellii

585638395 Elephantulus edwardii
585642658 Elephantulus edwardii
585681904 Saccoglossus kowalevskii
585690235 Saccoglossus kowalevskii
585717270 Elephantulus edwardii
586449065 Chrysochloris asiatica
586472956 Chrysochloris asiatica
586539674 Pteropus alecto
586539676 Pteropus alecto
586565816 Pteropus alecto
586997260 Felis catus
587008394 Felis catus
587008396 Felis catus
587011657 Felis catus
588480439 Pteropus alecto
588480490 Pteropus alecto
588480506 Pteropus alecto
589925029 Peromyscus maniculatus bairdii
589943938 Peromyscus maniculatus bairdii
589960757 Peromyscus maniculatus bairdii
591299240 Panthera tigris altaica
591299242 Panthera tigris altaica
591303338 Panthera tigris altaica
591303340 Panthera tigris altaica
591323280 Panthera tigris altaica
591359695 Chelonia mydas
591363601 Chelonia mydas
591381440 Chelonia mydas
593717924 Physeter catodon
593747658 Physeter catodon
593755350 Physeter catodon
594063588 Bubalus bubalis
594073010 Bubalus bubalis
594101952 Bubalus bubalis
594657107 Balaenoptera acutorostrata scammoni
594658856 Balaenoptera acutorostrata scammoni
594658858 Balaenoptera acutorostrata scammoni
594658860 Balaenoptera acutorostrata scammoni
594659124 Balaenoptera acutorostrata scammoni
594659126 Balaenoptera acutorostrata scammoni
594659128 Balaenoptera acutorostrata scammoni
594670557 Balaenoptera acutorostrata scammoni
594670559 Balaenoptera acutorostrata scammoni
594670561 Balaenoptera acutorostrata scammoni
594670563 Balaenoptera acutorostrata scammoni
597736428 Astyanax mexicanus
597736430 Astyanax mexicanus
597751074 Astyanax mexicanus
597785802 Astyanax mexicanus
602629316 Python bivittatus
602655369 Python bivittatus
602677894 Python bivittatus
602677896 Python bivittatus
602705673 Lipotes vexillifer
602708581 Lipotes vexillifer
602709522 Lipotes vexillifer
611991734 Monodelphis domestica
611991736 Monodelphis domestica
612019329 Monodelphis domestica
612019331 Monodelphis domestica
617381433 Poecilia formosa
617420008 Poecilia formosa
617605689 Erinaceus europaeus
617638697 Erinaceus europaeus
617654303 Erinaceus europaeus
620978842 Ornithorhynchus anatinus
625206893 Cricetulus griseus
625213678 Cricetulus griseus
625234420 Cricetulus griseus
625234422 Cricetulus griseus
625246515 Cricetulus griseus
625254256 Cricetulus griseus
62751389 Bos taurus
632945385 Callorhinchus milii
632976448 Callorhinchus milii
632981623 Callorhinchus milii
634850933 Orycteropus afer afer
634851763 Orycteropus afer afer
634875734 Orycteropus afer afer
635058327 Chlorocebus sabaeus
635058329 Chlorocebus sabaeus
635073168 Chlorocebus sabaeus
635092341 Chlorocebus sabaeus
635092343 Chlorocebus sabaeus
635092345 Chlorocebus sabaeus
635092347 Chlorocebus sabaeus
635092349 Chlorocebus sabaeus
635092351 Chlorocebus sabaeus
637299422 Anolis carolinensis
637299426 Anolis carolinensis
637299430 Anolis carolinensis
637299434 Anolis carolinensis
637299444 Anolis carolinensis
637299448 Anolis carolinensis
637344897 Anolis carolinensis
637352188 Anolis carolinensis
640780400 Tarsius syrichta
640792670 Tarsius syrichta
640805855 Tarsius syrichta
641696885 Eptesicus fuscus
641703041 Eptesicus fuscus
641703043 Eptesicus fuscus
641716301 Eptesicus fuscus
641763351 Chrysemys picta bellii
641789818 Chrysemys picta bellii
655600047 Oryctolagus cuniculus
655600049 Oryctolagus cuniculus
657557925 Stegastes partitus
657566562 Stegastes partitus
657799628 Cynoglossus semilaevis
657800674 Cynoglossus semilaevis
658839718 Poecilia reticulata
658864888 Poecilia reticulata
663239641 Calypte anna
663247831 Calypte anna
663268328 Calypte anna
70608133 Mus musculus
71982860 Caenorhabditis elegans
72010927 Strongylocentrotus purpuratus
74004732 Canis lupus familiaris
76609700 Bos taurus

Table S1. Genbank protein IDs for all sequences used in this study.

5’ppp **Best Models**

	ancRLR		ancRIG-I		ancMDA5-LGP2a		ancMDA5-LGP2b		ancMDA5		ancLGP2	
Scoring function term	Raw score	Z-score	Raw score	Z-score	Raw score	Z-score	Raw score	Z-score	Raw score	Z-score	Raw score	Z-score
C_beta interaction energy	-87.66	-0.12	-91.71	-0.05	-57.64	-0.93	-29.72	-1.69	-73.95	-0.47	-57.59	-0.96
All-atom pairwise energy	-4577.51	-0.93	-4652.04	-0.92	-4404.85	-1.01	-2200.91	-1.66	-4343.1	-1.01	-4343.63	-1.11
Solvation energy	-26.25	-0.11	-27.11	-0.02	-31.28	0.47	-14.77	0.66	-23.96	-0.43	-8.07	-2.23
Torsion angle energy	-68.37	0.07	-56.63	-0.53	-38.4	-1.43	-12.95	-2.02	-35.57	-1.62	-32.33	-1.83
Secondary structure agreement	67.90%	-2.21	60.80%	-3.27	65.10%	-2.6	63.00%	-2.6	68.30%	-2.2	69.80%	-2.07
Solvent accessibility agreement	79.20%	0.03	80.00%	0.19	84.00%	0.96	80.70%	0.9	78.50%	-0.09	78.70%	-0.08
QMEAN6 score	0.686	-0.77	0.647	-1.19	0.682	-0.8	0.602	-1.45	0.614	-1.52	0.637	-1.34
dfire energy	-280.44	N/A	-278.59	N/A	-266.28	N/A	-132.88	N/A	-282.76	N/A	-284.48	N/A

5’ppp **Central Structures**

	ancRLR		ancRIG-I		ancMDA5-LGP2a		ancMDA5-LGP2b		ancMDA5		ancLGP2	
Scoring function term	Raw score	Z-score	Raw score	Z-score	Raw score	Z-score	Raw score	Z-score	Raw score	Z-score	Raw score	Z-score
C_beta interaction energy	-51.56	-0.84	-63.55	-0.48	-25.59	-1.78	-53.19	-0.81	-48.92	-0.98	-29	-1.6
All-atom pairwise energy	-4270.02	-0.55	-5153.45	-0.09	-3669.48	-1.01	-3748.56	-0.93	-3892.97	-0.82	-4270.88	-0.65
Solvation energy	-23.11	-0.2	-28.59	0.42	-21.8	-0.41	-20.06	-0.6	-2.39	-2.56	-3.8	-2.62
Torsion angle energy	-37.81	-0.45	-43.38	-0.02	-23.5	-1.84	-47.85	-0.48	-34.58	-1.11	-26.88	-1.74
Secondary structure agreement	60.90%	-3.13	59.50%	-3.33	59.70%	-3.35	56.10%	-3.8	69.60%	-1.89	63.10%	-2.98
Solvent accessibility agreement	80.00%	0.18	79.10%	0.02	80.50%	0.31	81.10%	0.42	79.60%	0.15	81.60%	0.48
QMEAN6 score	0.66	-1.01	0.656	-1.06	0.604	-1.59	0.657	-1.03	0.692	-0.67	0.672	-0.92
dfire energy	-297.73	N/A	-305.79	N/A	-284.06	N/A	-274.57	N/A	-291.09	N/A	-292.82	N/A

Blunt **Best Models**

	ancRLR		ancRIG-I		ancMDA5-LGP2a		ancMDA5-LGP2b		ancMDA5		ancLGP2	
Scoring function term	Raw score	Z-score	Raw score	Z-score	Raw score	Z-score	Raw score	Z-score	Raw score	Z-score	Raw score	Z-score
C_beta interaction energy	-86.67	-0.12	-84.67	-0.21	-66.03	-0.68	-27.53	-1.77	-74.84	-0.42	-71.8	-0.54
All-atom pairwise energy	-4849.47	-0.77	-5062.41	-0.69	-4548.76	-0.93	-2398.1	-1.47	-5102.42	-0.57	-4897.89	-0.8
Solvation energy	-25.41	-0.2	-23.41	-0.42	-33.33	0.69	-14.59	0.64	-20.29	-0.83	-18.46	-1.13
Torsion angle energy	-51.7	-0.78	-60.36	-0.34	-40.55	-1.32	-23.01	-1.05	-30.67	-1.86	-43.36	-1.31
Secondary structure agreement	68.80%	-2.09	63.30%	-2.89	66.80%	-2.35	63.90%	-2.49	67.90%	-2.26	67.80%	-2.37
Solvent accessibility agreement	79.60%	0.11	80.00%	0.19	79.00%	0.02	79.00%	0.58	80.10%	0.21	82.60%	0.64
QMEAN6 score	0.667	-0.97	0.673	-0.91	0.615	-1.51	0.623	-1.22	0.64	-1.25	0.697	-0.67
dfire energy	-276.06	N/A	-276.37	N/A	-267.83	N/A	-135.94	N/A	-283.73	N/A	-287.44	N/A

Blunt **Central Structures**

	ancRLR		ancRIG-I		ancMDA5-LGP2a		ancMDA5-LGP2b		ancMDA5		ancLGP2	
Scoring function term	Raw score	Z-score	Raw score	Z-score	Raw score	Z-score	Raw score	Z-score	Raw score	Z-score	Raw score	Z-score
C_beta interaction energy	-47.65	-0.98	-46.73	-1	-32.62	-1.56	-24.36	-1.82	-45.68	-1.08	-27.19	-1.72
All-atom pairwise energy	-4628.6	-0.33	-4342.24	-0.47	-4148.26	-0.76	-3628.43	-1.05	-4346.2	-0.54	-4063.86	-0.91
Solvation energy	-19.63	-0.59	-23.74	-0.12	-19.32	-0.69	-22.37	-0.35	-8.24	-1.93	-7.54	-2.21
Torsion angle energy	-41.52	-0.17	-27.78	-1.21	-33.77	-1.2	-43.2	-0.75	-25.4	-1.7	-34.74	-1.26
Secondary structure agreement	64.50%	-2.61	58.60%	-3.46	59.70%	-3.35	57.90%	-3.55	71.30%	-1.64	66.40%	-2.49
Solvent accessibility agreement	82.30%	0.59	79.50%	0.1	81.40%	0.47	80.30%	0.26	80.00%	0.23	79.90%	0.18
QMEAN6 score	0.729	-0.31	0.615	-1.48	0.651	-1.11	0.635	-1.26	0.677	-0.83	0.669	-0.95
dfire energy	-295.39	N/A	-295.55	N/A	-287.41	N/A	-284.59	N/A	-296.84	N/A	-307.8	N/A

Table S2. Molecular dynamics improves protein structural model quality. We report structural model quality scores for ancestral RLR RDs bound to blunt-ended and 5'ppp dsRNA, both before and after model refinement by molecular dynamics (see Methods).

Supplementary References

1. Kanehisa M: **The KEGG database**. *Novartis Found Symp* 2002, **247**:91-101; discussion 101-103, 119-128, 244-152.

2. Meylan E, Curran J, Hofmann K, Moradpour D, Binder M, Bartenschlager R, Tschopp J: **Cardif is an adaptor protein in the RIG-I antiviral pathway and is targeted by hepatitis C virus**. *Nature* 2005, **437**(7062):1167-1172.

3. Bruns AM, Horvath CM: **Antiviral RNA recognition and assembly by RLR family innate immune sensors**. *Cytokine Growth Factor Rev* 2014, **25**(5):507-512.

4. Bruns AM, Horvath CM: **LGP2 synergy with MDA5 in RLR-mediated RNA recognition and antiviral signaling**. *Cytokine* 2015, **74**(2):198-206.

5. Rodriguez KR, Bruns AM, Horvath CM: **MDA5 and LGP2: accomplices and antagonists of antiviral signal transduction**. *J Virol* 2014, **88**(15):8194-8200.
